# PRMT5-mediated intron retention triggers innate and adaptive immunity against cancer

**DOI:** 10.1038/s44321-026-00499-1

**Published:** 2026-08-17

**Authors:** Wiktoria Blaszczak, Wojciech Barczak, Chuyue Zhang, Garret Rochford, Annalisa Nicastri, Claire Hutchings, Anastasia Samsonova, Alexander Kanapin, Nicola Ternette, Paul Klenerman, Nicholas B La Thangue

**Affiliations:** 1 Laboratory of Cancer Biology, Department of Oncology, University of Oxford, Oxford, UK; 2 The Jenner Institute, Nuffield Department of Medicine, University of Oxford, Oxford, UK; 3 Peter Medawar Building for Pathogen Research, University of Oxford, Oxford, UK; 4 Medical Science Division, University of Oxford, Oxford, UK; 5 Translational Gastroenterology and Liver Unit, Nuffield Department of Medicine, Experimental Medicine, University of Oxford, Oxford, UK

**Keywords:** Cancer, Post-translational Modifications & Proteolysis

## Abstract

PRMT5 is expressed at high levels in many cancers, where it regulates diverse cellular pathways that contribute to oncogenesis. Here, we have defined a new role for PRMT5 in regulating and coordinating the interplay between the innate and adaptive immune response. This occurs, in part, through the influence of PRMT5 and E2F1 on RNA splicing and the presence of retained introns (RIs). We found that RIs have a propensity to form double-stranded RNAs that contribute to the innate response. Furthermore, many RIs contain non-canonical open-reading frames (ncORFs), which can be translated and then processed into small peptides that assemble with the MHC class I complex. Significantly, RI-derived peptides are highly immunogenic and, as a murine cancer vaccine, carrying a string of antigenic RI peptides, delayed tumour growth and enhanced survival. RIs are present in human tumour cells, and we identified T lymphocytes in human cancer patients, with antigen specificity for RI-derived peptides, that killed human tumour cells in vitro. Regulating intron retention thus offers a new therapeutic approach to enhance tumour immunogenicity.

## Synopsis

PRMT5-regulated intron retention generates non-canonical MHC class I antigens that drive anti-tumour immunity and can be exploited for cancer vaccine development.

- PRMT5 was found to regulate retained intron (RI)-derived transcripts with the propensity to form double-stranded RNA that contribute to the innate interferon response.
- The identified retained introns were shown to contain non-canonical open reading frames that are translated and processed into MHC class I-presented peptides.
- A vaccine containing RI-derived peptides (PepMInt) was demonstrated to delay tumour growth and prolong survival in a syngeneic mouse model.
- T lymphocytes reactive to RI-derived peptides were detected in colorectal cancer patients and were shown to kill tumour cells in vitro.

## Abstract (web summary)

PRMT5-regulated intron retention generates non-canonical MHC class I antigens that drive anti-tumour immunity and can be exploited for cancer vaccine development.

## Graphical abstract

**Figure:**
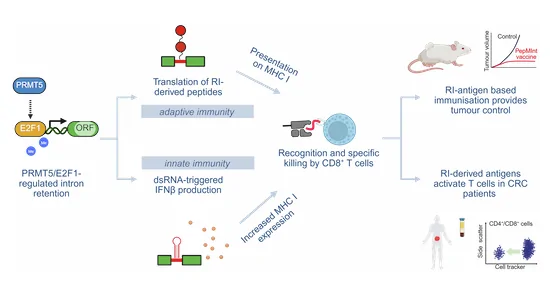

The paper explained **Problem**

Cancer vaccines offer a precise, low-toxicity alternative to conventional therapies, but their clinical utility has been constrained by the limited immunogenicity and patient specificity of tumour antigens. Current neoantigen-based vaccines rely on patient-specific somatic mutations, which restricts their applicability as the mutational burden varies widely both between tumour types and affected patients. In contrast, aberrant alternative RNA splicing is a hallmark of cancer. PRMT5 and E2F1, two oncogenic regulators deregulated in the majority of human cancers, regulate alternative RNA splicing and intron retention, which might give rise to a new class of non-canonical MHC class I antigens. This pathway therefore potentially represents a novel source of tumour antigens with the potential for broader application in cancer vaccine development.

**Results**

The authors show that the PRMT5-E2F1 axis regulates alternative RNA splicing and intron retention. The retained introns (RIs) were shown to form double-stranded RNA structures, which activate the innate immune response and trigger an interferon release contributing to increased expression of MHC class I. The authors further demonstrate that these RIs contain non-canonical open-reading frames that are translated into peptides and presented by MHC class I, marking cells for recognition by the adaptive immune response. The authors developed a RIs-derived, PepMInt (Peptide from Murine Introns) vaccine, which delayed tumour growth and improved survival in mice. Furthermore, T cells recognising RIs-derived peptides were detected in the blood of colorectal cancer patients, and further, the T cells were able to kill tumour cells in vitro.

**Impact**

These findings identify the PRMT5-E2F1 axis as a key regulator of RIs and describe a previously unrecognised source of immunogenic cancer antigens arising as a consequence of aberrant alternative splicing. The efficacy of the RI-based cancer vaccine, PepMInt, was confirmed in tumour-bearing mice, while T cells recognising RI-derived peptides were identified in human cancer patients. Together, these findings establish RIs as a promising source of cancer antigens that could find clinical utility in human cancers with deregulated PRMT5-E2F1 signalling.

## Introduction

Alternative RNA splicing (AS) is a widespread mechanism which allows cells to control post-transcriptional RNA diversity (Berget et al, 1977; Nilsen and Graveley, 2010; Ule and Blencowe, 2019). RNA splicing is a highly regulated process, involving the activity of the core spliceosome and an extensive collection of splicing factors (SF). Previous studies found that arginine methylation mediated by PRMT5 enables E2F1 to regulate a diverse group of genes at the level of alternative RNA splicing, in addition to the classical transcription-based mechanism attributed to the E2F pathway control (Bate-Eya et al, 2025; Roworth et al, 2019; Sachamitr et al, 2021). At a mechanistic level, arginine methylation facilitates interactions between E2F1 and components of the splicing machinery, which together with the direct methylation of SF, expands the repertoire of splicing events under E2F1 control (Roworth et al, 2019).

Moreover, AS is important in regulating many biological processes and is often deregulated in diseases, including cancer (Arif et al, 2023; Climente-Gonzalez et al, 2017; Guo et al, 2012; Venables et al, 2008). Aberrant AS events can arise in cancer through several mechanisms, resulting in RNA variants which encode proteins that contribute to the malignant phenotype (Ouyang et al, 2021). These include exon skipping or inclusion events, in addition to RNA carrying unspliced introns, referred to as retained introns (RIs; Hammarskjold, 1997; Ner-Gaon et al, 2004). RIs are a relatively poorly defined type of splicing structure that has been described in a variety of cellular contexts (Ner-Gaon et al, 2004; Pan et al, 2008). RIs frequently differ both qualitatively and quantitatively between normal and cancer cells, where RIs are generally rarer in normal compared to cancer cells (Dvinge and Bradley, 2015). It remains unclear whether the presence of RIs reflects a compromised splicing programme or a regulated splicing event with a defined biological role (Jacob and Smith, 2017).

Double-stranded RNA (dsRNA) can be a product of viral replication, which in mammalian cells is sensed to signal that cells are undergoing a detrimental infection event (Weber et al, 2006). The process is mediated by cytoplasmic sensor proteins that detect dsRNA and activate a cascade of events leading to the interferon response, which, with other cytokines, restricts virus growth and spread, usually culminating in cell death (Estornes et al, 2012; Scarim et al, 2001). The interferon (IFN) system is a key component of the innate immune response, regarded as the first line of cellular defence, and is highly relevant to cancer cells, where it causes growth arrest and apoptosis, and alters immunological recognition (Acha-Sagredo et al, 2025; Benci et al, 2019).

The major histocompatibility (MHC) class I complex plays a central role in the adaptive immune response by presenting peptide antigens bound to the MHC to the T-cell receptor (TCR), which invokes an antigen-specific T cell immune response (Heilig and Tonegawa, 1986). In humans, the MHC class I gene locus is highly polymorphic, with each allele able to selectively bind to specific sets of peptides (Dausset, 1958; Payne et al, 1964). In previous studies, we identified an extensive repertoire of MHC class I-associated peptides on cancer cells derived from diverse genes, including an unexpected group of peptides encoded by poorly annotated genes like lncRNA genes. These lncRNA genes were found to be under transcriptional control by the pRb-E2F pathway, and the MHC-bound peptides were highly immunogenic (Barczak et al, 2023).

Here, we describe a novel role for PRMT5 in coordinating the interplay between the innate and adaptive immune response. Mechanistically, this occurred, in part, through the effect of PRMT5 inhibition and E2F1 on the RNA splicing programme, which, amongst other effects, altered the repertoire of RIs present in cancer cells. We found that many RIs exhibit a propensity to form dsRNA structures, which then act at the biological level to induce the interferon response, which coincides with increased MHC class I expression. Notably, RIs were shown to contain non-canonical open-reading frames (ncORFs; (Deutsch et al, 2026)) which encode polypeptides that can be processed to small peptides that assemble with the MHC class I complex. Most significantly, RI-derived peptides were found to be immunogenic and, as a vaccine, drove a potent adaptive antigen-specific T-cell response which delayed tumour growth and enhanced survival in mouse models. We identified T lymphocytes in human cancer patients with antigenic specificity for peptides derived from human RIs, capable of killing human tumour cells in vitro. Our results establish an unexpected interplay between the innate and adaptive immune response, orchestrated by PRMT5 through its influence on the presence of RIs. Because intron retention potentially signals an aberrant and possibly incomplete RNA splicing event, our results not only define a new therapeutic approach for regulating the immunogenicity of cancer cells but also highlight an immune surveillance mechanism capable of recognising and eliminating damaged cells undergoing abnormal splicing.

## Results

### RIs in human tumour cells

Residue-specific arginine methylation by PRMT5 enables E2F1 to influence many genes at the level of alternative RNA splicing (AS) (Barczak et al, 2023; Carr et al, 2026). We reasoned therefore that the interplay between PRMT5 and E2F1 may impact RIs and evaluated RI events in human HCT116 cells by inspecting RNA-seq datasets derived from WT E2F1 and E2F1 CRISPR HCT116 cells (E2F1Cr), treated with the selective PRMT5 inhibitor, T1-44 (Barczak et al, 2023; Barczak et al, 2020). We performed differential analysis using two algorithms, the VAST tool which uses a Bayesian probabilistic model focused on splicing events and transcript isoform abundance (Tapial et al, 2017) and rMATS which uses a statistical framework based on replicate samples that predicts differential splicing analysis (Shen et al, 2014). A variety of differentially regulated AS events were detected, including alternative 3’ and 5’ splice site selection (ALT5 and ALT3), skipped exons (ES), and RIs (Appendix Fig. S1A(i–iii); Dataset EV1).

**Figure 1:**
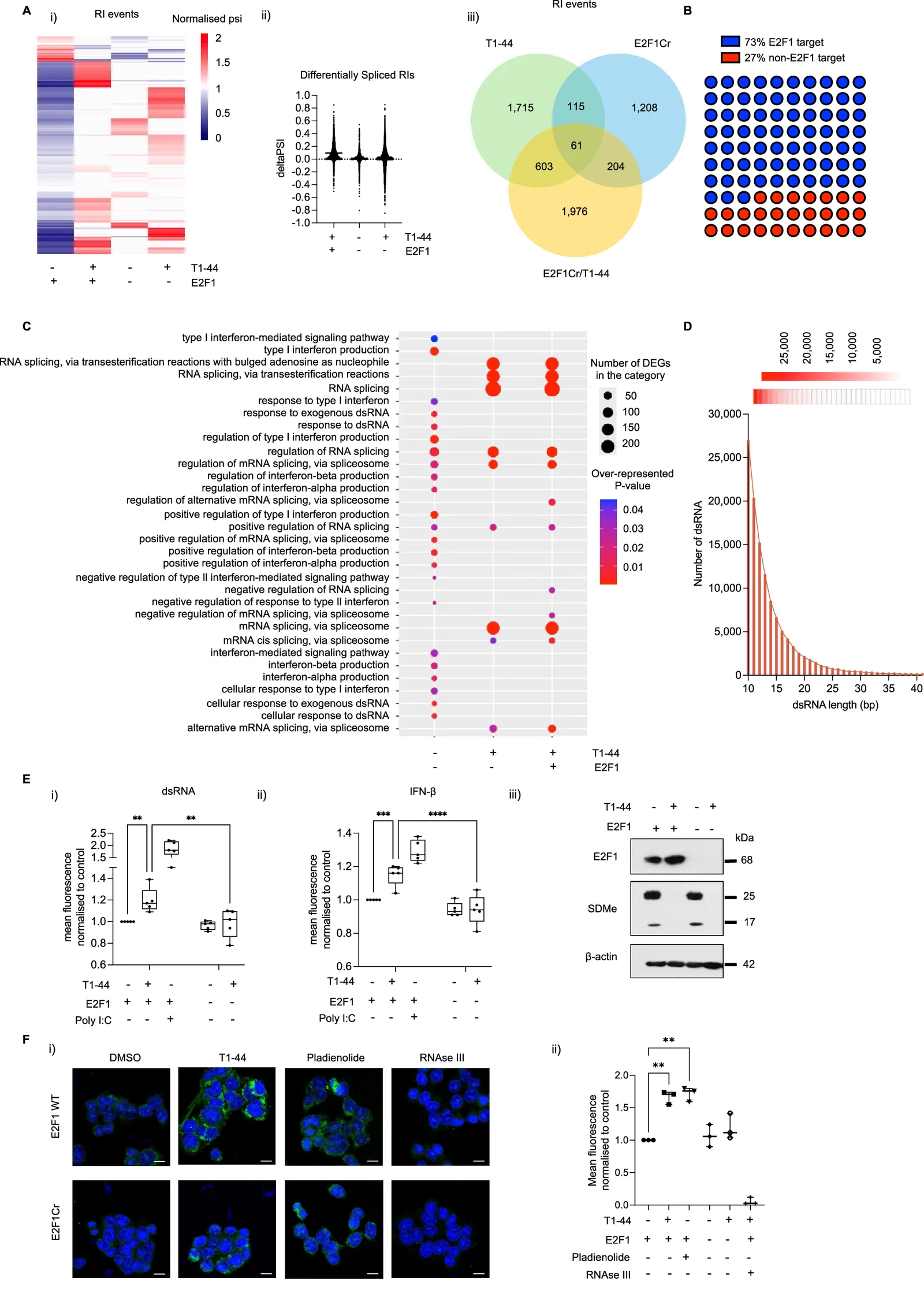
Analysis of retained introns in HCT116 cells. (**A**) Differential analysis of RIs using the VAST tool on RNA-seq datasets derived from HCT116 WT E2F1 and E2F1Cr cells treated with T1-44 for 48 h. (i) Results presented as a heatmap of normalised psi values for differentially spliced RIs; *P* value < 0.05, Bayesian Inference; Red—higher psi; Blue—lower psi (please see Heatmap generation and normalisation section in “Methods”); (ii) Results of the differentially upregulated and downregulated RI events in different conditions presented as a delta PSI plot; *P* value < 0.05, Bayesian Inference; (iii) Overlap of differentially spliced RIs in different conditions; *P* value < 0.05, Bayesian Inference. (**B**) Percentage of RI genes differentially regulated at a statistically significant level (VAST tool; *P* value  <  0.05) in the T1-44 treatment condition as compared to DMSO-treated WT E2F1 HCT116 that score as potential E2F1 target genes. (**C**) Gene ontology analysis was performed using the goseq R package on the differentially expressed genes (*P* value < 0.05) generated from each of the RNA-seq datasets. GO biological processes and *P* value < 0.05 cut-off (Wallenius noncentral hypergeometric test) were used for pathway enrichment. (**D**) ViennaRNA algorithm prediction of dsRNA from RI transcripts (combined from all treatments, i.e., T1-44, E2F1Cr, E2F2Cr/T1-44) identified by the VAST tool algorithm. The graph and heatmap represent the numbers of dsRNA fragments produced by RI loci as a function of their length (in bp). (**E**) Quantitation of dsRNA (i) and IFN-β (ii) intracellular staining (measured by flow cytometry) in HCT116 E2F1 WT and E2F1Cr cells treated with DMSO or T1-44. Mean fluorescence was normalised to DMSO-treated WT cells (control) and presented as a Box and Whiskers plot. Box and whiskers are defined as the minimum, first quartile, median, third quartile, and maximum of the data. In total, 2 μg/mL Poly I:C was transfected to WT cells as a technical positive control. *n* = 5; *P < 0.05, **P < 0.01, ****P < 0.0001, one-way ANOVA; (**E**) (i) E2F1 WT DMSO vs. E2F1 WT T144—*P* value = 0.0033, E2F1 WT T144 vs. E2F1 Cr T144—*P* value = 0.0019; (**E**) (ii) E2F1 WT DMSO vs. E2F1 WT T144—*P* value = 0.001, E2F1 WT T144 vs. E2F1 Cr T144—*P* value < 0.0001; (iii) A representative immunoblot showing E2F1Cr and T1-44 activity by SDMe mark is also shown. (**F**) Representative example images (i) and quantification (ii) of dsRNA intracellular staining (measured by immunofluorescence) of HCT116 E2F1 WT and E2F1Cr cells treated with DMSO or T1-44. Mean fluorescence was normalised to DMSO-treated WT cells (control). Data are presented as the median, with whiskers indicating the minimum and maximum values. Cells were also treated with pladienolide as a technical positive control, and RNAse III treatment was used as a negative control. Original magnification: ×40, scale bar = 20 μm; *n* = 3; **P < 0.01, one-way ANOVA; (**F**) (ii) E2F1 WT DMSO vs. E2F1 WT T144—*P* value = 0.0027, E2F1 WT DMSO vs. E2F1 WT Pladienolide—*P* value = 0.0057. Source data are available online for this figure.

Both algorithms identified RI events (*P* value < 0.05 for the VAST tool and FDR < 0.01 for rMATS) associated with E2F1 status and PRMT5 inhibition, with the effect of PRMT5 inhibition being more pronounced in the presence of E2F1 (Fig. 1A; Appendix Figs. S1B–E and S2A–C; Dataset EV1). RI events were seen to be dependent on E2F1 and PRMT5, since they appeared in the combined E2F1Cr/T1-44 treatment condition, whereas other RIs were dependent on single treatment conditions (Fig. 1A; Appendix Figs. S1B–E and S2A–C). In each condition, the majority of RIs were derived from E2F target genes (Fig. 1B; Appendix Figs. S1E and S2D). We also addressed whether the function of the genes, differentially expressed upon T1-44 treatment (Barczak et al, 2020), were connected through biological terms by gene ontology (GO), and identified interferon and RNA splicing as the significantly enriched terms (Fig. 1C). Further, there was a role for both E2F1 and PRMT5 in the GO analysis as the enriched terms were altered in T1-44, E2F1Cr, and E2F1Cr/T1-44 treatment conditions (Fig. 1C).

We selected a group of candidate RNAs containing a retained intron, where intron retention was influenced by E2F1 and PRMT5 (Appendix Fig. S3A); these were DDX55, CDK10, A1BG, HDAC6 and SPIN2B (Appendix Fig. S3B). We addressed whether the RI was regulated in conditions where the level of E2F1 and PRMT5 had been manipulated, and measured intron inclusion relative to intron exclusion. Intron inclusion increased upon PRMT5 inhibition, which was dependent on E2F1, as the enhanced intron inclusion was reduced when E2F1Cr cells were treated with the T1-44 inhibitor (Appendix Fig. S3A). These results support a role for E2F1 and PRMT5 in the regulation of RIs in cancer cells.

### Intron retention in murine tumour cells

We progressed on to examine RIs in CT26 cells to assess whether the principles established in human cells were relevant in murine cancer cells. Again, we observed that the major types of AS events are regulated upon T1-44 treatment, with RIs representing the most abundant one (Appendix Fig. S4A(i–iii); Dataset EV2). We found that the RIs identified in CT26 cells were regulated in a similar manner to those in human cells by PRMT5 inhibition (Appendix Fig. S4B,C). Furthermore, many of the RNAs that contain RIs were derived from E2F target genes (Appendix Fig. S4D). Importantly, when analysing the biological terms connected to the differentially expressed genes (Barczak et al, 2023), significantly enriched terms included the interferon response, RNA splicing and viral dsRNA (Appendix Fig. S4E(i–ii)), which were similar to the GO terms highlighted in the human cell analysis (Fig. 1C).

### RIs form double-stranded RNA structures, which drive an interferon response

RIs have been described in numerous biological contexts, although their precise role remains unclear (Jacob and Smith, 2017). To shed light on this issue, we tested whether the RIs detected in PRMT5 inhibitor-treated cells had a propensity to form double-stranded RNA structures. This is because dsRNA structures are potentially immunomodulatory through the ability to induce an interferon (IFN) response and further enhance MHC expression (Carre et al, 2025). We examined this possibility using an algorithm for RNA secondary structures, namely ViennaRNA package (Lorenz et al, 2011), which predicted that a large proportion of the RIs (between 10 and 20 base pairs long) were theoretically capable of forming stable dsRNA structures (Fig. 1D). In addition, using the method described in Trotta et al (Trotta, 2014) we estimated the prevalence and predicted stability of structures within the RIs using Adjusted Minimum Free Energy (AMFE) namely the minimum folding free energy of an RNA sequence normalised to a standard length, typically per 100 nucleotides, allowing structural stability comparisons between RNAs of different sizes. These results (Appendix Fig. S4F) demonstrated that the AMFE values for the RIs defined in our study were significantly lower (that is, a more stable dsRNA structure) than the value for randomly shuffled RNA sequences (length from 12 to 600 nt) (AMFE ~ −32 kcal/mol. These results suggest that the secondary structures originating from RIs are more stable than random sequences and thus they probably contribute to the pool of dsRNAs we observe in T1-44-treated cells.

We then measured dsRNA structures directly in cells by immunostaining with the J2 antibody, which is a validated antibody tool for dsRNA detection (Bonin et al, 2000; Lopez-Polo et al, 2024; Weber et al, 2006); it should be noted, however, that J2 does not distinguish between dsRNA derived from RIs or other sources. Structural analyses have demonstrated that the J2 monoclonal antibody recognises double-stranded RNA as short as 14 bp in a sequence-independent manner by binding the A-form helical backbone of extended RNA duplexes, enabling detection of diverse dsRNA, including retained intronic dsRNA structures (Bou-Nader et al, 2025). There was a significant increase in immunostaining in WT HCT116 cells treated with T1-44 compared to the DMSO treatment control (Fig. 1E(i,iii),F); the same effect was also observed in other cancer cell lines (Appendix Fig. S4G). Moreover, this effect of T1-44 was lost in E2F1Cr cells, where the increased J2 immunostaining was no longer apparent (Fig. 1E(i),F). Further, the J2 signal was absent when T1-44-treated cells were co-treated with RNAse III (Fig. 1F), and similarly, enhanced J2 immunostaining occurred in cells treated with pladienolide, which is a splicing inhibitor that increases the level of RIs (Zhang et al, 2020) or Poly I:C (Fig. 1E,F). We then assessed IFN-β levels and observed, most significantly, that the increased level of J2 immunostaining coincided with the level of IFN-β produced by HCT116 cells (Fig. 1E(ii)), which was also reduced in the E2F1Cr cells (Fig. 1E(ii)). Moreover, to confirm that dsRNA-sensing pathways contribute to the increased IFN-β levels, we treated cells with an siRNA against Protein Kinase R (PKR), which is a key dsRNA sensor that activates the IFN response (Cottrell et al, 2024; Zheng and Bevilacqua, 2004). We observed that IFN-β induction upon T1-44 treatment occurred only in cells with active PKR, since it was absent in siRNA-treated cells (Appendix Fig. S4H), which is consistent with dsRNA sensing by PKR leading to increased IFN-β levels.

**Figure 2:**
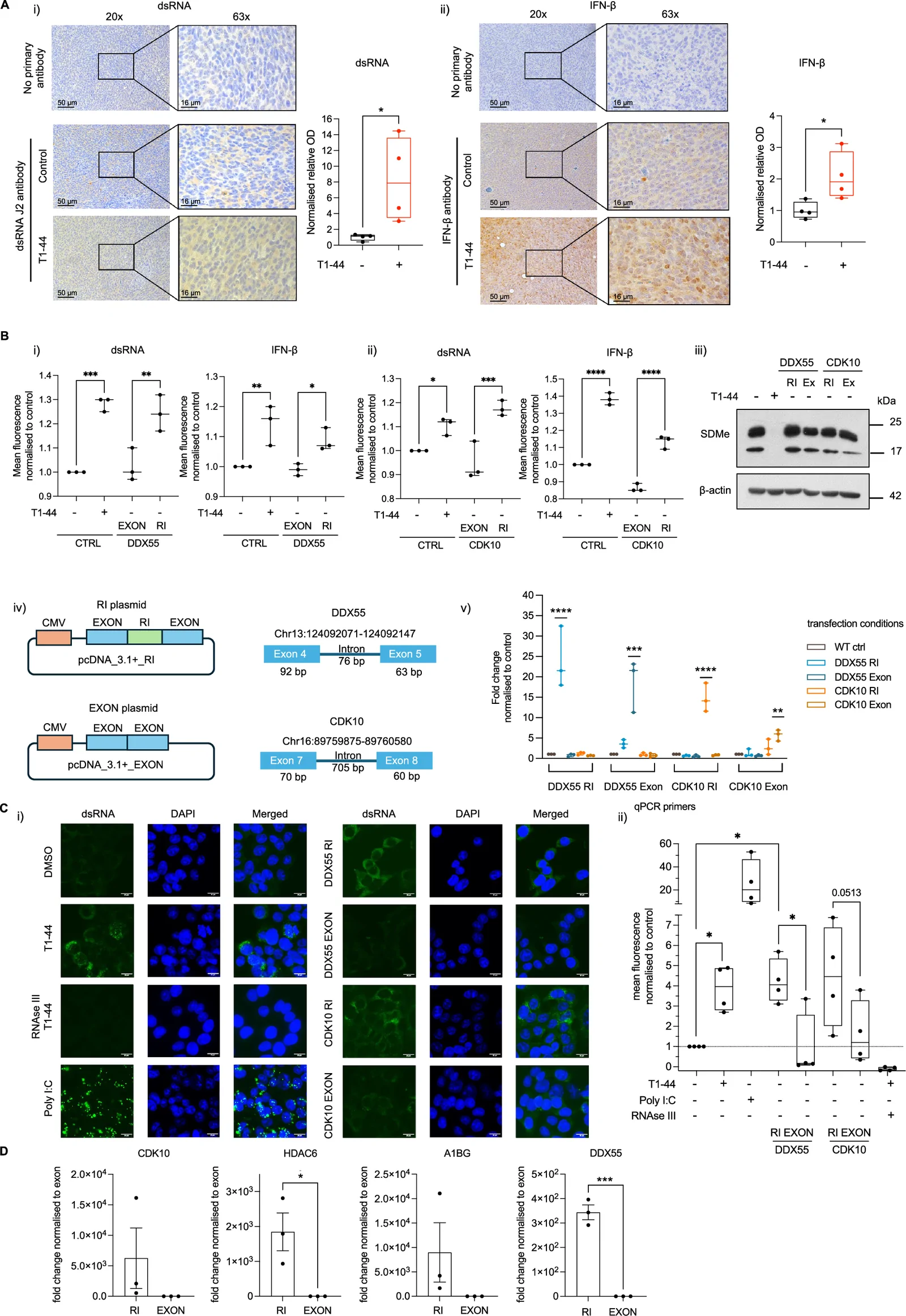
RIs form double-stranded RNA structures, which drive the interferon response. (**A**) An example of immunohistochemical staining of dsRNA (i) and IFN**-**β (ii) on Colon26 tumour tissue; Original magnification: ×20, scale bar, 50 μm; and ×63; scale bar, 16  μm. A slide without primary antibody treatment was used as a negative control for staining. Normalised optical density (OD) of each slide was analysed from a different biological replicate and presented as a Box and Whiskers plot. Box and whiskers are defined as minimum, first quartile, median, third quartile, and maximum of data; *n* = 4; *P < 0.05, unpaired Student’s *t* test; (**A**) (i) Control vs T144—*P* value = 0.0344; (**A**) (ii) Control vs T144—*P* value = 0.0354. (**B**) dsRNA and IFN-β intracellular staining of HCT116 WT cells transfected with either RI or Exon expression vector (iv) representing DDX55 (i) or CDK10 (ii). The graphs show the RI vector fluorescence signal relative to control (Exon vector-transfected cells). Data are presented as the median, with whiskers indicating the minimum and maximum values. T1-44 treatment was used as a positive control. *n* = 3; *P < 0.05, **P < 0.01, ***P < 0.001, ****P < 0.0001, using one-way ANOVA; (**B**) (i) dsRNA CTRL DMSO vs CTRL T144—*P* value = 0.0003, dsRNA DDX55 EXON vs DDX55 RI—*P* value = 0.0018, IFN-β CTRL DMSO vs CTRL T144— *P* value = 0.0044, IFN-β DDX55 EXON vs DDX55 RI—*P* value = 0.0343; (**B**) (ii) dsRNA CTRL DMSO vs CTRL T144—*P* value = 0.0487, dsRNA CDK10 EXON vs CDK10 RI—*P* value = 0.0006, IFN-β CTRL DMSO vs CTRL T144—*P* value < 0.0001, IFN-β CDK10 EXON vs CDK10 RI—*P* value < 0.0001; (iii) A representative Immunoblot showing the activity of T1-44 by SDMe mark and E2F1 level. Actin was used as a loading control; (iv) Diagram of RI and exon cloning strategy. The RI sequence and flanking exons were cloned into the pcDNA 3.1(+) plasmid vector. (v) qPCR relative expression of RI and Exon transcripts confirming transfection of DDX55 and CDK10 plasmids, compared to cells transfected with control plasmid (CTRL). *n* = 3; *P < 0.05, **P < 0.01, ****P < 0.0001, one-way ANOVA; DDX55 RI-WT ctrl vs DDX55 RI—*P* value < 0.0001, DDX55 EXON-WT ctrl vs DDX55 EXON—*P* value = 0.0002, CDK10 RI-WT ctrl vs CDK10 RI—*P* value < 0.0001, CDK10 EXON-WT ctrl vs CDK10 EXON—*P* value −0.003. Data are presented as the median, with whiskers indicating the minimum and maximum values. (**C**) (i) Representative immunofluorescence images of dsRNA in HCT116 cells transfected with RI or Exon plasmids of DDX55 or CDK10. Cells were treated with RNase III as a negative control or transfected with 2 μg/mL Poly I:C as a positive control. Original magnification: ×40, scale bar = 20 μm. (ii) Quantification of immunofluorescence staining shown in (**A**). Mean fluorescence was calculated using Fiji software. Results were normalised to control transfected cells treated with DMSO (first treatment column). *n* = 4; *P < 0.05, one-way ANOVA; DMSO vs T144—*P* value = 0.0338, DMSO vs DDX55 RI—*P* value = 0.0153, DDX55 RI vs DDX55 EXON—*P* value = 0.0135. Data presented as a Box and Whiskers plot. Box and whiskers are defined as the minimum, first quartile, median, third quartile, and maximum of the data. (**D**) qPCR analysis of the indicated RI transcripts after dsRNA immunoprecipitation (with J2 antibody) of in vitro transcribed RI or exon vectors presented as RI fold enrichment over exon using Retained Intron (RI) or Exon spanning primers (EXON); *n* = 3, log10 scale. *P < 0.05, ***P < 0.001, two-tailed, unpaired Student’s *t* test; HDAC6 RI vs EXON—*P* value = 0.0271, DDX55 RI vs EXON—*P* value = 0.0003. Data are presented as mean ± SEM. Source data are available online for this figure.

We similarly addressed J2 immunostaining in murine tumours treated with T1-44, where we tested for dsRNA and IFN-β levels in colon26 tumours (Barczak et al, 2023). We observed increased levels of J2 immunostaining in treated but not untreated tumours, with a similar staining pattern apparent for IFN-β (Fig. 2A; Appendix Fig. S4I). Notably, the increased dsRNA and IFN-β level occurred in conditions of inhibition of tumour growth upon T1-44 treatment and enhanced T-cell infiltration within the tumour microenvironment (Barczak et al, 2023), suggesting that splicing disruption and innate immune activation also contribute to anti-tumour immunity.

We evaluated whether the gene-specific RIs can form dsRNA structures in cells (Fig. 2B,C; Appendix Fig. S4J). To do this, we introduced the RI sequence into an expression vector and measured the level of dsRNA and IFN-β by immunostaining in transfected cells; for the control transfection, we expressed the surrounding exons (without the intron sequence) (Fig. 2B(iv)). When the RI derived from DDX55 was expressed, we observed a significant increase in both dsRNA and IFN-β levels compared to the exon-only vector (Fig. 2B(i),C). A similar increase in J2 and IFN-β immunostaining for the RI compared to the exon-only expression was also seen in CDK10-transfected cells (Fig. 2B(ii),C). The J2 and IFN-β levels remained unchanged in DMSO-treated cells in contrast to T1-44-treated cells, and the addition of RNAse III to T1-44-treated cells reduced the J2 immunostaining to negligible background levels (Fig. 2C). Cells treated with poly I:C served as a positive control for dsRNA detection (Fig. 2C).

We measured the level of ectopic RI and exon-only expression RNAs by qPCR in cells transfected with DDX55 and CDK10 vectors, where increased RI and exon RNA levels coincided with the appropriate transfected vector (Fig. 2B(v); Appendix Fig. S4I). Further, we used the J2 antibody to immunoprecipitate dsRNA after in vitro transcription (Fig. 2D), where we observed a thousand-fold increase in dsRNA with the RI vector compared to exon-only (Fig. 2D). Importantly, we also performed a J2 immunoprecipitation on endogenous RNA, which confirmed that CDK10 and DDX55 RI-containing transcripts form dsRNA structures in cells (Appendix Fig. S4K). Collectively, these results support the model in which RIs contribute to dsRNA formation and innate immune activation.

### The innate immune response increases MHC class I expression

The enhanced dsRNA seen in cells with RIs coincided with an IFN response (Fig. 2). It was of further interest therefore to address whether the innate response could influence components of the adaptive immune response, such as the level of MHC class I. To test this possibility, we assessed the effect of IFN-β on MHC class I surface expression in HCT116 cells. We found that treatment with IFN-β led to a fivefold increase in MHC surface expression, and this effect was abolished when IFN receptors were blocked with an IFN alpha/beta receptor subunit 1 antibody (Appendix Fig. S4L). A similar increase in MHC class I expression was apparent in HCT116 cells treated with T1-44 (Appendix Fig. S4M), which enhanced IFN-β production via its effects on RI and dsRNA formation. To determine whether the T1-44-mediated increase in MHC class I expression was dependent on a secreted factor released into the medium, we tested the effect of T1-44-conditioned medium on surface MHC class I levels and observed a corresponding increase in expression following the addition of the medium taken from T1-44-treated cells to untreated cells (Appendix Fig. S4N). As the enhanced MHC class I expression is highly likely to result from the action of IFN-β and thereby improve antigen presentation and the adaptive immune response, we progressed on to address whether there was a role for RIs in antigen presentation.

### Non-canonical ORFs in RIs encode MHC class I-bound peptides

**Figure 3:**
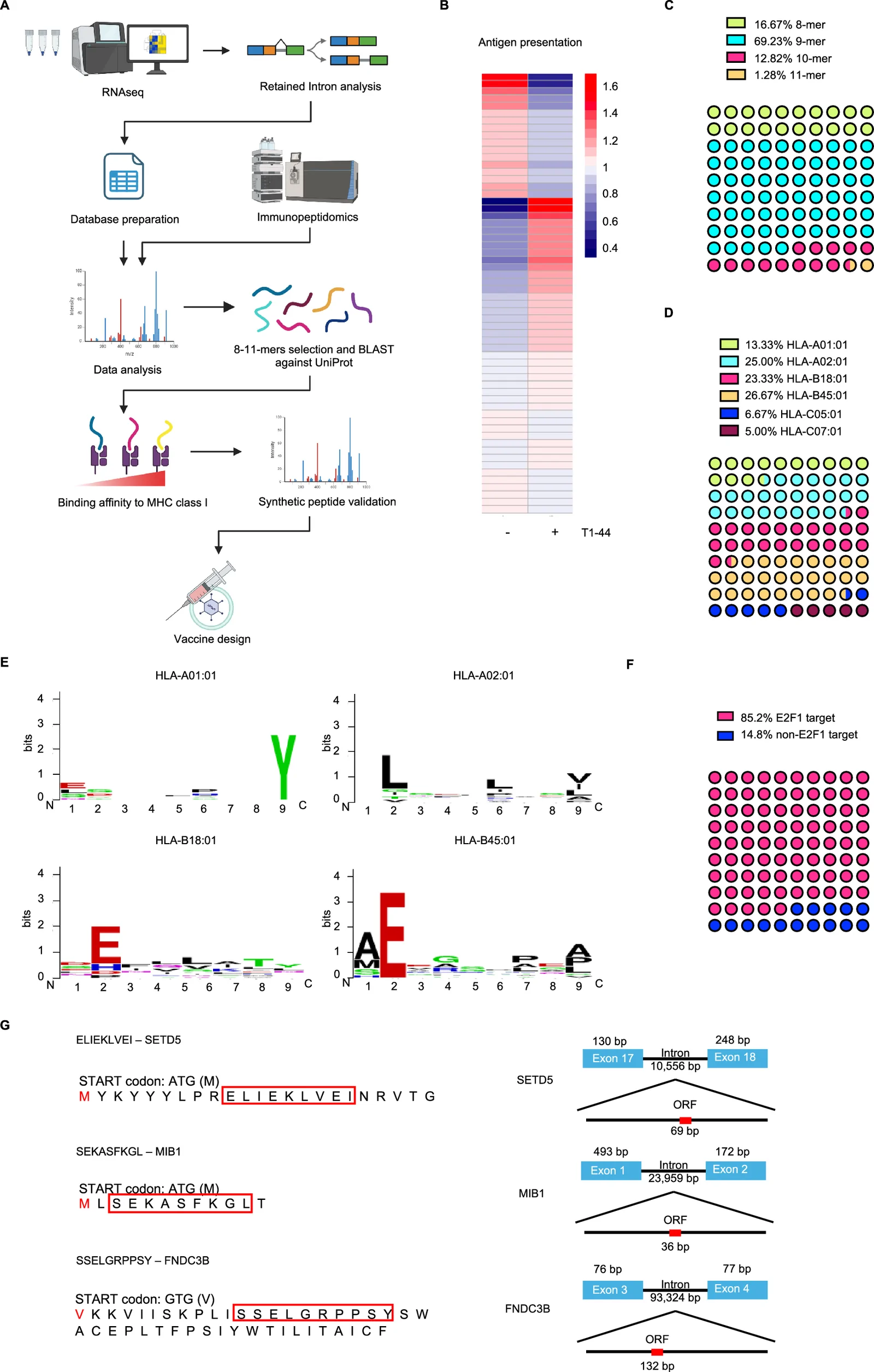
Immunopeptidomics analysis of human HCT116 cells. (**A**) Workflow describing the immunopeptidomics analysis. The RNA-seq datasets were analysed with the VAST tool algorithm, and differentially spliced RIs were translated into predicted peptide sequences to build FASTA databases. The databases were then aligned with the immunopeptidomics library, and the results were narrowed down with the following criteria: selection of 8-11-mers, BLAST (no overlap with the known peptide sequences in UniProt/SwissProt database (https://blast.ncbi.nlm.nih.gov/Blast.cgi), prediction of binding to MHC class I (https://services.healthtech.dtu.dk/services/NetMHCpan-4.1/), and peptide validation (spectra comparison of endogenous and synthetic peptides). (**B**) Heatmap of RI-derived peptide abundance from the quantitative immunopeptidomics analysis (60 peptides) (both upregulated and downregulated in T1-44 treatment vs DMSO). Relative abundance values were plotted. Red, upregulation; blue, downregulation; white, no change in abundance. *n*  =  2 independent experiments (each with two technical replicates). (**C**) The peptide length of each human RI-derived peptide is displayed as a percentage of the total. Sixty quantifiable peptides in total (pooled from two independent experiments) were detected by PROGENESIS software (data derived from immunopeptidomics experiments performed in biological independent replicates, each with two technical replicates). (**D**) Predicted MHC allele frequency for identified RI-derived peptides displayed as a percentage of total peptides. (**E**) Sequence logos of amino acid residue conservation in RI-derived peptides for each MHC allele. (**F**) The percentage of corresponding RI genes giving rise to MHC class I-bound peptides that score as potential direct E2F1 target genes. Analysis was performed on all 54 unique peptide-coding RI-containing genes identified. (**G**) Examples of RI-derived open-reading frames are displayed. The predicted ORF (left panel) gives rise to the identified MHC class I-bound peptide (boxed in red). Potential start residues are highlighted in red text. Graphical representation of predicted ORF position within the intron of the gene is presented (right panel). Source data are available online for this figure.

Although introns are generally regarded to be non-coding genomic sequences, we reasoned that RIs could, theoretically, contain non-canonical open-reading frames (ncORFs) that may contribute to the MHC class I peptidome. We therefore interrogated our immunopeptidome library (Barczak et al, 2023) to ascertain whether peptides derived from RIs were present in the repertoire displayed by the human MHC class I complex. To this end, we generated a database of all possible reading frames for every RI detected in the RNA-seq dataset. The peptides in the library (Dataset EV3) were then aligned with the reading frame database and narrowed down by applying selection criteria (as described in Fig. 3A), enabling us to identify 60 quantifiable peptides derived from RI sequences with a mode size of 9 residues (Fig. 3B,C; Dataset EV4). In addition, 10,796 canonical and 76 previously described lncRNA-derived (Barczak et al, 2023) peptides were detected, with similar signal intensity across all groups (Appendix Fig. S5A). Individual RI-derived peptides had the sequence of residues confirmed by comparing the immunopeptidomics spectrum to its synthetic peptide counterpart spectrum (Appendix Fig. S5B). Further, conserved residues in the peptides were consistent with those required for efficient human HLA MHC class I binding (Reynisson et al, 2020) (Fig. 3D,E), and were predicted to have high affinity for human HLA-A, -B, and -C MHC class I proteins (Fig. 3D,E), reflecting the HLA haplotype of HCT116 cells (Adams et al, 2005). Quantitative analysis of each peptide indicated that 36.7% of the peptides were upregulated (by more than 20%), 21.7% downregulated (by more than 20%) and 41.6% unchanged upon treatment with T1-44 (Fig. 3B). Significantly, over 85% of the genes encoding the RI peptide sequence were E2F targets (Fig. 3F), an observation which is consistent with the different level of RIs apparent between E2F1 WT and Cr cell conditions (Fig. 1B). Further, we identified ORFs for each peptide within the sequence of each RI (examples shown in Fig. 3G). Generally, we observed that the human peptides were derived from small ncORFs (mostly less than 100 codons) within each RI, and typically had a weak translation-initiating sequence with poor alignment with the classical sequence (Kozak, 1986), with a similarly divergent translation-initiating codon (Appendix Fig. S5C). We therefore regard these small ORFs as non-canonical, as they lack the typical conserved features that characterise and direct translation of classical protein-encoding genes (Chong et al, 2020; Kina et al, 2025).

We also measured the intron inclusion/exclusion ratio of a selection of human RIs that encode MHC class I-bound peptides (Appendix Fig. S5D). We found that PRMT5 inhibition altered the level of the RIs, increasing intron retention in genes like RSPRY1 or NRC2C, and decreasing it in ATAD5 and CDKAL1. When the intron/exon ratio was compared between WT and E2F1Cr cells, SETD5 RI was increased, and CDKAL1 was at lower levels, thus confirming a role for E2F1. In many cases, the effect of PRMT5 inhibition on the RI was dependent on the presence of E2F1, with a reduced impact in E2F1Cr cells (for example, in MIB1, ZNF592, KLHL29, FNDC3B, FAM188B, ARID1B, SCHIP1, Appendix Fig. S5D). Moreover, RI transcripts including MIB1, SETD5, FNDC3B, ATAD5, and FAM188B were enriched in the polysome translating fraction of ribosomes (Appendix Fig. S5E(ii,iv)), which is consistent with these ncORFs using the central protein translation machinery (Appendix Fig. S5E(iii,iv)). Consistent with these findings, our polysome profiling RNA-seq demonstrated read density across the majority of ncORFs present in RIs, with many showing increased density upon PRMT5 inhibition. This indicated that RIs not only accumulate but are actively translated in cells (Appendix Fig. S5F(i–iv)).

Interestingly, an analysis of publicly available dsRNA RIP-seq datasets (Zhang et al, 2022b) revealed that the vast majority of RI encoding peptide sequences (identified in our study) were enriched in dsRNA immunoprecipitates (Appendix Fig. S5G(i–v)). This finding indicates that most peptide-coding RIs can form double-stranded RNA structures in cells, further supporting the role of intron retention and dsRNA formation in activation of innate immune pathways.

**Figure 4:**
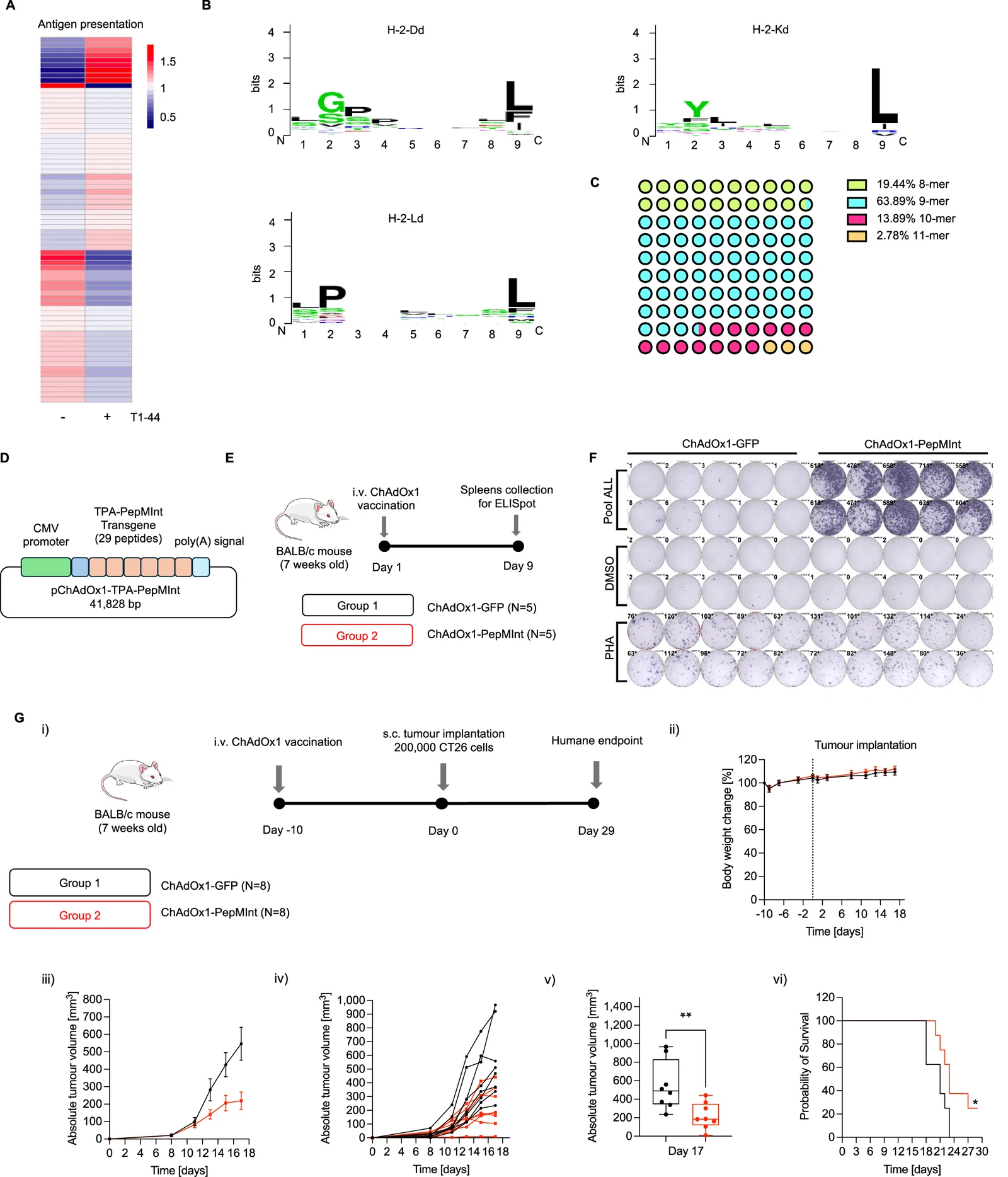
Immunopeptidomics analysis of murine CT26 cells. (**A**) Heatmap of RI-derived peptide abundance from the quantitative immunopeptidomics analysis (72 peptides, both upregulated and downregulated in T1-44 treatment vs. DMSO CT26 cells). Red, upregulation; blue, downregulation; white, no change in abundance. *n*  =  2 independent experiments (each with two technical replicates). (**B**) Sequence logos of amino acid residue conservation in RI-derived peptides for each MHC allele. (**C**) The peptide length of each murine RI-derived peptide is displayed as a percentage of the total. Seventy-two quantifiable peptides in total (pooled from two independent experiments) were detected by PROGENESIS software (data derived from an immunopeptidomics experiment performed in two independent biological replicates, each with two technical replicates). (**D**) Diagram of ChAdOx1-PepMInt vector containing the cassette of 29 RI-derived peptides. (**E**) Schema of the immunogenicity experiment. Groups of 5 BALB/c mice were vaccinated with the ChAdOx1-PepMInt expressing the poly-antigen cassette containing the selected 29 RI-derived peptides, or a control ChAdOx1-GFP. On day 9 post vaccination, the mice were sacrificed and their splenocytes collected for the ELISpot assay. The experiment was performed twice with successful replication. (**F**) Splenocytes were stimulated with the pool of RI peptides (all), and their activity was measured by interferon-γ-based ELISpot. DMSO and PHA were used as negative and positive controls, respectively. This ELISpot is also presented in Appendix Fig. S9A as part of the same experiment. (**G**) (i) Groups of 8 BALB/c mice were vaccinated with ChAdOx1-PepMInt or ChAdOx1-GFP. At 10 days post-vaccination mice were subcutaneously injected with the 200,000 CT26 cells; (ii) Relative body weight of BALB/c mice presented as a mean value ± SEM; (iii) Absolute tumour volume is presented as mean ± SEM; (iv) Absolute tumour volumes are presented in the individual mice; (v) Absolute tumour volume of individual mice at day 17 (Student’s *t* test; **P value; ChAdOx1-GFP vs ChAdOx1-PepMInt—*P* value = 0.0083) presented as a Box and whiskers plot. Box and whiskers are defined as minimum, first quartile, median, third quartile, and maximum of data; (vi) Kaplan–Meier survival curve; *P value, Log-rank (Mantel-Cox) test; ChAdOx1-GFP vs ChAdOx1-PepMInt—*P* value = 0.043. The experiment was performed twice with successful replication (please see Appendix Fig. S10B,C and the Source data). Source data are available online for this figure.

We performed a similar analysis on murine CT26 cells, which utilised our annotated MHC class I immunopeptidomics peptide dataset derived from CT26 cells (Barczak et al, 2023), with a similarly prepared database containing translated polypeptides from all possible reading frames for every RI at a detectable level in the CT26 RNA-seq dataset (Dataset EV5). Peptides identified in the immunopeptidomics analysis were then matched to either this database or a standard proteomic database (containing all public domain mouse SwissProt protein entries). We identified 72 unique peptides derived from RI sequences (with a mean size of 9 residues; Fig. 4A–C; Dataset EV6). In parallel, we detected 5314 canonical peptides and 195 previously described lncRNA-derived peptides (Barczak et al, 2023) with comparable average signal intensity across all peptide categories (Appendix Fig. S6A). To validate each peptide sequence, we compared the mass spectrometry sequence profile with the synthetic peptide sequence, which allowed identity to be confirmed (Appendix Fig. S6B). Most importantly, the peptides exhibited quantitative differences between T1-44-treated and -untreated cells (Fig. 4A). All of the CT26-derived peptides were predicted to have high affinity for the murine MHC class I alleles H-2-Kd, Dd and Ld and exhibited a pattern of conserved residues required for efficient MHC class I binding (Fig. 4B; Appendix Fig. S6C), closely resembling the consensus for peptide sequences derived from protein coding genes (Reynisson et al, 2020).

We then examined the inclusion/exclusion ratio of the RIs in CT26 cells (Appendix Fig. S7A) and found that the presence of RI required PRMT5 in the majority of genes that we investigated (namely Eif4a2, Fam78b, Fut10, Ppp1r16a, Ptprg, Macrod, Cog3, Csnk1e, Scfd2). A decreased ratio was observed only in Adamts10 and Tgfbr1, and no change in Exoc6, Kat6b, Gm48619, and Dennd1a (Appendix Fig. S7A). Further, many of the murine RIs were encoded by E2F target genes (Appendix Fig. S7B). In a similar way to the human RIs, we identified small open-reading frames within each RI, which were subsequently matched to the peptides; for example, SGSPLVHGF to Cog3 RI, AGPSAALI to Csnk1e RI and YYIQQNAQL to Scfd2 RI (Appendix Fig. S7C). The murine RI transcripts were also able to associate with the translating polysome fraction of ribosomes (for example, the Cog3, Csnk1e, Dennd1a, Eif4a2, and Scftd RIs (Appendix Fig. S7D(ii,iv)), an observation consistent with a conventional ORF translation mechanism (Appendix Fig. S7D(iii,iv)). We therefore conclude that RI sequences that give rise to MHC class I-bound peptides reflect the presence of small ncORFs within the RI. Thus, a significant group of human and murine MHC class I-bound peptides is derived from translated ORFs within RIs.

### RI-derived MHC-bound peptides are immunogenic in mice and humans

To test whether RI-derived MHC-bound peptides are immunogenic, we designed and engineered a DNA cassette encoding a string of 29 murine peptides, which was cloned into ChAdOx1 (Fig. 4D; Appendix Fig. S8A), an established expression vector for the experimental immunisation of mice (Warimwe et al, 2013). To assess peptide immunogenicity, Balb/c mice were given a priming immunisation with the RI peptide vector, referred to as PepMInt (for Peptides from Murine Introns) vaccine. Nine days after priming, splenocytes were harvested and IFNγ production measured in an ELISpot assay against five RI-derived peptide pools (Fig. 4E,F; Appendix Figs. S8B and S9A). A remarkably high IFN-γ signal was evident after the priming immunisation, most likely reflecting an antigen-specific T lymphocyte response, as compared to mice immunised with a control ChAdOx1-GFP vector, where the IFN-γ signal was minimal (Fig. 4E,F; Appendix Fig. S8B). Moreover, using the same source of splenocytes and performing ELISpot on single peptides, we identified the most immunodominant peptides (Appendix Fig. S9) and further identified a sequence trend that was apparent in the immunogenic and not non-immunogenic peptides (Appendix Fig. S10A). These results indicate that murine RI-derived MHC class I-bound peptides are immunogenic and can stimulate an antigen-specific T lymphocyte response.

We progressed on to test whether the T lymphocyte activity generated by vaccination with PepMInt translated into a therapeutic benefit in tumour-bearing mice (Fig. 4G; Appendix Fig. S10B,C). Strikingly, a single vaccination with PepMInt delayed the growth of the CT26 tumours and increased survival compared to the control group vaccinated with the GFP vector (Fig. 4G). By day 17, we observed a 60% reduction in tumour growth (Fig. 4G), which coincided with a high level of IFN-γ-producing T lymphocytes in the PepMInt treatment group as indicated in the ELISpot assay (Appendix Fig. S10C). These results establish that the PepMInt vaccine stimulates a remarkably potent and therapeutically effective immune response, which hinders tumour growth and prolongs survival in the CT26 model. The induction of strong peptide-specific IFN-γ responses together with the therapeutic efficacy of PepMInt, which is derived from MHC class I-bound peptides, strongly supports a role for CD8⁺ T cells. However, definitive demonstration of a CD8 T-cell dependence in vivo would require further experiments.

We next evaluated combining PepMInt vaccination with PRMT5 inhibition (T1-44), which indicated that the combined treatment did not result in improved tumour control or an increase in ELISpot read-out compared to PepMInt alone, at least under these conditions (Appendix Fig. S10D–F). One possible explanation is that PRMT5 inhibition itself promotes the presentation of retained intron (RI)-derived peptides, thereby reshaping the tumour antigen landscape in a manner that could be similar to the effect of PepMInt vaccination. Each treatment could therefore induce overlapping effects that diminish the effect of the combined treatment (Appendix Fig. S10D–F).

### Human T lymphocytes from cancer patients recognise RI-derived peptides

**Figure 5:**
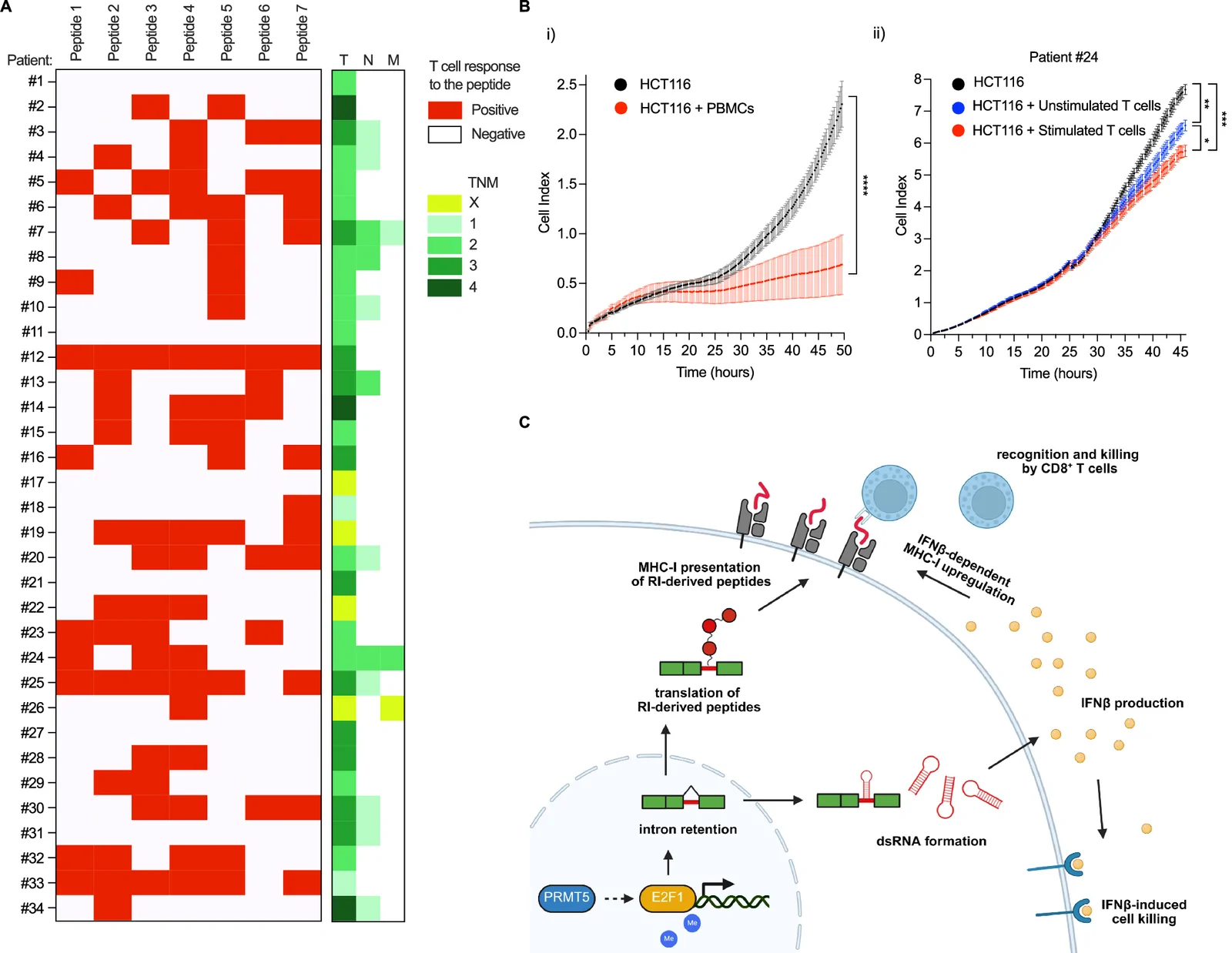
Human T lymphocytes recognise RI-derived peptides. (**A**) Heatmap representation of the results of the flow cytometry-based Cell Tracker Proliferation Assay performed on the blood collected from 34 Colon Cancer Patients. TNM status of the Patients has been displayed as well. Red colour represents a positive response to the peptide from CD8^+^ and/or CD4^+^ T cells. Patients’ TNM status (T—Tumour size; N—local metastasis to lymph node; M—distant metastasis) is also presented. (**B**) (i) Killing assay performed with HCT116 cells treated with human PBMCs from donors #20, #26 and #28 compared to untreated control. Data presented as a mean ± SEM; *n* = 3, each biological repeat is a separate donor. Significance calculated using *t* test, ****P value < 0.0001; HCT116 vs HCT116 + PBMC—*P* value = <0.0001; (ii) Killing assay performed with HCT116 cells treated with T cells enriched from PBMCs (Patient #24) that had been stimulated (red line) or unstimulated (blue line) with a pool of seven RI-derived peptides, compared to untreated control (black line). Results presented as a mean ± SEM calculated from the data of three sets of T cells isolated and expanded from one Patient. Significance calculated using one-way ANOVA; *P < 0.05, **P < 0.01, ***P < 0.001; HCT116 vs HCT116 + Unstimulated T cells —*P* value = 0.0083, HCT116 vs HCT116 + Stimulated T cells—*P* value = 0.0005, HCT116 + Stimulated T cells vs HCT116 + Unstimulated T cells—*P* value = 0.0388. (**C**) Model illustrating the interplay between adaptive and innate immune responses regulated by the PRMT5-E2F1 axis. PRMT5 through its control of E2F1 alters target gene specificity and influences intron retention. In the cytoplasm, RIs form double-stranded RNAs (dsRNAs) that trigger the production of IFN-β, upregulating the surface expression of MHC I molecules. Concurrently, the RIs provide an additional source of peptides that are translated and presented on MHC I complexes, leading to T-cell recognition and specific cell killing. Source data are available online for this figure.

We were interested to determine whether antigen-specific T cells are present in humans which recognise human RI-derived MHC-bound peptides. We used PBMCs (Peripheral Blood Mononuclear Cells) donated by 34 patients suffering from colorectal cancer (reflecting the pathological origin of HCT116 cells) and tested their response to some of the RI-derived peptides using an ex vivo T lymphocyte proliferation assay (Fig. 5A; Appendix Fig. S11A–C). We measured the proliferation of CD4^+^ and CD8^+^ lymphocytes stimulated by seven individual HLA-A02:01 peptides (Appendix Fig. S11A), by tracking the decay of Cell Trace Far Red dye (Lyons and Parish, 1994) (Appendix Fig. S11B). For each assay, we included PHA-L and DMSO treatments, which served as positive and negative controls.

Remarkably, we found that lymphocytes taken from 70% of the patients responded to at least two out of seven peptides tested, as reflected in T lymphocyte proliferation after the incubation period (Fig. 5A; Appendix Fig. S11C). It was notable however that the T-cell response was selective and patient-specific (for example, Patient 8 was highly responsive to Peptide 5 but unresponsive to Peptide 4, and Patient 20 showed a high response to Peptide 4 and low to Peptide 3). Patient #12 was the exception, as their T-cell response was detected against all the peptides.

We were interested to further establish whether patient-derived T lymphocytes, proliferating in response to the RI peptides, could kill HCT116 cells in vitro. To test this possibility, we expanded PBMC populations in which we had identified T lymphocyte activity against RI-derived peptides, added them to HCT116 cells and recorded the well coverage via bioimpedance (a measure of cell viability). Strikingly, we found that the PBMC populations harboured T lymphocytes which were able to kill HCT116 cells, leading to a fivefold decrease in cell confluency when compared with untreated control (Fig. 5B(i)). Moreover, when we re-stimulated enriched T lymphocytes with the RI-derived peptides (the total seven peptides), the extent of target cell killing was magnified and statistically significant in T lymphocyte responders (Fig. 5(ii); Appendix Fig. S11D). Further, no cell killing or effect of re-stimulation was observed in the peptide-unresponsive patient 31 (Appendix Fig. S11E), suggestive of an antigen-specific T lymphocyte response. These results demonstrate that T lymphocytes taken from some human patients with CRC are antigenically specific for the RI-derived peptides and, when co-cultured in vitro with the RI peptides, exhibit improved killing of target HCT116 cells, which naturally express the RI-derived peptides.

### Presence of RIs in human cancer

It was of interest to assess the abundance of RIs transcripts in human disease. We focused on CRC and measured the level of RIs in open-access data (http://www.oncosplicing.com/) (Zhang et al, 2022a). To assess any prognostic significance, we related RI expression to patient survival (Zhang et al, 2019). When distinct RIs (derived from CDK10, DDX55, and HDAC6) were analysed, in each case we found that elevated expression coincided with poor survival (Appendix Fig. S12A,B). We reason that these tumours could present RI-derived peptides bound to the MHC class I complex, and thus may be responsive to a cancer vaccine with RI-derived peptides, as we have exemplified here with the mouse tumour model.

## Discussion

PRMT5 has gained prominence as an interesting cancer target, and numerous clinical trials are underway assessing the therapeutic benefit of small-molecule inhibitors (de Cordoba Sanchez et al, 2026). Its methyltransferase activity is established to regulate a variety of pathways involved in oncogenesis, such as the pRb-E2F pathway, whose deregulation is regarded as a hallmark of cancer. In addition to its pathway-specific effects, PRMT5 is known to influence gene expression and RNA splicing control (Bate-Eya et al, 2025; Bezzi et al, 2013; Deng et al, 2010; Gillespie et al, 2025). In RNA splicing, PRMT5 methylates a variety of SF that allow proper splicing to occur before the mature RNA is translated. Inactivating PRMT5 leads to deregulated gene expression and splicing (Barczak et al, 2020; Roworth et al, 2019).

The results described here establish that PRMT5 has a significant effect on controlling the immune response against tumour cells. Through its influence on RNA splicing, PRMT5 can regulate the level and processing of RNA. One consequence of PRMT5 inhibition is the altered level of RIs, reflecting the role of PRMT5 in regulating splicing events. We have found that RIs, on the one hand, form dsRNA structures that activate the innate IFN response in cancer cells. Double-stranded RNAs generated from endogenous RNA sequences and structured transcripts are well established to activate type I interferon signalling (Kato et al, 2006; Zheng et al, 2024) through cytosolic RNA sensors such as PKR, RIG-I and MDA5, providing a plausible mechanistic basis for the immune activation observed in our system (Chen and Hur, 2022; Der and Lau, 1995). Although the IFN signalling can be induced by other species of RNA, such as single-stranded RNA (Heil et al, 2004), circular RNAs (Chen et al, 2017) or RNA:DNA hybrids (Mankan et al, 2014), dsRNA is considered a predominant trigger. RIs also perform a complementary role by driving an adaptive immune response, reflecting the translation of small ncORFs present in the RIs, and peptides derived from them, which assemble with MHC class I to foster a T-cell response.

Notably, the majority of RI-derived ncORFs identified in our study initiate from non-AUG codons. Translation from such non-AUG start sites is consistent with established alternative initiation mechanisms, which are increasingly recognised as contributors to the generation of cryptic antigens (Deng et al, 2024; Kearse and Wilusz, 2017). Consistent with active translational engagement, our polysome profiling RNA-seq revealed substantial read coverage across peptide-coding intronic regions following PRMT5 inhibition. The enrichment of the reads within translating fractions supports the notion that intron-containing transcripts are accessible to ribosomes and capable of generating antigenic peptides.

These results thus point to a role for PRMT5 in regulating the immune response against cancer cells by virtue of its qualitative and quantitative impact on RIs. Consistent with this concept, therapeutic interventions such as radiation, chemotherapy, cytokine exposure and targeted agents (including PRMT5 inhibition (Barczak et al, 2023) are increasingly recognised as capable of remodelling the tumour immunopeptidome, by altering both the quality and the repertoire of presented peptides (Sagie et al, 2025; Tailor et al, 2022; Yang et al, 2023), although the contribution of RI-derived peptides as yet is to be determined.

It is noteworthy that antigen-specific T cells against RI-derived peptides were detected in cancer patients. This raises the interesting possibility that a vaccine, designed to express RI-derived antigens, could be used to enhance the adaptive immune response against tumours with an aberrant splicing programme that produces RIs, which is a common feature of many cancers (Dvinge and Bradley, 2015). Although the RI-reactive T-cell responses were heterogeneous, this variability is consistent with the well-recognised diversity of HLA haplotypes within the human population (Oseni et al, 2025). The detection of peptide-specific T-cell responses against RI-derived antigens in CRC patients nevertheless supports the immunogenic potential of this antigen source. Inter-patient variability suggests that RI-based vaccine strategies may benefit from stratification approaches. For example, enrichment for patients expressing common alleles such as HLA-A*02 could facilitate more uniform immune targeting in early clinical studies. In addition, functional stratification based on ex vivo assessment of RI-specific T-cell responses may help identify patients most likely to benefit from vaccination. Given the heterogeneity in peptide immunogenicity, a personalised strategy selecting RI-derived peptides according to individual HLA genotype and measured peptide responsiveness may further enhance therapeutic efficacy.

Although we identified T-cell responses against RI-derived peptides in CRC patients, higher RI transcript expression was unexpectedly associated with poorer survival. While future studies may address this issue, it is certainly possible that high RIs in tumours do not translate to immunogenic antigens, possibly reflecting the highly immunosuppressive tumour microenvironment in aggressive tumours (Calcinotto et al, 2012; Freeman et al, 2000).

Nevertheless, in tumour-bearing mice, we showed that vaccination with PepMInt (expressing a string of murine RI-encoded peptides) created a powerful antigen-specific T lymphocyte response which led to a consequential delay in tumour progression and enhanced survival. Based on these results, we might anticipate that a cancer vaccine, expressing RI-encoded peptides identified in human tumour cells, may provide clinical benefit in human cancer. Moreover, given the increased dsRNA upon inhibition of PRMT5 and the active innate IFN response, we reason that other agents that influence either the innate or adaptive immune response may provide effective combinations with a targeted vaccine as described here.

It has been established that the levels of RIs are generally lower in normal cells and increased in tumour cells (Dvinge and Bradley, 2015; Shah et al, 2022), which may relate to the role of E2F1 on RIs (Fig. 1) and the frequent deregulation of the pRb-E2F pathway in cancer (Barczak et al, 2020; Roworth et al, 2019). Normal cells can however suffer from abnormal RNA splicing upon exposure to different types of stress, such as hypoxia and DNA damage (Dutertre et al, 2011; Memon et al, 2016), and RIs have been shown to increase in ageing and senescent cells (Pabis et al, 2024; Yao et al, 2020). It is feasible, we speculate, that the appearance of RIs under conditions that are detrimental to cell survival leads to increased presentation of RI-derived peptide antigens by the MHC, which in turn prompts the immune system to target and eliminate the damaged cells. This could represent a powerful mechanism for immune surveillance, where immune destruction of unhealthy or damaged cells is dependent on antigen presentation triggered by aberrant RNA splicing events (Fig. 5C).

RIs have previously been identified as a source of cancer antigens, although their clinical relevance, for example, impact on disease progression and prognosis, remains to be determined (Dong et al, 2022; Dong et al, 2021; Dvinge and Bradley, 2015; Rosenberg-Mogilevsky et al, 2025; Smart et al, 2018; Yong et al, 2025). In other studies, AS events were identified as a source of tumour-specific antigens (Huang et al, 2024; Kwok et al, 2025; Pan et al, 2023; Shen et al, 2019). Here, we present a very significant advance on our current knowledge by outlining a strategy to target malignant disease, based on the status of PRMT5 and E2F1, and the role of RI-derived antigens in driving a tumour-specific immune response.

In conclusion, our results have described the interplay between the innate and adaptive immune response, orchestrated by PRMT5 and E2F1, and dependent on RIs derived from deregulated RNA splicing. Our findings significantly advance the understanding of PRMT5 and E2F1 biology and highlight a new source of human T cell antigens that may be important immunological targets when deployed in cancer vaccines. Most importantly, because intron retention can generate novel antigens, our results also define a potential immune surveillance mechanism that identifies and eliminates damaged cells that exhibit a deregulated RNA splicing programme.

## Methods

**Table:** Reagents and tools table

Reagent/resource	Reference or source	Identifier or catalogue number
**Experimental models**		
HCT116 cell line	ATCC	CCL-247
CT26 cell line	ATCC	CRL-2638
PANC1 cell line	ATCC	CRL-1469
MCF7 cell line	ATCC	HTB-22
SHEP2 cell line	Bate-Eya LT et al, 2025. https://doi.org/10.1002/1878-0261.13702	N/A
GIMEN cell line	Bate-Eya LT et al, 2025. https://doi.org/10.1002/1878-0261.13702	N/A
BALB/cAnNCrl	CRL	028
W6/32	ATCC	HB-95
34-1-2S	ATCC	HB-79
PBMCs collected from Colon Cancer Patients	This paper	N/A
**Recombinant DNA**		
pcDNA_3.1 + _RI_DDX55	This study/synthetized in Genscript	N/A
pcDNA_3.1 + _EXON_DDX55	This study/synthetized in Genscript	N/A
pcDNA_3.1 + _RI_CDK10	This study/ synthetized in Genscript	N/A
pcDNA_3.1 + _EXON_CDK10	This study/synthetized in Genscript	N/A
pcDNA_3.1 + _RI_HDAC6	This study/synthetized in Genscript	N/A
pcDNA_3.1 + _EXON_HDAC6	This study/synthetized in Genscript	N/A
pcDNA_3.1 + _RI_A1BG	This study/synthetized in Genscript	N/A
pcDNA_3.1 + _EXON_A1BG	This study/synthetized in Genscript	N/A
**Antibodies**		
FITC Mouse Anti-Human HLA-A2	BD Pharmingen	551285
FITC Mouse Anti-Human CD3	BD Pharmingen	3248390
PE Mouse Anti-Human CD4	BD Pharmingen	566679
PE Mouse Anti-Human CD8	BD Pharmingen	555367
FITC Mouse Anti-Human HLA-ABC	BD Pharmingen	560965
J2 dsRNA antibody	Nordic Biosite	246-10010500
FITC-conjugated IFN-β antibody	Invitrogen	BMS1044F1
Alexa Fluor 488 conjugated secondary antibody	Invitrogen	A-11059
IFNAR	Medchemexpress	HY-P99168
β-actin	Sigma-Aldrich	A2228
E2F1	Cell Signaling Technology	3742S
SDMe	Cell Signaling Technology	13222S
IFN-β antibody	Thermo Fisher Scientific	PA5-20390
IFNγ mAb clone AN18	MabTech	3321-3-1000
biotin conjugated anti-IFNγ	MabTech	3321-6-100
Cell Tracker Far Red	ThermoFisher Scientific	C34572
Secondary HRP-conjugated antibody mouse		
Secondary HRP-conjugated antibody rabbit		
**Oligonucleotides and other sequence-based reagents**		
exonic_forward_Fam78b	Merck	AGGCATGCAACCAGATGGAG
exonic_forward_Eif4a2	Merck	ATGTTGAGCGAGAGGAGTGG
exonic_forward_Fut10	Merck	CTGGGGACTTGTTTTGCTGC
exonic_forward_Ptprg	Merck	CATTATGTGGTGTGCTTCCCG
exonic_forward_Adamts10	Merck	TGCCTGCAACACAGAACCAT
exonic_forward_Gm48619	Merck	TCGACAGGATGAGTACGAG
exonic_forward_Tgfbr1	Merck	TTGCTGCAATCAGGACCACT
exonic_forward_Macrod1	Merck	GGTGGACGGCTGCATTCAT
exonic_forward_Exoc6	Merck	TCAGTCTTTTGAGCTGCCCAT
exonic_forward_Ppp1r16a	Merck	AGGTTCCAGGTGTGTGTGAC
exonic_forward_Kat6b	Merck	TACCCCAGTGCTTTTCCGTC
exonic_forward_Dennd1a	Merck	AGGACATCTGTCTCAAGCCC
exonic_forward_Cog3	Merck	GCCTGCATACAGTCCTTGCTA
exonic_forward_Csnk1e	Merck	CAGGGTTTCTCCTACGACTACG
exonic_forward_Scfd2	Merck	CAGAACGTGATTGCACCAGG
exonic_forward_ARID1B	Merck	GCCCCCGGAAAACCTAACC
exonic_forward_SCHIP1	Merck	GGTGCCACCTATGGATTGGG
exonic_forward_FAM188B	Merck	GAAAGAGCGTTCAAACGGCA
exonic_forward_ZNF592	Merck	CACACCCTTGTTTTGAGA
exonic_forward_ATAD5	Merck	CCTCAGACTGCCAGTGAACTT
exonic_forward_RSPRY1	Merck	TCTTTGCCGTTCTCTCGGAC
exonic_forward_CDKAL1	Merck	ACTGTGTGGCTGACTTCAAA
exonic_forward_FNDC3B	Merck	AGAGCAGAGGATGGAACACT
exonic_forward_KLHL29	Merck	ATCCTGGGGACGAATCCTGA
exonic_forward_NR2C2	Merck	GGCGCCAAATCCTGAGGTAA
exonic_forward_MIB1	Merck	CTTACGACCTCCGCATCCTG
exonic_forward_DDX55	Merck	CCCCGAGTTCAGCCAGATTC
exonic_forward_CDK10	Merck	GGTGGTCACTCTCTGGTACCGA
exonic_forward_HDAC6	Merck	GATGCGGGATGCTGACTACA
exonic_forward_A1BG	Merck	CTACCGGACCGATGGGGAA
exonic_forward_SPIN2B	Merck	GGATGGAAGGAAGGAGATGAGC
exonic_reverse_Fam78b	Merck	CCTTCCCTCAAGTCAGGCAG
exonic_reverse_Eif4a2	Merck	ACTGCTTGTGTGATAGTCAAAGT
exonic_reverse_Fut10	Merck	ACAGCTGGAGTCCTCAGACA
exonic_reverse_Ptprg	Merck	GAGCTGTCCTGTCTGCTCTC
exonic_reverse_Adamts10	Merck	ACACCAGCGTCACAGCTAC
exonic_reverse_Gm48619	Merck	TCCCAGTCTCCTCTTTCTTC
exonic_reverse_Tgfbr1	Merck	GCCAGCTGACTGCTTTTCTG
exonic_reverse_Macrod1	Merck	GGTCTCGCAGTTCTGTAGGG
exonic_reverse_Exoc6	Merck	TTCTTGTGTGCGTTTGGCTG
exonic_reverse_Ppp1r16a	Merck	AGGCAGCTAGGTAGACAGGAG
exonic_reverse_Kat6b	Merck	ATTGGAATGGGATCGGCACG
exonic_reverse_Dennd1a	Merck	CATCACCTTCTGCGCTGTCT
exonic_reverse_Cog3	Merck	GTTGTCCATCAATCTGAGTCTTGT
exonic_reverse_Csnk1e	Merck	GTTCGTGCTCCCGTCTTTCC
exonic_reverse_Scfd2	Merck	ACTGCCTCTTTGTGCTTGGA
exonic_reverse_ARID1B	Merck	CGTGGGGAGGTCATCAATGG
exonic_reverse_SCHIP1	Merck	TTTTCTGTGCCTCCTGCTCC
exonic_reverse_FAM188B	Merck	ACCATCCACCTTGTCAGACG
exonic_reverse_ZNF592	Merck	CGTCTTCCTTCTGTCTTC
exonic_reverse_ATAD5	Merck	GCCTTTCTTCCAATTCAGCTCT
exonic_reverse_RSPRY1	Merck	ATCCAATGGCAGTTCGGAGG
exonic_reverse_CDKAL1	Merck	TTTCTCCAGGAAAACCACAGA
exonic_reverse_FNDC3B	Merck	ACCTGGAGGCACATGAATGG
exonic_reverse_KLHL29	Merck	GTGAGCTAAGCAGAGACCTAC
exonic_reverse_NR2C2	Merck	TTATCTGGATGCGTGGGGAG
exonic_reverse_MIB1	Merck	CAATGATTGGTTGCTGGCGG
exonic_reverse_DDX55	Merck	CCTTGTTGCTTAAACCTCTCAACA
exonic_reverse_CDK10	Merck	ACATGTCGATGCTGGTGGTCT
exonic_reverse_HDAC6	Merck	GAGGCTGGAACTCGAGGG
exonic_reverse_A1BG	Merck	TGCAGCGAGCTCCTCAATG
exonic_reverse_SPIN2B	Merck	AATCATCTAGAAGCTGATCCAGAAC
intronic_forward_Fam78b	Merck	TCAACACCTACAGCGACCTG
intronic_forward_Eif4a2	Merck	TGTTGAGCGAGAGGTAATTTTGT
intronic_forward_ Fut10	Merck	GGACTTGTTTTGCTGCCCTG
intronic_forward_Ptprg	Merck	CATTATGTGGTGTGCTTCCCG
intronic_forward_Adamts10	Merck	GCAACACAGAACCATGTCCAC
intronic_forward_Gm48619	Merck	TCCCCACCAAAGAGGTTCAG
intronic_forward_Tgfbr1	Merck	TGCAATCAGGACCACTGCAATA
intronic_forward_Macrod1	Merck	GGGGTGAGTACTAGACCTTCCT
intronic_forward_Exoc6	Merck	TTTGAGCTGCCCATTGAAGC
intronic_forward_Ppp1r16a	Merck	GTTCAGGGAGCGGCCAG
intronic_forward_Kat6b	Merck	AAAGACCAGGTGAGTGAGCC
intronic_forward_Dennd1a	Merck	GGACATCTGTCTCAAGCCCT
intronic_forward_Cog3	Merck	GCCTGCATACAGTCCTTGCT
intronic_forward_Csnk1e	Merck	GGCAGGGTTTCTCCTACGAC
intronic_forward_Scfd2	Merck	CAGGCTGTGGAGGAAAACCA
intronic_forward_ARID1B	Merck	GCAAGAAAGACCATCAAGTTTACCA
intronic_forward_SCHIP1	Merck	CTGGAGAAGCATCTGGCCG
intronic_forward_FAM188B	Merck	GAGAGGCTGGAAAGAGCGTT
intronic_forward_ZNF592	Merck	CGCCACACCCTTGTTTTGAG
intronic_forward_ATAD5	Merck	ACCTCAGACTGCCAGTGAAC
intronic_forward_RSPRY1	Merck	CGTTCTCTCGGACCTGTCAC
intronic_forward_CDKAL1	Merck	GTAGTGGATTTTCTGAAAGAGAAGT
intronic_forward_FNDC3B	Merck	CAGTGCATTCAAGGTAAGGACA
intronic_forward_KLHL29	Merck	CGCAAGGTAGGTCTCTGCTT
intronic_forward_NRC2C	Merck	CCTGAGGTAAAATTGAAAGAGGG
intronic_forward_MIB1	Merck	GCTTACGACCTCCGCATCC
intronic_forward_DDX55	Merck	TCAAAGGCTGTTTTCTTGTTGTAG
intronic_forward_CDK10	Merck	GGAGAGGAGCCGGCTGTATT
intronic_forward_HDAC6	Merck	AGCCTCATGGAGTCTGTTCC
intronic_forward_A1BG	Merck	GCAGCTACCGGACCGATG
intronic_forward_SPIN2B	Merck	ATGGAAGGAAGGAGATGAGCCC
exonic_reverse_Fam78b	Merck	TGGAGAAAGGGGCCAGGA
exonic_reverse_Eif4a2	Merck	AGTTCCTGCAATTTTAGCACAGT
exonic_reverse_ Fut10	Merck	TGTCTCGTCTTACACTCCGC
exonic_reverse_Ptprg	Merck	GACAACTCCGCATACCCCG
exonic_reverse_Adamts10	Merck	GCAGAGAACACTCTCACCCA
exonic_reverse_Gm48619	Merck	GAGAGCCTAATCCCAAACCC
exonic_reverse_Tgfbr1	Merck	GGGAAGGGTGCTAATGCAAC
exonic_reverse_Macrod1	Merck	GAAACAGGAGACCCATACCCC
exonic_reverse_Exoc6	Merck	AGACGGAAAAGAGAAATGAAGGT
exonic_reverse_Ppp1r16a	Merck	CCAGATAGACCCGACCCTCA
exonic_reverse_Kat6b	Merck	ACGCCTAAGTGGAAGCGAAA
exonic_reverse_Dennd1a	Merck	TCATTGGGTTCCTGGGGAGA
exonic_reverse_Cog3	Merck	TCACCTAACGCTTCATCAAACC
exonic_reverse_Csnk1e	Merck	CTGTCCAGTCCCCCAAGC
exonic_reverse_Scfd2	Merck	GCCTCATGTCTTACTTGGATGG
exonic_reverse_ARID1B	Merck	ACAAACAAAAACAACAGCTCTTTC
exonic_reverse_SCHIP1	Merck	GCCAACTTACGGAGGTGGA
exonic_reverse_FAM188B	Merck	GAAAAGCCGTCACCTTTGCAT
exonic_reverse_ZNF592	Merck	GCTTTTCAAGGCCAGTTCAGG
exonic_reverse_ATAD5	Merck	CAATTCCTTACACATTTTACCAACC
exonic_reverse_RSPRY1	Merck	GGCTGTGGCACTGACTTTCT
exonic_reverse_CDKAL1	Merck	TGCCACAAGCTTTTGAAAGAAA
exonic_reverse_FNDC3B	Merck	TGCAGACACACACAGAAAAACAT
exonic_reverse_KLHL29	Merck	AGAGAGCTGGCTATGCTCAGG
exonic_reverse_NRC2C	Merck	GCGGCGCCTCCATGT
exonic_reverse_MIB1	Merck	CGCAGCTCGCCCCTTC
exonic_reverse_DDX55	Merck	TCTCAACATCTTCTCCAGGATTC
exonic_reverse_CDK10	Merck	ACATGTCGATGCTGGTGGTCT
exonic_reverse_HDAC6	Merck	CCTTGCAGGGCATCAAATCC
exonic_reverse_A1BG	Merck	GATGGTTCCCACAGTCACCG
exonic_reverse_SPIN2B	Merck	GGGATTTATAGGCACCTGATCCA
peptide_flanking_forward_Fam78b	Merck	CAGCTTGAATCCAGTGATCTGGC
peptide_flanking_forward_Eif4a2	Merck	TCATGATCTTTGTGGGTAGCA
peptide_flanking_forward_Ptprg	Merck	GAGAGAGACAGAAGCCAGTCAC
peptide_flanking_forward_Dennd1a	Merck	CAGGGGAAGCCTCTAGGAGT
peptide_flanking_forward_Cog3	Merck	TCCCTAGAGAAGCTCTGCCTT
peptide_flanking_forward_Csnk1e	Merck	TGGGCTCCAGTCTCTCTTGT
peptide_flanking_reverse_Fam78b	Merck	TTATGAGGGTACAGAGCCCCA
peptide_flanking_reverse_Eif4a2	Merck	TTCTATTGCTGCAGCCCTCT
peptide_flanking_reverse_Ptprg	Merck	TGTATGGAGGGAGGCAGTCTT
peptide_flanking_reverse_Dennd1a	Merck	CTATTACGCCAGGGTGAGGC
peptide_flanking_reverse_Cog3	Merck	TGTCCCATGCTCCTTTAATCCC
peptide_flanking_reverse_Csnk1e	Merck	CAGACAGCTATCAGGGCTGC
GAPDH forward	Merck	CATCTTCCAGGAGCGAGATCC
IVT PCR primer forward	Merck	AGAACCCACTGCTTACTGGCT
GAPDH reverse	Merck	ATGACGAACATGGGGGCATC
IVT PCR primer reverse	Merck	CCCTCTAGACTCGAGCGGC
PKR forward	Merck	TGCATGGGCCAGAAGGATTT
PKR reverse	Merck	GCGGCCAATTGTTTTGCTTC
Non-targeting siRNA	Merck	AGCUGACCCUGAAGUUCUU
siPKR	Merck	EHU127861
pcDNA_3.1 + _RI_DDX55	Genscript	CGGATTTCGGCCTGGCCCGGGCCTATGGTGTCCCAGTAAAGCCAATGACCCCCAAGGTGGTCACTCTCTGGTAAGTCCTTCTGAAGCATGGTGGCCCCTGGGGACCAGGCCTGTCTGGTGGAGGTCTCCTTGGGGATGTCAGGCCGAAGCTGCAACTGGCCTTGGGAATGTTAAGCTACAGGGTCTGTGCACACTCAGAAACGTTGGGTCTAGGACAGCCTCCAGGACACAGCAGGGCTGTGCCACAAAATGGTGAGAGACGGGCTCAGTGGCGTCAAGGGCCTGCCTAGATCAGACACACCTCGCCTTGAGAGCTGGAAACACCGTGGCCGTGAAACCATCTCTGGAGGGAAGGCTGCTTCACGGACTGAAGGACAGGCAGCTTGCATGCTTTCCCTGGTTCTTCCTCCTGGATACTTTTAACCAAACAGGGTCATGCTTAGCCAGGGCTGAATATCAGAGTTGGTCAGAATCAGGTCAGGACCCCAGTGTCGTGTACTCCCCATCTGAGAACGGGTTGGTAGTGGCTGGTGTGGCTGTGTGCCGAGGGTCCGTGGTCCCTGCGACTCTCAGGACACCGCTGCACGTGACAGCTCTCGCCCCCGGCAACCATTGCTGTGTGTGCCACCTGGATCTGCAGGGCCTGGGGCAGAGGGTGGTCCGAGTTGGGACAGGTTTCCAGGCCACCACAGGCTCTCGGGGAGGCTGTGTCCTGTGGAGGCCTGGGCTGGGGGAGAGGAGCCGGCTGTATTGAGGTGGGTGCTTCTGTGTGTAGGTACCGAGCCCCTGAACTGCTGTTGGGAACCACCACGCAGACCACCAGCATCGACATGTG
pcDNA_3.1 + _EXON_DDX55	Genscript	CGGATTTCGGCCTGGCCCGGGCCTATGGTGTCCCAGTAAAGCCAATGACCCCCAAGGTGGTCACTCTCTGGTACCGAGCCCCTGAACTGCTGTTGGGAACCACCACGCAGACCACCAGCATCGACATGTG
pcDNA_3.1 + _RI_CDK10	Genscript	GTTGGAGCCATAATCATCACCCCCACTCGAGAGCTGGCCATTCAAATAGACGAGGTCCTGTCGCATTTCACGAAGCACTTCCCCGAGTTCAGGTGAATTGGATGCAGTGTCCCTGTTAGTCATGGGCTGTTTTGTCGAACTTAATCAAAGGCTGTTTTCTTGTTGTAGCCAGATTCTTTGGATCGGAGGCAGGAATCCTGGAGAAGATGTTGAGAGGTTTAAGCAACAAGG
pcDNA_3.1 + _EXON_CDK10	Genscript	GTTGGAGCCATAATCATCACCCCCACTCGAGAGCTGGCCATTCAAATAGACGAGGTCCTGTCGCATTTCACGAAGCACTTCCCCGAGTTCAGCCAGATTCTTTGGATCGGAGGCAGGAATCCTGGAGAAGATGTTGAGAGGTTTAAGCAACAAGG
pcDNA_3.1 + _RI_HDAC6	Genscript	GTGGGGATGCGGGATGCTGACTACATTGCTGCTTTCCTGCACGTCCTGCTGCCAGTCGCCCTCGAGGTCCTGGGGATCTGGGGTGTGTTGGGAGGAGGAGTGGCCTTGAAGGTTAGGCTGTGAGGGTAGGTAGCCTCATGGAGTCTGTTCCCCCTTCAGTTCCAGCCTCAGCTGGTCCTGGTGGCTGCTGGATTTGATGCCCTGCAAGGGGACCCCAAG
pcDNA_3.1 + _EXON_HDAC6	Genscript	GTGGGGATGCGGGATGCTGACTACATTGCTGCTTTCCTGCACGTCCTGCTGCCAGTCGCCCTCGAGTTCCAGCCTCAGCTGGTCCTGGTGGCTGCTGGATTTGATGCCCTGCAAGGGGACCCCAAG
pcDNA_3.1 + _RI_A1BG	Genscript	AGTCCTTGCCTGCTCCCTGGCTCTCGATGGCGCCAGTGTCCTGGATCACCCCCGGCCTGAAAACAACAGCAGTGTGCCGAGGTGTGCTGCGGGGTGTGACTTTTCTGCTGAGGCGGGAGGGCGACCATGAGTTTCTGGAGGTGCCTGAGGCCCAGGAGGATGTGGAGGCCACCTTTCCAGTCCATCAGCCTGGCAACTACAGCTGCAGCTACCGGACCGATGGGGAAGGCGCCCTCTCTGAGCCCAGCGCTACTGTGACCATTGAGGAGCTCGCTGCACCACCACCGCCTGTGCTGATGCACCATGGAGAGTCCTCCCAGGTCCTGCACCCTGGCAACAAGGTGACCCTCACCTGCGTGGCTCCCCTGAGTGGAGTGGACTTCCAGCTACGGCGCGGGGAGAAAGAGCTGCTGGTACCCAGGAGCAGCACCAGCCCAGATCGCATCTTCTTTCACCTGAACGCGGTGGCCCTGGGGGATGGAGGTCACTACACCTGCCGCTACCGGCTGCATGACAACCAAAACGGCTGGTCCGGGGACAGCGCGCCGGTCGAGCTGATTCTGAGCGATG
pcDNA_3.1 + _EXON_A1BG	Genscript	AGTCCTTGCCTGCTCCCTGGCTCTCGATGGCGCCAGTGTCCTGGATCACCCCCGGCCTGAAAACAACAGCAGTGTGCCGAGGTGTGCTGCGGGGTGTGACTTTTCTGCTGAGGCGGGAGGGCGACCATGAGTTTCTGGAGGTGCCTGAGGCCCAGGAGGATGTGGAGGCCACCTTTCCAGTCCATCAGCCTGGCAACTACAGCTGCAGCTACCGGACCGATGGGGAAGGCGCCCTCTCTGAGCCCAGCGCTACTGTGACCATTGAGGAGCTCGGTGACTGTGGGAACCATCTCTGGGGGCTGCTGGGGGAGGAGGATACAGGGGGCATGACCACATTCATCCTGACCTCCATGCTGAATGTCCTTGCCCAGGACCAGGTCCAGGAAGCTGGCCACACGGTTGCCTGGGTCTGATGGTTGCAAAGGAGTTTGGGCAGAGGATGCCCAAAGAATGGAAGGGTGAGGCCGGGTGCGGTGGCTCACGCCTATAATCCCAGCACTTTGGGAGGCTGAGGCCAGCGGATCATGAGGTCAAGAGATCGAGACCAGTCTACCCAACATGACGAAACCCCGTCTCTACTAAAAATACAAAAATTAGCCAGGTGTGGTGGCAGGTGCCTGTAGTACCAGCTACTCAGGAGGCTGAGGCAGGAGAATCCCTTGAACCTGGGAGGCAGAGCTTGCAGTGAGCTGAGTGGCGCCACTGCACTCCAGCCTGGGCAACAAGAGCGAAACTCCGTTTCAAAAAAAAAAAAAAAAAAGAATGGAAGGGTGTTCCTGGGATTGCCACCCGGAGGGCAGAGGTCTGGGGACTGGGCAGAGGGCAGCTCCAGGGATGGGGCAGCCTAGTAATGCTGCCTGCATTGCAGCTGCACCACCACCGCCTGTGCTGATGCACCATGGAGAGTCCTCCCAGGTCCTGCACCCTGGCAACAAGGTGACCCTCACCTGCGTGGCTCCCCTGAGTGGAGTGGACTTCCAGCTACGGCGCGGGGAGAAAGAGCTGCTGGTACCCAGGAGCAGCACCAGCCCAGATCGCATCTTCTTTCACCTGAACGCGGTGGCCCTGGGGGATGGAGGTCACTACACCTGCCGCTACCGGCTGCATGACAACCAAAACGGCTGGTCCGGGGACAGCGCGCCGGTCGAGCTGATTCTGAGCGATG
**Chemicals, enzymes, and other reagents**		
DMEM	Merck	D6429
FBS	Labtech	FB-1345/500
penicillin/streptomycin	Gibco	15140122
trypsin-EDTA	Gibco	25200056
RPMI 1640	Sigma-Aldrich	SLM-240
L-glutamine	Gibco	25030081
Sodium pyruvate	Gibco	11360070
Direct-zol RNA MiniPrep kit	Zymo Research	R2052
TRIzol	Thermo Fisher Scientific	15596018
oligo (dT)20	Invitrogen	18418020
SuperScript III Reverse Transcriptase	Invitrogen	18080044
Brilliant III SYBR Green qPCR Master Mix	Stratagene	600883
Cytofix/Cytoperm kit	BD Biosciences	554714
DMSO	Sigma-Aldrich	D2438
PBS	Merck	D8537
IFN-β	R&D Systems	8499-IF-010
RIPA buffer	Merck	20-188
Bradford reagent	Bio-Rad	5000205
SDS	MP Biomedicals	102918
Glycine	Thermo Scientific	120070050
protease inhibitor cocktail	Merck	11836170001
Acrylamide	Serva	10688.01
Milk powder	Merck	1153630500
TEMED	Merck	T9281
Protein Standard	NEB	P7719
APS	Merck	A3678
Transfer kit for western blot	Bio-Rad	1704158
Clarity Western ECL Substrate	Bio-Rad	1705061
Formaldehyde	Merck	F8775
RNase III	Invitrogen	AM2290
BSA	Merck	05470
Tween20	Merck	P1379
Tween80	Merck	P1754
Fluoroshield mounting medium	Merck	F6182
DAPI	Invitrogen	62248
Histochoice	Sigma-Aldrich	H2779
Tris	VWR	443866 G
EDTA	Thermo Scientific	D/0650/53
Methanol	Merck	M1770
H2O2	Merck	H1009
Mouse-on-Mouse Polymer IHC Kit	Abcam	ab269452
Vectastatin ABC-HRP Kit	Vector Laboratories	PK-4001
DAB solution	Vector Laboratories	SK-4100
Haematoxylin	Sigma-Aldrich	SLCL4496
GeneJuice	Novagen	70967
Opti-MEM	Gibco	31985070
Oligofectamine transfection reagent	Invitrogen	12252011
ApaI	Thermo Scientific	FD1414
BclI	Thermo Scientific	FD0724
Phusion™ High-Fidelity DNA Polymerase	Thermo Scientific	F-530XL
Agarose	MP Biomedicals	193983
QIAquick Gel Extraction Kit	Qiagen	28704
T7-Scribe™ Standard RNA IVT Kit	Cellscript	C-AS3107
ScriptCap™ m7G Capping System	Cellscript	C-SCCE0625
ScriptCap™ 2’-O-Methyltransferase Kit	Cellscript	C-SCMT0625
Borax buffer	Sigma-Aldrich	HT1002
GelRed Nucleic Acid Stain	Merck	SCT123
ssRNA Ladder	NEB	N0362S
RNA Loading Dye	NEB	B0363A
Protein A agarose beads	Rockland	PA50-00-0025
Salmon sperm DNA	Invitrogen	15632011
NaCl	Merck	S9888
Igepal	MP Biomedicals	198596
Sodium deoxycholate	Merck	302-95-4
RNaseOUT	Invitrogen	10777019
GlycoBlue™ Coprecipitant	Invitrogen	AM9516
Random Hexamers	Invitrogen	N8080127
Sepharose-protein A beads	Expedeon/Abcam	ab270308
Boric acid	Merck	1.00165.0100
KCl	Merck	1.04936.0250
dimethyl pimelimidate	Merck	D8388
Citric acid monohydrate	Merck	1.00244.0500
Acetic acid	Merck	695092
Trifluoroacetic acid	Merck	302031
Acetonitrile	Merck	34851
Proteinase K	Thermo Scientific	EO0491
MgCl2	Merck	M8266
Cycloheximide	Merck	C7698
Triton X-100	Merck	X100
RNase inhibitor	Invitrogen	N8080119
Sucrose	Merck	S0389
Non-essential amino acids	Merck	M7145
ACK lysis buffer	Lonza	BP10-548E
PHA-L	Roche	11249738001
BCP/NBT substrate	Merck	B1911
Ficoll-Paque Plus	Merck	GE17-1440
human blood group type AB serum	Sigma	H3522
MojoSort™ Human CD3 T Cell Isolation Kit	Biolegend	480021
ImmunoCult Human CD3/CD28 T Cell Activator	Stemcell Technologies UK Ltd	10971
IL-2	Biolegend	589102
**Software**		
VAST software version 2.5.1	https://github.com/vastgroup/vast-tools	N/A
Trinity software package version 2.15.1	https://github.com/trinityrnaseq/trinityrnaseq	N/A
kallisto software version 0.44.0	https://github.com/pachterlab/kallisto	N/A
rMATS program version 4.0.2	https://rnaseq-mats.sourceforge.io/rmats4.0.2/index.html	N/A
STAR package version 020201	https://github.com/alexdobin/STAR	N/A
R Studio/Posit	https://posit.co/download/rstudio-desktop	N/A
ViennaRNA package version 2.4.18	https://github.com/ViennaRNA/ViennaRNA	N/A
BD Accuri C6 Plus software	BD	N/A
ImageJ software	National Institutes of Health	N/A
seqkit	https://anaconda.org/channels/bioconda/packages/seqkit/overview	N/A
Peaks v8.5	Bioinformatics Solutions	N/A
Progenesis QI v2.0 for proteomics	Waters	N/A
NetMHC4.1	https://services.healthtech.dtu.dk/services/NetMHCpan-4.1/	N/A
WebLogo	https://github.com/gecrooks/weblogo	N/A
Universal Spectrum Explorer	https://www.proteomicsdb.org/use/	N/A
SplAdder tool	ONCOSPLICING portal; http://www.oncosplicing.com/	N/A
GraphPad Prism 10	GraphPad Software	N/A
Samtools v 3.1.0	https://github.com/samtools/samtools	N/A
IGV Viewer v 2.19.4	https://igv.org/	N/A
**Other**		
ChAdOx1-PepMInt	This study/manufactured in Viral Vector Core Facility (University of Oxford)	N/A
ChAdOx1-GFP	Viral Vector Core Facility (University of Oxford)	N/A

### Cell lines

Generation of human HCT116 E2F1 CRISPR and CAS9 control cells has been described previously (Barczak et al, 2020). Mouse CT26 cells were acquired from ATCC (CRL-2638) and were used in culture and with the ChAdOx1-based vaccine immunogenicity and tumour challenge experiments. HCT116 cells were cultured in Dulbecco’s modified Eagle medium (DMEM, Sigma-Aldrich) supplemented with 10% foetal bovine serum (FBS, Labtech) and 1% penicillin/streptomycin (Gibco), whereas CT26 cells were cultured in RPMI 1640 Medium (Sigma-Aldrich), also supplemented with 10% FBS and 1% penicillin/streptomycin. PANC1 and MCF7 cells were acquired from ATCC (CRL-1469 and HTB-22, respectively) and cultured in DMEM supplemented with 10% FBS and 1% antibiotic mixture. Neuroblastoma cell lines, GIMEN (RRID: CVCL_1232) and SHEP2 (RRID: CVCL_0524) (Bate-Eya et al, 2025), were maintained in DMEM supplemented with 10% foetal bovine serum, 1% penicillin/streptomycin, 2 mM l-glutamine (Gibco), and 1 mM sodium pyruvate (Gibco). All cell lines were tested for mycoplasma contamination before use and authenticated by STR genotyping. Selective PRMT5 inhibitor (T1-44) has been described and characterised previously (Barczak et al, 2020), and was used for 48 h at 1 μM final concentration unless otherwise stated.

### RNA-seq

HCT116 and HCT116 E2F1 Cr RNA-seq datasets used in this study have been previously deposited to the GEO under accession code GSE142430 (Barczak et al, 2020). CT26 sample RNA-seq datasets have been previously deposited to GEO under accession code GSE181401 (Barczak et al, 2023). This paper also analyses existing, publicly available dsRNA J2 RIP-seq data accessible in GEO under accession code GSE174374 (Zhang et al, 2022b).

For polysome profiling RNA-seq, total RNA isolated from polysome fractions of HCT116 cells treated with T1-44 or DMSO (please see the “Polysome profiling” section of “Methods”) was isolated using the Direct-zol RNA MiniPrep kit (Zymo Research) according to the manufacturer’s instructions. RNA from HCT116 cells was used to prepare libraries by BGI Genomics. Briefly, mRNAs were isolated using oligo(dT)-based mRNA enrichment. mRNA was fragmented and reverse transcribed with random primer, prior to second-strand synthesis with dUTP, end repair and 3’ A-tailing. This was followed by adaptor ligation and digestion of U-labelled second-strand template with UDG, prior to PCR amplification. Library quality control was performed before circularisation and amplification to make DNA nanoballs. The RNA sequencing was carried out using DNBSeq-G400. The Polysome profiling RNA-seq data generated in this manuscript have been deposited in the Gene Expression Omnibus (GEO) under accession code: GSE327671.

### RNA-seq analysis

Retained intron (RI) prediction was performed with VAST software (Tapial et al, 2017) (version 2.5.1), and VAST database (version 23.06.20) based on the mouse genome mm10 and the human genome hg19. A differential alternative splicing assessment was performed by the vast-tools “diff” module, and the results were filtered with dPSI (delta psi; difference in psi values between treatments) threshold of 0.01. For all genes, including RIs, predicted by VAST, de novo assembly of transcripts was performed (Trinity software package, version 2.15.1). Sashimi plots were generated using IGV Viewer (v 2.19.4). For all the transcripts, levels of expression were estimated by kallisto software (0.44.0).

For rMATS analysis, the processed reads were aligned with the human reference genome hg19 and murine reference genome mm10 using the STAR package (v.020201) (Dobin et al, 2013). The aligned data were used for differential alternative splicing assessment with the rMATS program (v4.0.2) (Shen et al, 2014).

For the generated polysome profiling RNA-seq and publicly available dsRNA J2 RIP-seq GSE174374 (samples: GSM5293215, GSM5293216, GSM5293217, GSM5293218, GSM5293219, GSM5293220), the cleaned and processed reads were aligned with the human reference genome hg19 using the STAR package. The number of RNA-seq reads mapping to annotated peptide-coding intronic regions in polysome-associated RNA from DMSO- and T1-44-treated HCT116 cells, and dsRNA J2 RIP-seq was calculated using the samtools kit (v 3.1.0). Sashimi plots were generated using IGV Viewer (v 2.19.4).

### Heatmap generation and normalisation

For heatmap analyses, retained intron (RI) events were selected based on differential splicing (*P* < 0.05) or a minimum level of 10% PSI in the wild-type condition. For peptide-level analyses, intensity values were used. For visualisation, PSI values for each RI event and peptide intensity values were normalised across conditions. Specifically, values were divided by the mean across all conditions for each RI or peptide, enabling comparison of relative changes independently of absolute levels. The normalised values were then used to generate heatmaps with the pheatmap R package (version 1.0.13).

### Gene ontology analysis

Gene ontology of previously analysed differentially expressed genes (GSE142430, GSE181401) was performed using the goseq R package (Young et al, 2010).

### qPCR

RNA was isolated from cells using TRIzol (Thermo Fisher Scientific) or the Direct-zol RNA MiniPrep kit (Zymo Research) according to the manufacturer’s instructions. In total, 1 μg of total RNA was used for complementary DNA (cDNA) synthesis. Reverse transcription with oligo (dT)20 (Invitrogen) was performed using SuperScript III Reverse Transcriptase (Invitrogen) as per the manufacturer’s instructions. Quantitative PCR (qPCR) was then carried out in technical triplicate using the indicated primer pairs and the Brilliant III SYBR Green qPCR Master Mix (Stratagene) on an AriaMx (Agilent) instrument. Results were expressed as average (mean) fold change compared to control treatments using the ΔΔCt method from at least three biological repeat samples. Glyceraldehyde-phosphate dehydrogenase (GAPDH) primer sets were used as an internal calibrator. Error bars represent SD unless otherwise indicated. For primer lists, please see the Reagents and Tools Table.

### dsRNA prediction and structure stability

The secondary structure analysis of the RI-containing transcripts was predicted with the ViennaRNA package (Lorenz et al, 2011) (version 2.4.18) with default parameters. As a result of the run, the RNAfold programme produces a text file with an ASCII-depicted model of complementary regions in a given RNA sequence. The file was processed by a custom Python script to extract the value of MFE (minimum free energy) and the number and lengths of double-stranded regions. In addition, the number of paired nucleotides in the predicted double-stranded regions was calculated by RSCanner. In addition, to assess the prevalence and the structural stability within predicted RI dsRNA sequences using Adjusted Minimum Free Energy (AMFE) according to Trotta et al (2014) (Trotta, 2014).

### ENCODE ChIP-seq data analysis

Gene promoter characterisation was performed utilising bioinformatics tools present in UCSC Genome Browser (https://genome.ucsc.edu; GRCh37/h19 assembly) and analysing ChIP-seq data for E2F1 tracks from the ENCODE project (http://genome.ucsc.edu/ENCODE/). The ‘Txn factor ChIP track’, ‘ENCODE 3 TFBS Track’, ‘Uniform TFBS track’ and ‘SYDH TFBS track’ tools were used to display E2F1 ChIP-seq peaks or signal as appropriate.

For promoter characterisation in the mouse, the GRCm38/mm10 assembly was used, and mouse E2F1 ChIP-seq peak data were loaded as a custom track using data deposited in GEO (GSM288349). ChIP-seq peak coordinates were intersected with 1000 bp wide regions around TSS (GENCODE annotation).

### Flow cytometry

For intracellular staining of dsRNA and IFN-β, cells were fixed and permeabilised using the Cytofix/Cytoperm kit (BD Biosciences, 554714). Cells were incubated with Fixation/Permeabilisation solution at 4 °C for 15 min, washed twice with 1× BD Perm/Wash buffer, and stained with FITC-conjugated IFN-β antibody (1:100, Invitrogen, BMS1044F1) and J2 dsRNA antibody (1:700, Nordic Biosite, 246-10010500) for 1 h at 4 °C. For dsRNA staining, anti-mouse Alexa Fluor 488 conjugated secondary antibody (1:800, Invitrogen, A-11059), and samples were incubated for 1 h in the dark. Then, samples were run and analysed on a flow cytometer (BD Accuri C6 Plus).

For HLA-ABC surface staining, HCT116 cells were seeded onto a six-well plate at 50,000/well and left to adhere overnight. The next day, IFN receptors were blocked by incubation with anifrolumab (IFNAR, Medchemexpress, #HY-P99168) at 10 µg/mL for 2 h prior to remaining treatments. Then the cells were treated with 20 µM T1-44 and 60 ng/mL IFN-β (R&D Systems, #8499-IF-010) for 5 days. After that, the cells were harvested, washed twice and stained with HLA-ABC antibody (BD Biosciences, #560965) according to the manufacturer’s protocol. The mean fluorescence intensity was recorded using BD Accuri C6 plus (BD Bioscience, San Jose, CA, USA).

To assess whether the T1-44-mediated increase in HLA-ABC expression was dependent on a transferable factor released into the medium, the effect of T1-44-conditioned medium on HLA-ABC surface expression was tested. About 100,000 HCT116 cells were seeded onto a six-well plate and left to adhere for 6 h. Then, the cells were treated with 1.5 μM T1-44 or DMSO for 24 h. After that, the medium was removed, and cells were washed three times with PBS before adding 2 mL fresh medium for conditioning over the next 48 h. Then, HCT116 cells were seeded on a 12-well plate at 35,000/well and left to attach, after which 1 mL of conditioned medium was added to each well. The medium conditioned by cells treated with DMSO served as a negative control, and the positive control was DMSO-conditioned medium supplemented with 10 ng/mL IFN-β (R&D Systems, #8499-IF-010). The cells were incubated with conditioned medium for 72 h prior to collection for HLA-ABC surface staining.

### Western blot

For immunoblots, cells were harvested in modified RIPA buffer (50 mM Tris-HCl pH 7.5, 150 mM NaCl, 1% Igepal CA-630 [v/v], 1 mM EDTA, 1 mM NaF, 1 mM Na_3_VO_4_, 1 mM AEBSF, protease inhibitor cocktail) and incubated on ice for 30 min prior to SDS–PAGE and transfer to nitrocellulose. The following antibodies were used in immunoblots: β-actin (clone AC-74, Sigma-Aldrich; dilution 1:2000), E2F1 (3742S, Cell Signaling Technology, dilution 1:1000) and symmetric di-methyl arginine (SDMe,13222S, Cell Signaling Technology, dilution 1:1000).

### Immunofluorescence

Cells were seeded onto eight-well cell culture chambers on PCA slides (Sarstedt, 94.6140.802) 24 h after the second round of plasmid transfection, followed by drug treatment for 48 h. Cells were then fixed in 4% paraformaldehyde for 20 min at room temperature, and washed before permeabilised with 0.5% Triton X-100 in PBS for another 20 min at room temperature. All washes are done three times with 1× PBS.

As a negative control for dsRNA staining, one well was treated with 20U RNase III (Invitrogen, AM2290) for 30 min at 37 °C before proceeding to blocking with 1% BSA in PBS-T for 30 min at room temperature. The slides were then incubated overnight in a humidified chamber at 4 °C with J2 dsRNA primary antibody (1:700, Nordic Biosite, 246-10010500).

Slides were washed before incubation with anti-mouse Alexa Fluor 488 conjugated secondary antibody (1:800, Invitrogen, A-11059) for 1 h in the dark in a humidified chamber. After the final wash, slides were fixed using Fluoroshield mounting medium with DAPI (4’,6-diamidino-2-phenylindole) (ab104139). Results were analysed using a Leica DM2500 optical microscope (Wetzlar, Germany) and presented as semi-quantitative using ImageJ software (National Institutes of Health).

### Immunohistochemistry

To assess the effects of PRMT5 inhibition on dsRNA formation and IFN-β induction, Balb/c mice were treated with T1-44 as previously described (Barczak et al, 2023). FFPE slides were washed for 5 min with Histochoice (Sigma-Aldrich), followed by two times 3 min washing in 100% ethanol, 3 min in 70% ethanol and 5 min in tap water. Next, samples were incubated with antigen retrieval solution (sodium citrate buffer or Tris/EDTA, depending on the antibody used) at 99 °C in a water bath for 20 min. After three times washing with purified water, samples were incubated with freshly made 6% Methanol/H2O2 for 15 min, and washed in tap water. In the next steps, slides were washed in 1% PBS-T for 5 min, blocked in blocking serum solution (Vectastain ABC kit) for 20 min, washed again in 1% PBS-T for 5 min and incubated overnight at 4 °C with primary J2 dsRNA antibody (1:3000, Nordic Biosite, 246-10010500) or for 1 h at room temperature with IFN-β antibody (1:400, Thermo Fisher Scientific, PA5-20390). For the negative control, no primary antibody and RNAse III pre-treatment were used. As the J2 dsRNA antibody used for dsRNA detection is a mouse monoclonal antibody, the Mouse-on-Mouse Polymer IHC Kit (Abcam, ab269452) was used for subsequent blocking of mouse IgG for 1 h at room temperature before the primary antibody incubation step. The next day, slides were washed with 1% PBS-T for 5 min followed by 30 min incubation with secondary antibody (Vectastain ABC kit) at room temperature. In the next step, the ABC solution (Vectastatin ABC-HRP Kit, Peroxidase, Rabbit IgG, PK-4001) was added for 30 min, and the slides were washed in 1% PBS-T and incubated with DAB solution (Vector DAB) for 10 min. Then, slides were washed in purified water and counterstained in haematoxylin (Sigma-Aldrich). The slides were imaged using a Leica microscope (at least two images from the centre and/or the margin from each sample were taken), and the results were analysed by calculating the mean of the optical density in each group. The data were then semi-quantitated using ImageJ v1 software (Fiji package) (National Institutes of Health).

### Plasmid/siRNA transfection

The RI sequence and/or flanking exons were cloned into pcDNA3.1(+) plasmid vector (Genscript). Plasmid transfections were performed using the GeneJuice transfection reagent (Novagen, 70967). In all, 1.5 μL of GeneJuice per μg of DNA was added dropwise to Opti-MEM (Gibco, 31985070) and incubated for 5 min at room temperature. Then, DNA was added to the GeneJuice/Opti-MEM mixture and mixed gently. The mixture was incubated for a further 15 min before addition to the dishes with pre-seeded cells. The medium was replaced 6 h after transfection. The plasmid transfection was repeated on day 3. Cells were collected 48–72 h after the second transfection.

RNA interference was performed with 25 nM siRNA for 72 h using the Oligofectamine transfection reagent (Invitrogen), as per the manufacturer’s instructions. Sequences for siRNA are as follows: non-targeting control, 5′-AGCUGACCCUGAAGUUCUU-3′; while siPKR was obtained from Sigma-Aldrich (EHU127861).

### In vitro transcription (IVT) and RNA immunoprecipitation (RIP)

#### Preparation of PCR templates

The pcDNA3.1(+) plasmids (Genscript) containing either intron-retained or control sequences of DDX55, CDK10, HDAC6 and A1BG were linearised with restriction enzymes: ApaI or BclI (Thermo Scientific). PCR of the target sequences, including the T7 promoter region, was performed using Phusion™ High-Fidelity DNA Polymerase (Thermo Scientific, F-530XL). 20 ng of linearised plasmids were used to set up 50 μL reactions following the manufacturer’s instructions. The thermocycling conditions were as follows: denaturation at 98 °C for 30 s, 98 °C for 10 s, 61 °C annealing temperature for 30 s and 72 °C for 1 min to a total of 35 cycles, and a final 72 °C elongation for 5 min. PCR products were visualised on 1% agarose gel with GelRed Nucleic Acid Stain, excised and extracted from the agarose gel using QIAquick Gel Extraction Kit (Qiagen, 28704) as per the manufacturer’s instructions.

#### In vitro transcription (IVT)

IVT was performed using the T7-Scribe™ Standard RNA IVT Kit according to the manufacturer's instructions (Cellscript, C-AS3107). The RNA was resuspended in RNase-free water and quantified using the Nanodrop 2000 Spectrophotometer (Thermo Scientific). 5’ cap and Poly A tail were added to the IVT products. The ScriptCap™ m7G Capping System (Cellscript, C-SCCE0625) and the ScriptCap™ 2’-O-Methyltransferase Kit (Cellscript, C-SCMT0625) were used for capping. RNA products were visualised by running 100 ng of RNA in a 1% agarose gel prepared using 1× Borax buffer and GelRed Nucleic Acid Stain (Merck, SCT123). An ssRNA Ladder (New England Biolabs, N0362S) was run alongside for size reference, and RNA was denatured by mixing with 2× RNA Loading Dye, heated at 70 °C for 10 min and then placed on ice. Samples were loaded onto an agarose gel and run at 150 V for 20 min before visualisation using a ChemiDoc Imaging System (Bio-Rad).

#### dsRNA immunoprecipitation

Protein A agarose beads were pre-blocked with 5 mg/mL BSA and 300 μg/mL salmon sperm DNA for 1 h. Then the beads were washed and resuspended in RIP lysis buffer (50 mM Tris-HCl pH 7.4, 100 mM NaCl, 1% Igepal, 0.1% SDS, 0.5% sodium deoxycholate, 1× protease inhibitor cocktail), and kept at 4 °C. For all the following procedures, RNaseOUT (Invitrogen, 10777019) was added to the RIP buffer at a final concentration of 200 U/mL to prevent degradation of RNA.

Equal amounts of transcribed RNA (30–60 μg) were made up to 1 mL with RIP buffer, 60 μL of pre-blocked protein A/G agarose beads, and 2 µg of mouse IgG for pre-clearing of samples. The mixtures were rotated at 4 °C for 2 h.

Beads were then spun down at 360× *g* at 4 °C for 3 min, and 1/10 of the supernatant was kept as input and stored at −20 °C. The rest of the supernatant was split equally into two tubes and topped up to 1 mL with RIP buffer. In total, 2 µg of either J2 dsRNA antibody (Nordic Biosite, 246-10010500) or control, mouse IgG antibody, were added to the samples. The samples were incubated at 4 °C on a rotating mixer overnight.

The next day, 60 μL of A/G beads were added to each tube, and samples were incubated for a further 2 h. Then, the beads were washed four times with RIP buffer and resuspended after the fourth wash with TRIzol (Thermo Fisher Scientific, 15596026) to elute RNA from the beads.

Then RNA isolation from the beads and input samples was performed using TRIzol according to the manufacturer’s instructions. During RNA precipitation, 15 μg of GlycoBlue™ Coprecipitant (Invitrogen, AM9516) was used as a carrier for RNA to increase the visibility of RNA pellets.

RT-qPCR was performed as described (please see “qPCR” section of “Methods”), but instead of Oligo(dT)12-18 Primer (Invitrogen, 18418012), Random Hexamers (Invitrogen, N8080127) were used for reverse transcription. The Intron and Exon primers used for qPCR are listed in the Reagents and Tools Table.

The results were calculated in the following way. For each gene, both RI and Exon primers were used. The fold enrichment over Exon for each primer set = 2^-(average Ct value of J2 from RI construct − average Ct value of J2 from Exon construct).

### Immunopeptidomics

Antibodies were sourced from hybridoma supernatants (ATCC, HB-95 and −79, respectively) using a standard purification procedure using Sepharose-protein A beads (Expedeon). 0.5 mL/sample beads were incubated with 5 mg/sample of W6/32 antibody (specific for HLA class I for HCT116), or antibody clone 34.1.2 s (recognising H-2 Kd, Dd, Ld for CT26), for 30 min at room temperature. The resin was washed with 10 cv (column bed volumes) of borate buffer (50 mM borate, 50 mM KCl, pH 8.0), and antibodies were cross-linked by adding 10 cv of 40 mM dimethyl pimelimidate in borate buffer (pH 8.3) for 30 min at room temperature. The reaction was stopped with 10 cv of ice-cold 0.2 M Tris, pH 8.0, followed by a washing step of 10 cv of 0.1 M citrate, pH 3.0, to remove any unbound antibody, and finally equilibrated with 10 cv of 50 mM Tris, pH 8.0.

Cell pellets were lysed in 3 mL lysis buffer (1% Igepal CA-630; 100 mM Tris, pH 8.0; 300 mM NaCl; supplemented with complete Protease Inhibitor Cocktail, EDTA-free, [Roche]) by mild agitation. Samples were incubated for 45 min on ice. Lysates were then cleared by sequential centrifugation steps at 500× *g* for 10 min then 20,000× *g* for 1 h at 4 °C. Peptide-HLA class I complexes were captured by overnight incubation with the antibody-coated beads at 4 °C under mild agitation. The lysate was then removed by gravity flow, and the column was washed consecutively with 10 mL wash buffer 1 (0.005% Igepal, 50 mM Tris pH 8.0, 150 mM NaCl, 5 mM EDTA), 10 mL wash buffer 2 (50 mM Tris pH 8.0, 150 mM NaCl), 10 mL wash buffer 3 (50 mM Tris pH 8.0, 450 mM NaCl) and 10 mL wash buffer 4 (50 mM Tris pH 8.0). Peptide-HLA complexes were eluted by the addition of 5 cv of 10% acetic acid.

#### HLA peptide purification strategies

Samples were loaded onto an Ultimate 3000 HPLC system (ThermoFisher Scientific), and peptides were separated from larger complex components using a monolithic column (4.6 × 50 mm ProSwift RP-1S, ThermoFisher Scientific) by applying a 10 min gradient from 2 to 35% buffer B (0.1% TFA in acetonitrile) with a flow rate at 1000 µl/min. Each sample was fractionated into 15 fractions, and alternate fractions containing the HLA peptides but not ß2-microglobulin were pooled in two final fractions. Samples were dried, resuspended in 20 µl of loading buffer (0.1% TFA, 1% ACN) and stored at −80 °C prior to MS analysis. LC-tandem mass spectrometry (LC-MS/MS).

For HCT116 cell samples, HLA peptides were analysed by either an Orbitrap Fusion Lumos Tribrid mass spectrometer (Thermo Scientific) or a Q Exactive HF-X mass spectrometer (Thermo Scientific). CT26 cell samples were measured on a Q Exactive HF-X (Thermo Scientific). Either mass spectrometer instrument was coupled with an Ultimate 3000 RSLCnano System supplemented with a PepMap C18 column, 2-µm particle size, 75 µm × 50 cm (Thermo Scientific). Peptides were eluted using a 60 min linear gradient of 3–25% acetonitrile in 5% DMSO, 0.1% formic acid in water at a flow rate of 250 nl/min and 40 °C and introduced into the mass spectrometer using a nano EASY-Spray source at 2000 V (Thermo Scientific). The ion transfer tube was set to 305 °C for both instruments.

For samples analysed by the Orbitrap Fusion Lumos, the resolution for full MS was set at 120,000 with ACG target of 400,000 and scan range of 300–-1500 *m/z*. Precursor selection and isolation were performed using TopSpeed in a 2 s cycle time and 1.2 amu quadrupole isolation width. MS2 resolution was set at 30,000, and peptide ions were accumulated at a maximal injection time of 120 ms with an AGC target of 300,000. Precursor ions were fragmented using high-energy collisional dissociation: Collision energy was set to 28 for peptides with a charge state of 2–4, and set to 32 for singly charged ions. For samples analysed on the Q Exactive HFX, full MS (320–1600 *m/z* scan range) resolution was set at 120,000, and an AGC target of 300,000. Peptide ions were isolated at 1.6 amu isolation width. MS2 resolution was set to 60,000 at an AGC target of 50,000, and the collision energy was set at an energy of 28 for peptides with a charge state of 2–4, fragmentation of precursor ions and 25 for those with a charge state of 1–4.

#### Data processing for liquid chromatography mass-spectrometry analysis (RI-derived peptide databases)

The nucleotide sequence of predicted RIs (differentially spliced events detected by the diff module of the VAST tool algorithm) was translated into proteins with seqkit (Shen et al, 2024) using 3-frame translation. For databases used, please see Datasets EV1 and EV3.

#### Mass spectrometry data analysis

MS data were analysed with Peaks v8.5 (Bioinformatics Solutions) for identification of peptide sequences matching to databases generated by integration of all reviewed human SwissProt protein entries (20,386 entries, current at 09/06/2022), or all reviewed mouse SwissProt protein entries (17,228 entries, current at 03/08/2023), combined with the respective three frame translations of the open-reading frames obtained from the in-house RNA sequencing assemblies generated for the HCT116 data and CT26 cell lines, respectively. Searches were performed with the following parameters: no enzyme specificity, no peptide modifications, peptide tolerance: ±5 ppm and fragment tolerance: ±0.03 Da. The results were filtered using a false discovery rate of 4.3% and 5.8% established through parallel decoy database searches for HCT116 and CT26 data, respectively. For quantitative analysis, the data were analysed by Progenesis QI v2.0 for proteomics (Waters). A one-way ANOVA analysis was applied to assess the significant regulation of peptides between conditions. GraphPad Prism (GraphPad Software Inc) was used for visualisation of the data. HLA class I peptides prediction was performed using the NetMHC4.1 online algorithm (Reynisson et al, 2020) and WebLogo (Crooks et al, 2004). HCT116 and CT26 datasets are available via ProteomeXchange (PRIDE database) with identifiers PXD029613 and PXD029594, respectively.

Identified endogenous peptides were validated by spectrum comparison with synthetic peptides (Genscript). M/Z and intensity data were collected from raw files by the rawrr R package and analysed with Universal Spectrum Explorer (https://www.proteomicsdb.org/use/).

### RNA immunoprecipitation

Cells were washed once with phosphate-buffered saline (PBS) before ultraviolet cross-linking at 4,000 Units using a Stratalinker (Stratagene). Cells were collected, washed with PBS and resuspended in 3 ml of PBS. 90 μl of Formaldehyde was added, mixed and incubated at RT for 15 min. In all, 1 ml 0.5 M Glycine was added, mixed and incubated for another 5 min. After washing, RIP lysis buffer [50 mM tris-HCl (pH 7.4), 100 mM NaCl, 1% (v/v) NP-40, 0.1% sodium dodecyl sulphate, 0.5% sodium deoxycholate, and protease inhibitor cocktails] was added, incubated for 10 min on ice, and centrifuged at 13,000 rpm (5 min, 4 °C). For RNA samples, 10% of inputs were taken, and 10 μg of proteinase K was added for 30 min at 37 °C before addition of TRIzol and RNA isolation. The rest of the lysate was precleared using preblocked protein A/G agarose beads, 2 μg of nonspecific IgG (Jackson ImmunoResearch), and heparin (0.1 mg/ml) for 2 h at 4 °C. The precleared lysate was added to a fresh tube with 2 μg of nonspecific IgG or a specific antibody (dsRNA; J2) for overnight incubation at 4 °C with rotation. Protein A/G beads were then added for a further 2 h. The beads were washed four times in RIP lysis buffer and resuspended in 200 μl of RIP lysis buffer for reverse cross-linking and elution (70 °C, 1 h). After centrifugation, 500 μl of Trizol was added for RNA isolation.

### Polysome profiling

Cells were treated with 100 µg/mL cycloheximide for 10 min at 37 °C, treated with 1× trypsin-EDTA solution for 10 min and washed twice with ice-cold 1× PBS containing 100 µg/mL of cycloheximide. Polysome lysis buffer comprising 20 mM Tris HCl pH 7.4, 5 mM MgCl_2_, 100 mM KCl, 100 μg/mL cycloheximide, 1% Triton X-100, 1× RNase inhibitor (Invitrogen), and 1× protease inhibitors (VWR) was used to resuspend cells, followed by 30 min incubation on ice (occasional inverting) and 10 min centrifugation at 12,000×* g* at 4 °C. Sucrose gradients were prepared using 10%, 20%, 30%, 40% and 50% sucrose solutions (sucrose diluted in polysome extraction buffer without Triton X-100 and prepared in RNAse-free conditions) in a polypropylene, 13.2-mL tube (Beckman Coulter). The gradient was left at 4 °C overnight to become linear. Clear cell lysates, containing 300 µg of RNA, were loaded onto the sucrose gradients and centrifuged at 190,000× *g* (SW40Ti rotor, Beckman Coulter Optima XE) for 90 min at 4 °C. Twelve fractions were separated by manual collection, and the absorbance was measured at 254 nm to record the polysome profile.

### ChAdOx1 vector preparation

The ChAdOx1-PepMInt and ChAdOx1-GFP adenoviruses were manufactured by the Viral Vector Core Facility (Jenner Institute, University of Oxford). The poly-antigen cassette, containing the MHC class I presented peptides derived from RIs (Appendix Fig. S6) was designed to include each peptide sequence plus 12 base pairs of natural flanking upstream and downstream sequence; which means for SPVLIHSFL and SPGDLGLSL peptides, sequences coding KHATSPVLIHSFLKVGP and FIIPSPGDLGLSLGCVV respectively, were designed. Coding sequences for all peptides were combined into the poly-antigen cassette. The tPA sequence was also added to the 5′ end of the cassette in addition to a Kozak sequence. The cassette was synthesised in the pC1921 24ABNBMP_PepMlnt_pMA-RQ plasmid (Thermo Fisher Scientific) by GeneArt Gene Synthesis. pC1921 24ABNBMP_MuPeplnt_pMA-RQ and p1990 (the entry vector containing a long CMV promoter) plasmids were digested with KpnI and NotI to obtain the antigen insert and the entry backbone, respectively. DNA was then ligated and transformed into DH10b cells. Bacterial clones were colony screened by PCR, and a clone was selected for plasmid purification using midi-prep (Qiagen). This entry clone was named pENTR4LPTOS-PepMlnt. pENTR4LPTOS-PepMlnt and the destination shuttle vector p2563 (ChAdOx1) were recombined using LR Clonase II (Thermo Fisher Scientific) and transformed into DH10b cells. Clones were screened by antibiotic sensitivity and colony PCR to select a single clone for BAC prep. This resulting pChAdOx1-PepMInt plasmid was sequenced to confirm its identity and digested to linearise the plasmid, as per standard protocols. Linearised pChAdOx1-PepMInt was used for virus production in HEK293A T-Rex cells. The presence of the antigen was confirmed by ID PCR. The integrity of the antigenic DNA sequence and absence of contaminating Adenovirus were confirmed by Flank-Flank PCR.

### Mouse experiments—general information

Sample size - size of the groups was determined based on power calculations to reach >80% power for the outputs required, and previous experience. Replication—ChAdOx1-PepMInt tumour challenge and immunogenicity experiment were performed twice with successful replication. The ChAdOx1-PepMInt/T1-44 tumour challenge experiment has been performed once. Randomisation—animals were randomly assigned to treatment or control groups. Blinding—no blinding was used since all mice/cages were identified by a label and number to ensure proper treatments. All animal experiments were performed in compliance with institutional guidelines and relevant ethical regulations.

### Mouse experiment—immunogenicity

Five 7-week-old female BALB/c mice (Charles River Laboratories) were vaccinated intravenously with ChAdOx1-PepMInt adenoviral vectors (2.5 × 10^8^ IU). Another five control mice were vaccinated with the equivalent dose of the ChAdOx1-GFP vector. At day 10 post-vaccination, mice were culled and their spleens collected for the ELISpot assay. All animals were housed in specific pathogen-free conditions at the Biomedical Services Building (University of Oxford). All work was performed under UK Home Office license PPL PP3430109 in accordance with the UK Animal (Scientific Procedures) Act 1986. The work was performed by trained and licensed research staff. The experiment was performed twice with successful replication. In the manuscript, one representative experiment is shown.

### ELISpot

The day before endpoint, Merk Multiscreen 96-well Filter Plates (Merck) were incubated with primary antibody (IFNγ mAb clone AN18, Mabtech, 3321-3-1000) diluted 1:200 in sterile PBS (Gibco), at 4 °C. The next day, the antibody was removed, plates were washed four times with PBS at 200 µl/well, then blocked with 200 µl/well medium (RPMI [Gibco] supplemented with 10% heat-inactivated FCS, Non-essential amino acids, L-Glutamine and penicillin/streptomycin (all from Sigma) for 2 h at 37 °C). Mice were culled, their spleens removed, passed through a 40 µm cell strainer (Falcon) and the single cell suspension pelleted by centrifugation. The splenocytes were resuspended in 1 mL ACK lysis buffer (Lonza) for 1–3 min to lyse the red blood cells, then stopped with 5 mL medium, followed by centrifugation at 400×* g*, 5 min at room temperature. The splenocyte pellet was resuspended in 5 mL medium, counted and the cell concentration adjusted to 4 × 10^6^/mL. Blocking buffer was removed and replaced with 50 µl of cells, which were stimulated with the respective individual peptides (50 µl of peptide at a final concentration of 10 µg/mL) that the group had been vaccinated with. Negative control wells contained DMSO only, while cells were stimulated with PHA-L (11249738001, Roche, dilution 1:200) as a technical control. The plates were incubated overnight (15–20 h) in a 37 °C (5% CO_2_) incubator. The cells and peptides were removed, and the wells were washed seven times with sterile PBS. Secondary antibody (biotin conjugated anti-IFNγ, MabTech, 3321-6-100, clone R4-6A2-Biotin) diluted 1:2000 in assay diluent (AD) (25 mg/mL BSA in PBS), was added (50 µl/well) and incubated for 4 h at room temperature. The plates were then washed four times with PBS, then 50 µl of streptavidin-alkaline phosphatase (Mabtech, 3310-10-1000) diluted 1:1000 in AD was added and incubated for 2 h at room temperature. Plates were washed four times with PBS, then 50 µl BCP/NBT substrate was added to each well and allowed to develop for 5–10 min until spots were visible in the positive control wells. The reaction was stopped by rinsing the plates in water three times. The rubber bottom was removed, and the membrane was rinsed on both sides with water, then allowed to dry. The spots were imaged and quantitated on an ELISpot Reader (S6 Entry M2, ImmunoSpot by C.T.L.).

### PepMInt tumour challenge experiment

Six female BALB/c mice at 7 weeks of age (Charles River Laboratories) were vaccinated i.v. with ChAdOx1-PepMInt adenoviral vectors (2.5 × 10^8^ IU). Another six control mice were vaccinated with the ChAdOx1-GFP vector. At day 10 post-vaccination, the mice received unilateral subcutaneous injections of 2 × 10^5^ CT26 cells in PBS in a total injection volume of 100 µl/mouse. Body weights and tumour volume [mm^3^] by calliper measurement were performed twice weekly. After reaching 300 mm^3^ tumour volume (calculated according to the formula: ((length × width^2^)/2), mice were monitored daily. Termination of individual mice was conducted at day 17 (day 19 in the second experiment) post-implantation or at tumour volume not exceeding 1200 mm^3^ (unilateral). All animals were housed in specific pathogen-free conditions at the Biomedical Services Building (University of Oxford). All work was performed under UK Home Office licence PPL PP3430109 (Protocol 2) in accordance with the UK Animal (Scientific Procedures) Act 1986 and was approved by the Animal Care and Ethical Review Board at the University of Oxford. All work was performed by trained and licensed individuals. Housing conditions were as follows: temperature: 22–24 °C, 12 h day/night cycle, humidity 40–70%. We performed the experiment twice with successful replication.

### PepMInt and T1-44 tumour challenge experiment

Twelve female BALB/c mice at 7 weeks of age (Charles River Laboratories) were vaccinated i.v. with ChAdOx1-PepMInt adenoviral vectors (2.5 × 10^8^ IU). Another twelve control mice were vaccinated with the ChAdOx1-GFP vector. At day 9 post-vaccination, the mice received unilateral subcutaneous injections of 2 × 10^5 CT26 cells in PBS in a total injection volume of 100 µl/mouse. From day 7, half of the mice from each group were treated daily (for 10 days) by oral administration (gavage) with 100 mg/kg (dosing volume 10 ml/kg) of T1-44 dissolved in 0.5%Tween80 in 5% DMSO/PBS. Body weights and tumour volume [mm^3^] by calliper measurement were performed twice weekly. After reaching 500 mm^3^ tumour volume (calculated according to the formula: ((length × width^2^)/2), mice were monitored daily. Termination of individual mice was conducted at day 18 post-implantation. All animals were housed in specific pathogen-free conditions at the Biomedical Services Building (University of Oxford). All work was performed under UK Home Office licence PPL PP6528177 (Protocol 2) in accordance with the UK Animal (Scientific Procedures) Act 1986 and was approved by the Animal Care and Ethical Review Board at the University of Oxford. All work was performed by trained and licensed staff. Housing conditions were as follows: temperature: 22–24 °C, 12 h day/night cycle, humidity 40–70%.

### Human participants

All experiments involving human samples were conducted in accordance with the principles of the World Medical Association Declaration of Helsinki and the Department of Health and Human Services Belmont Report, as well as relevant institutional and national ethical regulations. The blood samples were collected by TGLU (Translational Gastroenterology and Liver Unit) Biobank under the research tissue bank ethics 21/YH/0206. Informed consent was obtained from all participants. Blood donors were patients diagnosed with colorectal cancer. The population contained 34 patients, 24 of whom were male and 10 female. The age at the time of blood donation ranged from 34 to 91 years old, with a median age of 75.5. Gender was not reported. Details of the race or ethnicity of blood donors were not collected.

### T-cell proliferation assay

PBMCs were isolated with Ficoll-Paque Plus according to the manufacturer’s protocol. PBMCs were washed twice with 1 mL of PBS, followed by loading with Cell Tracker Far Red (C34572, ThermoFisher Scientific) at a 1:1000 dilution for 10 min at room temperature. The loading reaction was quenched with 4 mL of foetal bovine serum (FBS) at 4 °C, and cells were resuspended in RPMI medium supplemented with 10% human blood group type AB serum (Sigma), 1% penicillin-streptomycin solution, and 2 mM L-glutamine solution, and subsequently plated in a 96-well round-bottom plate at a plating density of 0.25 × 10^6^ cells per well. Cells were stimulated with single peptides (Appendix Fig. S11A) at a final concentration of 10 μg/mL per peptide. Medium, containing 0.1% dimethyl sulfoxide (DMSO; Sigma) representing DMSO content in peptide solution, was used as a negative control, and 2 μg/mL of PHA-L (Sigma) was used to stimulate proliferation as a positive control. Cells were then incubated at 37 °C, with 5% CO_2_ for 9–12 days (freshly isolated PBMCs) or 20 days (frozen PBMCs), with a change of media on day 5. At the end of the incubation period, cells were stained using the antibody panel CD3^+^, CD4^+^, and CD8^+^. All samples were acquired using a BD Accuri C6 plus (BD Bioscience, San Jose, CA, USA), and the gating strategy is shown in the Appendix Fig. S11B. Responses above the DMSO background were considered positive.

### Killing assay

Human PBMCs from donors #20, #26, and #28 were thawed and expanded with 10 µg/mL phytohemagglutinin-L (PHA-L, Roche, #11249738001) for 5 days and subsequently stimulated with IL-2 (BioLegend, #589102) at 50 U/mL twice, 48 h apart. HCT116 cells were seeded onto 96-well E-plate (Agilent, #300601020) at 10,000/well and placed onto xCELLigence Real-Time Cell Analysis reader (Agilent) for baseline recordings taken at 15 min intervals for 4 h (or 24 h when enriched T cells were used). Then, the experiment was paused, and 100,000 PBMCs or T cells (see “T-cell isolation and peptide pulsing” section in “Methods”) in 100 µL medium were added to treated wells, and cell-free medium was added to control wells. The xCELLigence software-generated cell index was recorded for 50 h at 15-min intervals.

### T-cell isolation and peptide pulsing

T cells were isolated from PBMC samples using MojoSort™ Human CD3 T Cell Isolation Kit as per the manufacturer’s protocol (Biolegend, 480021). Briefly, the PBMCs from donors #24, #30, and #31 were prepared, and the cell density was adjusted to 10^7^ per 100 µL in MojoSort buffer. Then, 10 µL of Biotin-Antibody cocktail was added, and the cell suspension was incubated on ice for 15 min. Cells were then resuspended by vortexing, and 10 µL of Streptavidin Nanobeads was added, followed by the addition of 2.5 mL of Mojosort buffer before the tube was placed on the magnet for 5 min. T cells were isolated by pouring the suspension into a fresh tube. T-cell enrichment was confirmed via flow cytometry (CD3^+^ CD8^+^ surface markers) and compared to pre-enrichment samples. Isolated cells were centrifuged and resuspended in complete media with the cells adjusted to 1 × 10^6^ per mL. The T-cells were activated and expanded by the addition of ImmunoCult Human CD3/CD28 T Cell Activator (25 µL per 1 mL of cell suspension, Stemcell Technologies UK Ltd, 10971) and IL-2 (50 U/mL). Isolated T cells were incubated with 10 µg/mL of PHA-L and co-stimulated with 50 U/mL IL-2 for 8 days, followed by the peptide pulse (pool of seven HLA-A02 peptides; Fig. S11A) at 10 µg/mL for 3 days and a recovery period of 72 h.

### TCGA analysis

For the analysis of selected Retained Intron transcript expression levels in human colorectal cancer, the SplAdder tool was used (ONCOSPLICING portal; http://www.oncosplicing.com/) (Zhang et al, 2022a).

### Statistical analysis

Statistical analyses were performed using two-tailed, unpaired Student’s *t* test when only two samples were being compared, whilst one-way ANOVA was used in experiments involving multiple comparisons (with GraphPad Prism 10 Software). Data are shown as means with SD, unless otherwise indicated. *P* values lower than 0.05 were considered significant and are labelled using asterisks *P  <  0.05, **P  <  0.01, ***P  <  0.001, and ****P  <  0.0001, and the numerical *P* values can be found in the Table EV1. The exact number of biological replicates is given in every figure legend.

For animal experiments, mice of the appropriate genotype, age, sex, and health status were included and randomly assigned to experimental groups where applicable. Mice were excluded only if tumour implantation failed.

For human sample studies, participants were included if they had pathologically confirmed colorectal cancer. Participants were excluded if they had received preoperative antitumour treatment.

For cellular experiments, no data points were omitted unless a predefined technical failure occurred and was documented.

### Graphics

Visual abstract and schematic illustration in Figs. 3A and 5C were created with BioRender.com.

## Supplementary information

Appendix

Table EV1

Peer Review File

Dataset EV1

Dataset EV2

Dataset EV5

Dataset EV3

Dataset EV6

Dataset EV4

Source data Fig. 1

Source data Fig. 2

Source data Fig. 3

Source data Fig. 4

Source data Fig. 5

## Data Availability

The Polysome profiling RNA-seq data generated in this manuscript have been deposited in the Gene Expression Omnibus (GEO) under accession code: GSE327671. The source data of this paper are collected in the following database record: biostudies:S-SCDT-10_1038-S44321-026-00499-1.
