# Supplementary material for: PRMT5-mediated intron retention triggers innate and adaptive immunity against cancer
Source: EMBO Mol Med. 2026 Aug 17;18(9):3677–703. doi: 10.1038/s44321-026-00499-1 (PMC13562731; doi:10.1038/s44321-026-00499-1)
Supplement: Supplementary file 1 [file 44321_2026_499_MOESM1_ESM.pdf]

Supplementary Materials and Figures

Interplay between PRMT5 and retained introns controls innate and adaptive immune responses against cancer cells

Wiktoria Blaszczak[1]*, Wojciech Barczak[1]*, Chuyue Zhang[1], Garret Rochford[1], Annalisa Nicastri[2], Claire Hutchings[3], Anastasia Samsonova[4], Alexander Kanapin[4], Nicola Ternette[2], Paul Klenerman[3,5] and Nicholas B La Thangue[1~]

[1] Laboratory of Cancer Biology, Department of Oncology, University of Oxford

Old Road Campus Research Building, Oxford, OX3 7DQ, United Kingdom

[2] The Jenner Institute, Nuffield Department of Medicine, University of Oxford, Oxford, OX3 7BN, United Kingdom

[3] Peter Medawar Building for Pathogen Research, University of Oxford, Oxford, OX1 3SY, United Kingdom

[4] Medical Science Division, University of Oxford, Old Road Campus Research Building, Oxford, OX3 7DQ, United Kingdom

[5] Translational Gastroenterology and Liver Unit, Nuffield Department of Medicine, Experimental Medicine, University of Oxford, Oxford, OX3 9DU, United Kingdom

* These Authors contributed equally to this paper

~ Corresponding Author: nick.lathangue@oncology.ox.ac.uk

Table of content:

Appendix Figure S1. Retained intron analysis in HCT116 cells.	Page 3
Appendix Figure S2. Retained intron analysis in HCT116 cells.	Page 6
Appendix Figure S3. Retained intron analysis in HCT116 cells.	Page 8
Appendix Figure S4. Retained intron analysis in CT26 cells.	Page 10
Appendix Figure S5. Immunopeptidomics on HCT116 cells.	Page 16
Appendix Figure S6. Immunopeptidomics of CT26 cells.	Page 22
Appendix Figure S7. Immunopeptidomics of CT26 cells	Page 24
Appendix Figure S8. Immunogenicity of ChAdOx1-PepMInt vaccine.	Page 26
Appendix Figure S9. Immunogenicity of ChAdOx1-PepMInt vaccine.	Page 28
Appendix Figure S10. Immunogenicity of ChAdOx1-PepMInt vaccine.	Page 30
Appendix Figure S11. Immunogenicity of the human RI-derived peptides.	Page 34
Appendix Figure S12. Level of RIs in human cancer.	Page 36

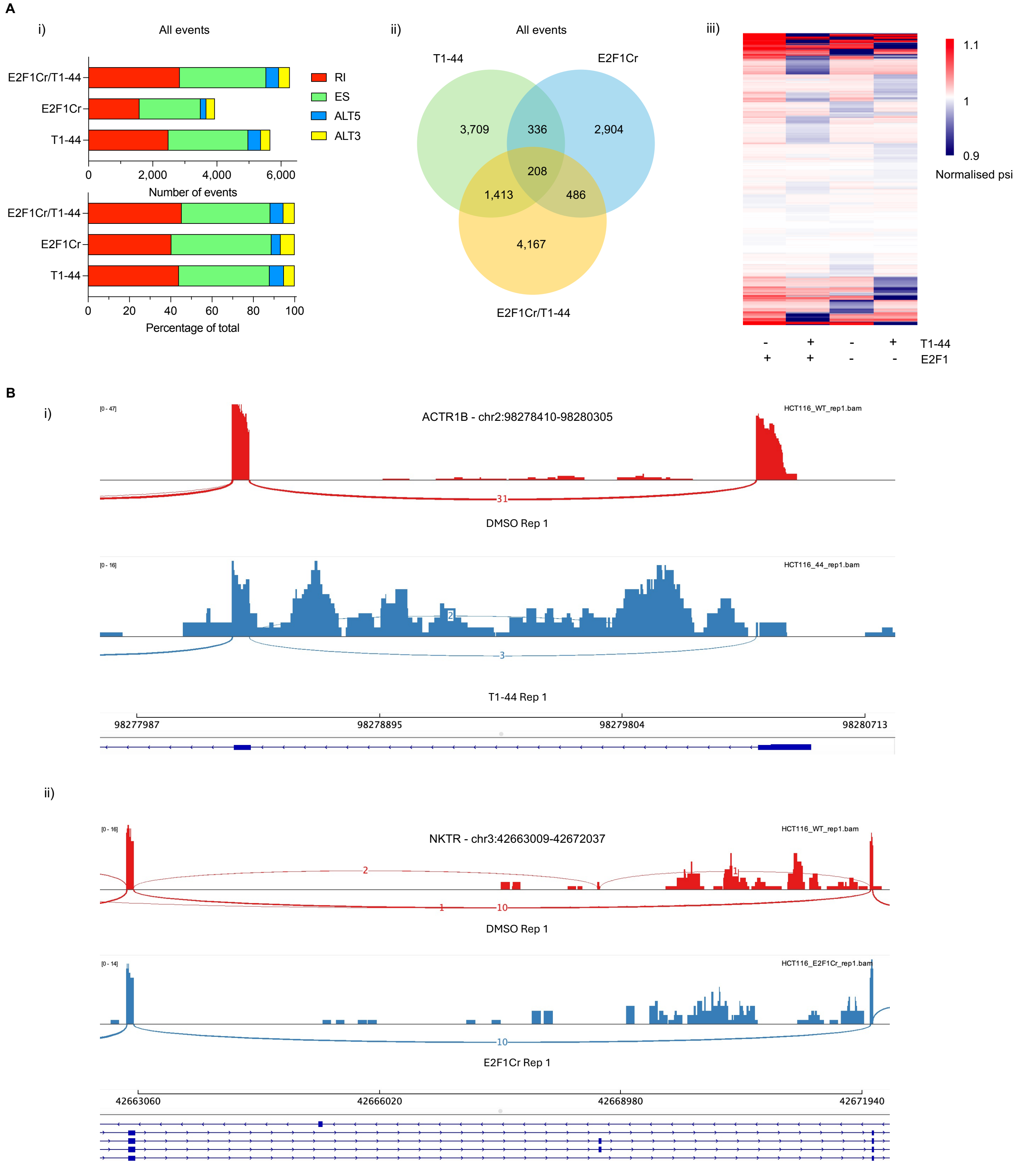
A
i)
All events
E2F1Cr/T1-44
E2F1Cr
T1-44
RI
ES
ALT5
ALT3
0
2,000
4,000
6,000
Number of events
0
20
40
60
80
100
Percentage of total
ii)
All events
T1-44
E2F1Cr
3,709
336
2,904
208
1,413
486
4,167
E2F1Cr/T1-44
iii)
1.1
1
0.9
Normalised psi
- + - + T1-44
+ + - - E2F1
B
i)
ACTR1B - chr2:98278410-98280305
HCT116_WT_rep1.bam
31
DMSO Rep 1
HCT116_44_rep1.bam
2
3
T1-44 Rep 1
98277987
98278895
98279804
98280713
ii)
NKTR - chr3:42663009-42672037
HCT116_WT_rep1.bam
2
1
10
DMSO Rep 1
HCT116_E2F1Cr_rep1.bam
10
E2F1Cr Rep 1
42663060
42666020
42668980
42671940

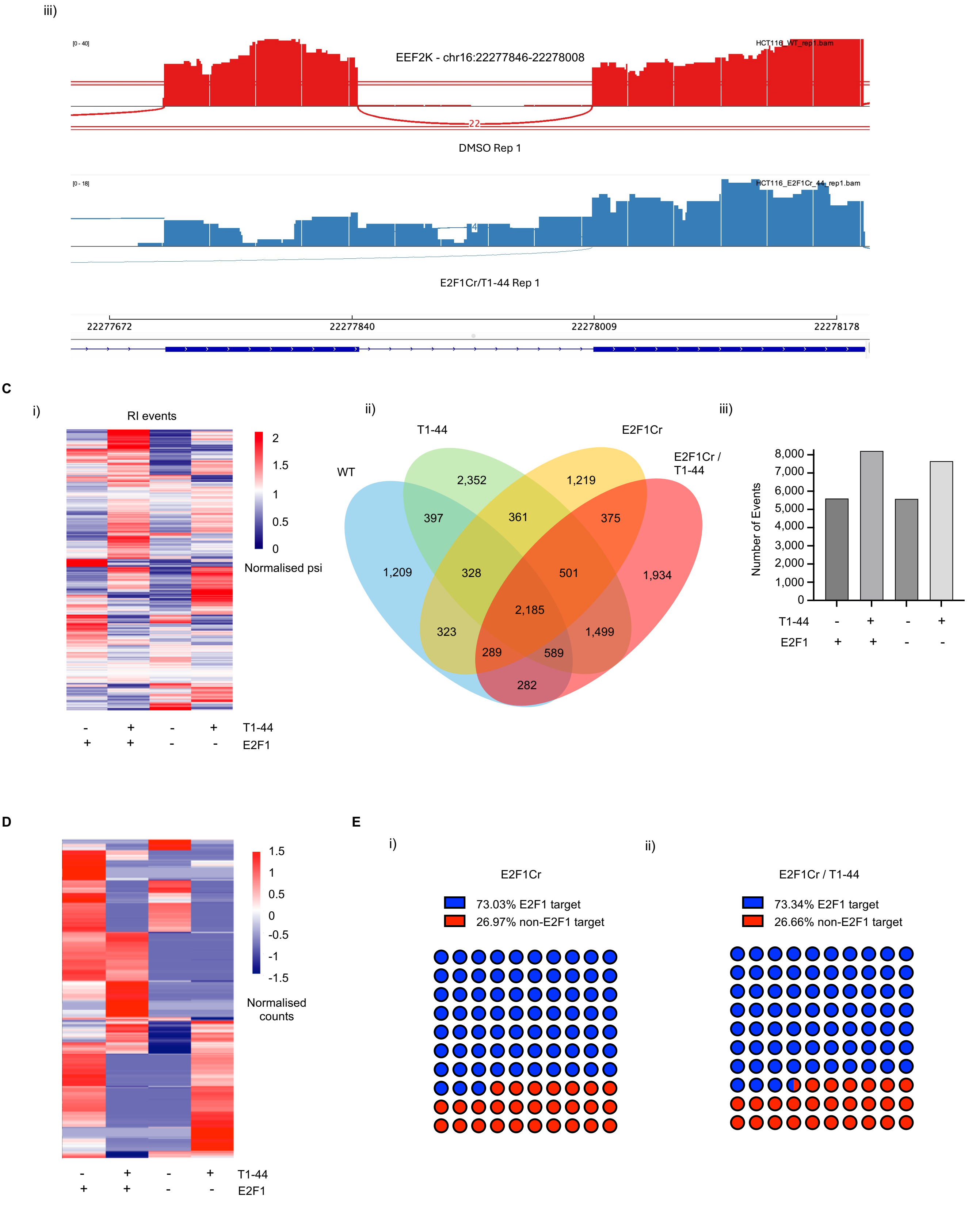
iii)
[0 - 40]
EEF2K - chr16:22277846-22278008
HCT116_WT_rep1.bam
22
DMSO Rep 1
[0 - 18]
HCT116_E2F1Cr_44_rep1.bam
E2F1Cr/T1-44 Rep 1
22277672
22277840
22278009
22278178
C
i)
RI events
2
1.5
1
0.5
0
Normalised psi
- + - + T1-44
+ + - - E2F1
ii)
T1-44
E2F1Cr
WT
E2F1Cr / T1-44
2,352
1,219
397
361
375
1,209
328
501
1,934
2,185
323
1,499
289
589
282
iii)
Number of Events
8,000
7,000
6,000
5,000
4,000
3,000
2,000
1,000
0
T1-44 - + - +
E2F1 + + - -
D
1.5
1
0.5
0
-0.5
-1
-1.5
Normalised counts
- + - + T1-44
+ + - - E2F1
E
i)
E2F1Cr
73.03% E2F1 target
26.97% non-E2F1 target
ii)
E2F1Cr / T1-44
73.34% E2F1 target
26.66% non-E2F1 target

Appendix Figure S1. Retained intron analysis in HCT116 cells.

A) Alternative splicing analysis using VAST tool algorithm on RNA-seq data from HCT116 WT E2F1 and E2F1Cr cells treated with T1-44 for 48 h. (i) The bar charts display the breakdown of statistically significant (p-value < 0.05) differential splicing events observed in each of the indicated treatments, as compared to WT E2F1 HCT116 cells treated with DMSO; results presented as number of events (top) and percentage of total (bottom). ES, skipped/cassette exon; RI, retained intron; ALT5, alternative 5′ splice site; ALT3, alternative 3′ splice; (ii) Overlap of differentially spliced RIs in different conditions; p-value < 0.05; (iii) Results presented as a heatmap of normalised psi values for all detected Alternative Splicing events; p-value < 0.05; Red – higher psi; Blue – lower psi.
B) Sashimi plots from representative biological replicates of T1-44–treated cells (i), E2F1 knockdown cells (E2F1Cr) (ii), and T1-44 treated E2F1 knockdown cells (E2F1Cr/T1-44) (iii), showing read coverage within intronic regions of selected genes.
C) RI analysis using the VAST tool algorithm on RNA-seq data from HCT116 WT E2F1 and E2F1Cr cells treated with T1-44 for 48 h. (i) Results presented as a heatmap for RI events with more than 10% psi in wild type condition; Red – higher psi, Blue – lower psi (please see Heatmap generation and normalisation section in Material and Methods); (ii) Overlap of RI events between different conditions; for each condition 10% psi cut-off was applied; (iii) Total number of RI events in each condition; for each condition 10% psi cut-off was applied.
D) Expression of RI transcripts according to the VAST tool algorithm. Results calculated by kallisto package are presented as a rlog-transformed normalized counts; average from three biological replicates.
E) Percentage of RI genes differentially regulated at a statistically significant level (VAST tool; p-value < 0.05) in E2F1Cr (i) and E2F1Cr/T1-44 (ii) treated conditions as compared to DMSO treated WT E2F1 HCT116 that score as potential E2F1 target genes.

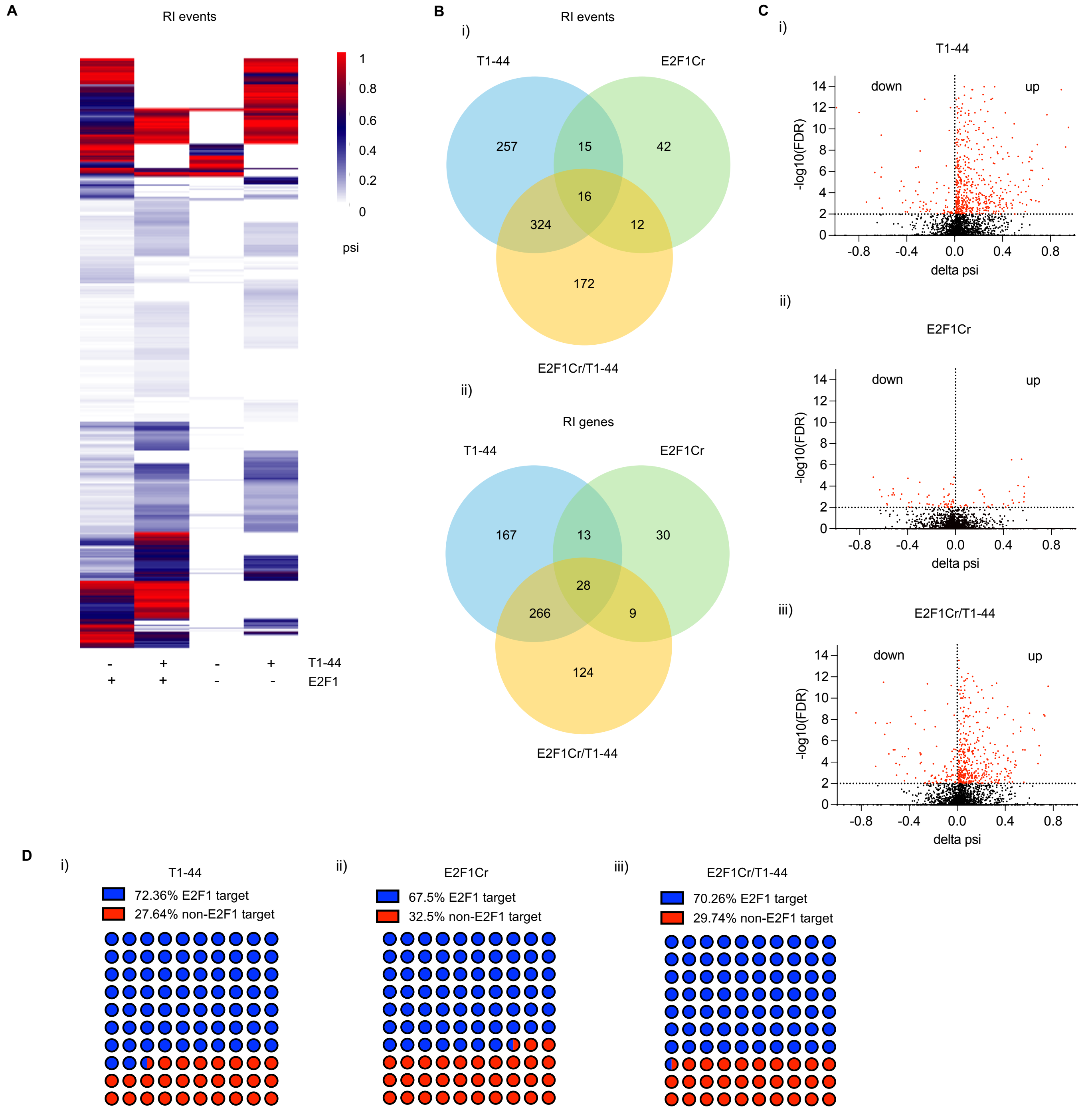
A
RI events
1
0.8
0.6
0.4
0.2
0
psi
- + - + T1-44
+ + - - E2F1
B
i)
RI events
T1-44
E2F1Cr
257
15
42
16
324
12
172
E2F1Cr/T1-44
ii)
RI genes
T1-44
E2F1Cr
167
13
30
28
266
9
124
E2F1Cr/T1-44
C
i)
T1-44
down
up
-log10(FDR)
delta psi
ii)
E2F1Cr
down
up
-log10(FDR)
delta psi
iii)
E2F1Cr/T1-44
down
up
-log10(FDR)
delta psi
D
i)
T1-44
72.36% E2F1 target
27.64% non-E2F1 target
ii)
E2F1Cr
67.5% E2F1 target
32.5% non-E2F1 target
iii)
E2F1Cr/T1-44
70.26% E2F1 target
29.74% non-E2F1 target

Appendix Figure S2. Retained intron analysis in HCT116 cells.

A) Differential splicing changes in RI transcripts (FDR < 0.01) in WT E2F1 and E2F1Cr cells treated with T1-44 for 48 h, with respect to WT E2F1 DMSO treatment, using the rMATS algorithm. Percentage Spliced In (psi) values were presented as a heatmap. Red colour represents higher psi, whilst blue colour represents lower one. White colour represents no splicing event. Splicing data were derived from three independent experiments.
B) (i) A Venn diagram showing the overlap between statistically significant differential RI events (FDR < 0.01) in each treatment, as compared to WT E2F1 HCT116 cells treated with DMSO; (ii) A Venn diagram showing the overlap between statistically significant differential RI genes (FDR < 0.01) in each treatment, as compared to WT E2F1 HCT116 cells treated with DMSO.
C) Volcano plots displaying values of delta psi and –log10 FDR values for differentially spliced RI events identified in T1-44 (i), E2F1Cr (ii), and E2F1Cr/T1-44 (iii) treated cells, as compared to WT E2F1 HCT116 cells treated with DMSO. Red colour represents events significantly changed. Black colour represents genes that fell below the statistical cut-off applied (FDR < 0.01).
D) Percentage of RI genes differentially regulated at a statistically significant level (FDR < 0.01) in T1-44 (i), E2F1Cr (ii), and E2F1Cr/T1-44 (iii) treated cells, as compared to WT E2F1 HCT116 cells treated with DMSO in HCT116 cells that score as potential E2F1 target genes.

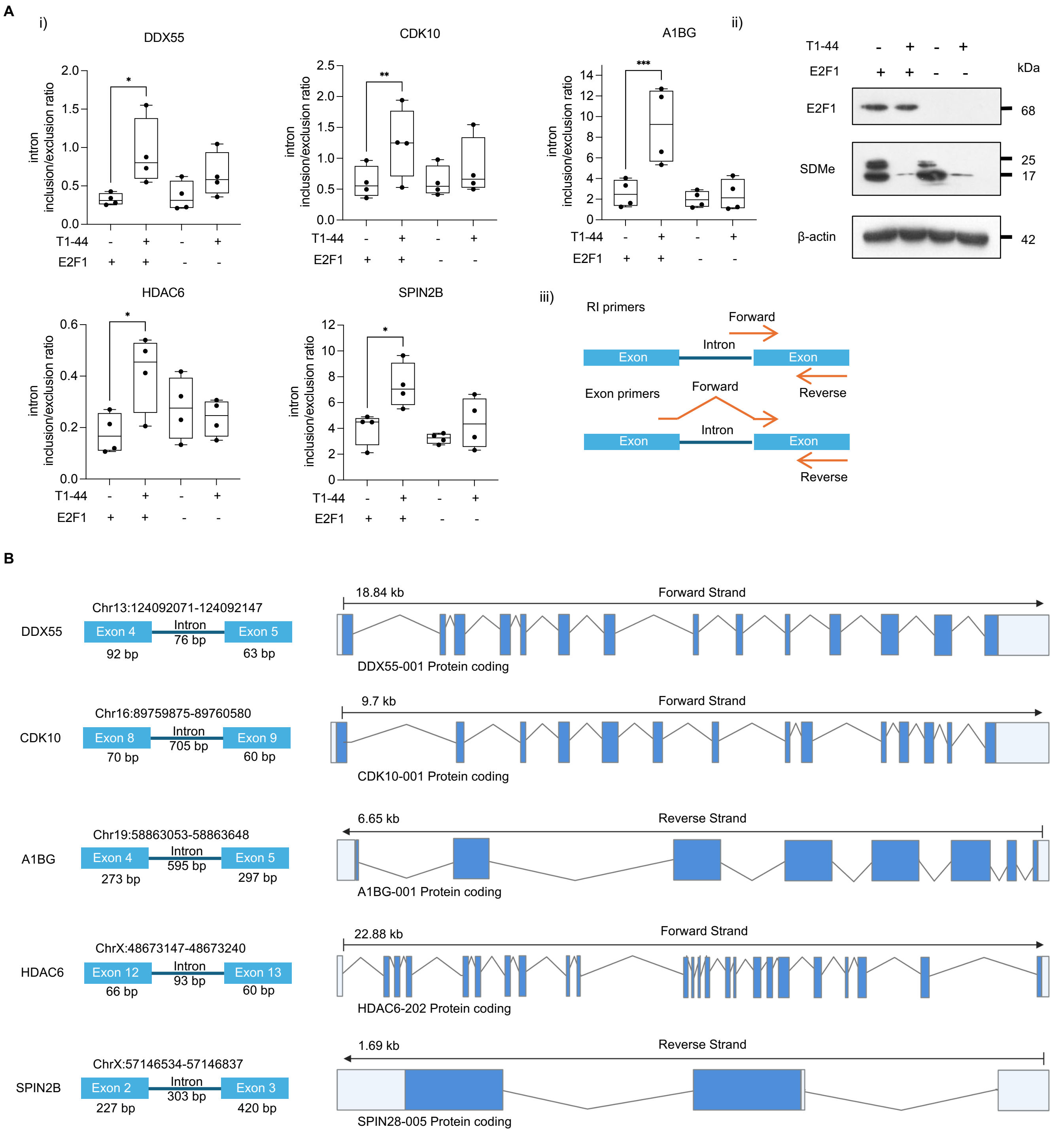
A
i)
DDX55
CDK10
A1BG
HDAC6
SPIN2B
intron inclusion/exclusion ratio
T1-44
E2F1
ii)
T1-44 - + - +
E2F1 + + - -
kDa
E2F1
68
SDMe
25
17
β-actin
42
iii)
RI primers
Forward
Intron
Exon
Exon primers
Reverse
B
DDX55
Chr13:124092071-124092147
Exon 4
Intron
76 bp
Exon 5
92 bp
63 bp
18.84 kb
Forward Strand
DDX55-001 Protein coding
CDK10
Chr16:89759875-89760580
Exon 8
Intron
705 bp
Exon 9
70 bp
60 bp
9.7 kb
Forward Strand
CDK10-001 Protein coding
A1BG
Chr19:58863053-58863648
Exon 4
Intron
595 bp
Exon 5
273 bp
297 bp
6.65 kb
Reverse Strand
A1BG-001 Protein coding
HDAC6
ChrX:48673147-48673240
Exon 12
Intron
93 bp
Exon 13
66 bp
60 bp
22.88 kb
Forward Strand
HDAC6-202 Protein coding
SPIN2B
ChrX:57146534-57146837
Exon 2
Intron
303 bp
Exon 3
227 bp
420 bp
1.69 kb
Reverse Strand
SPIN28-005 Protein coding

Appendix Figure S3. Retained intron analysis in HCT116 cells.

A) (i) WT E2F1 and E2F1 Cr HCT116 cells treated for 48 h with 1 μM T1-44 or DMSO as indicated. A qPCR was performed to measure the intron inclusion/exclusion ratios in DDX55, CDK10, A1BG, HDAC6, and SPIN2B RNA transcripts from the cells. The results show the mean inclusion/exclusion ratio, with individual data points, presented as Box and whisker plots. Significance was calculated by one-way ANOVA, * p-value <0.05, ** p-value <0.01, *** p-value <0.001; n=4, (ii) A representative immunoblot is included to display input protein levels of E2F1 and symmetric dimethylation (SDMe). Actin was used as a loading control; (iii) schema of the intron and exon primer design.

B) Organisation of the genes containing the RIs measured in A. The outline, size, and flanking exons are presented (left panel). In addition, the corresponding full transcript, size, and its orientation is indicated. Boxes represent exons; dark blue – coding sequence, light blue – untranslated regions; lines between boxes represent intronic sequences (right panel).

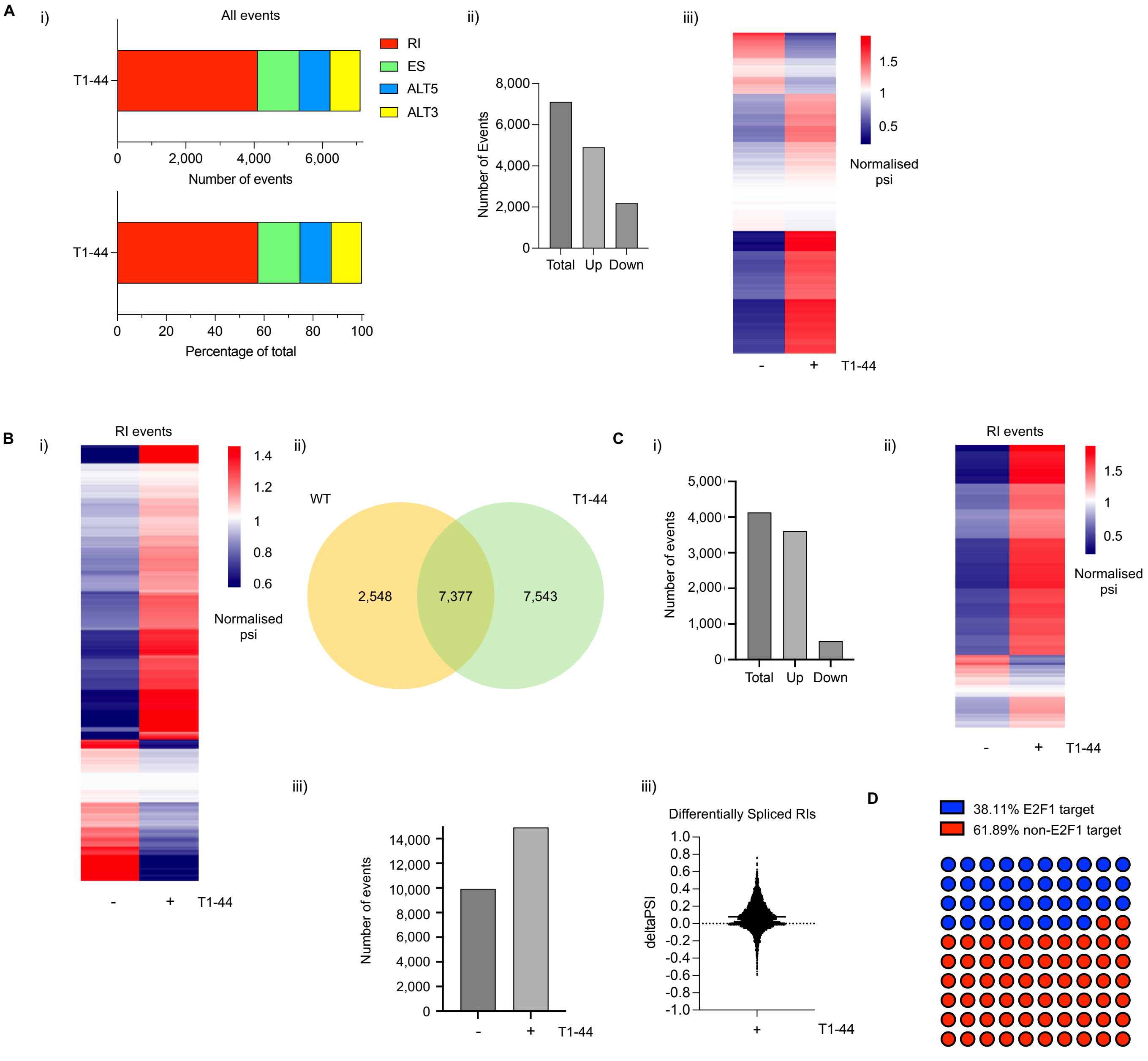
A
i)
All events
T1-44
RI
ES
ALT5
ALT3
Number of events
Percentage of total
ii)
Number of Events
Total
Up
Down
iii)
Normalised psi
T1-44
B
RI events
WT
2,548
7,377
7,543
Number of events
Differentially Spliced RIs
deltaPSI
C
D
38.11% E2F1 target
61.89% non-E2F1 target

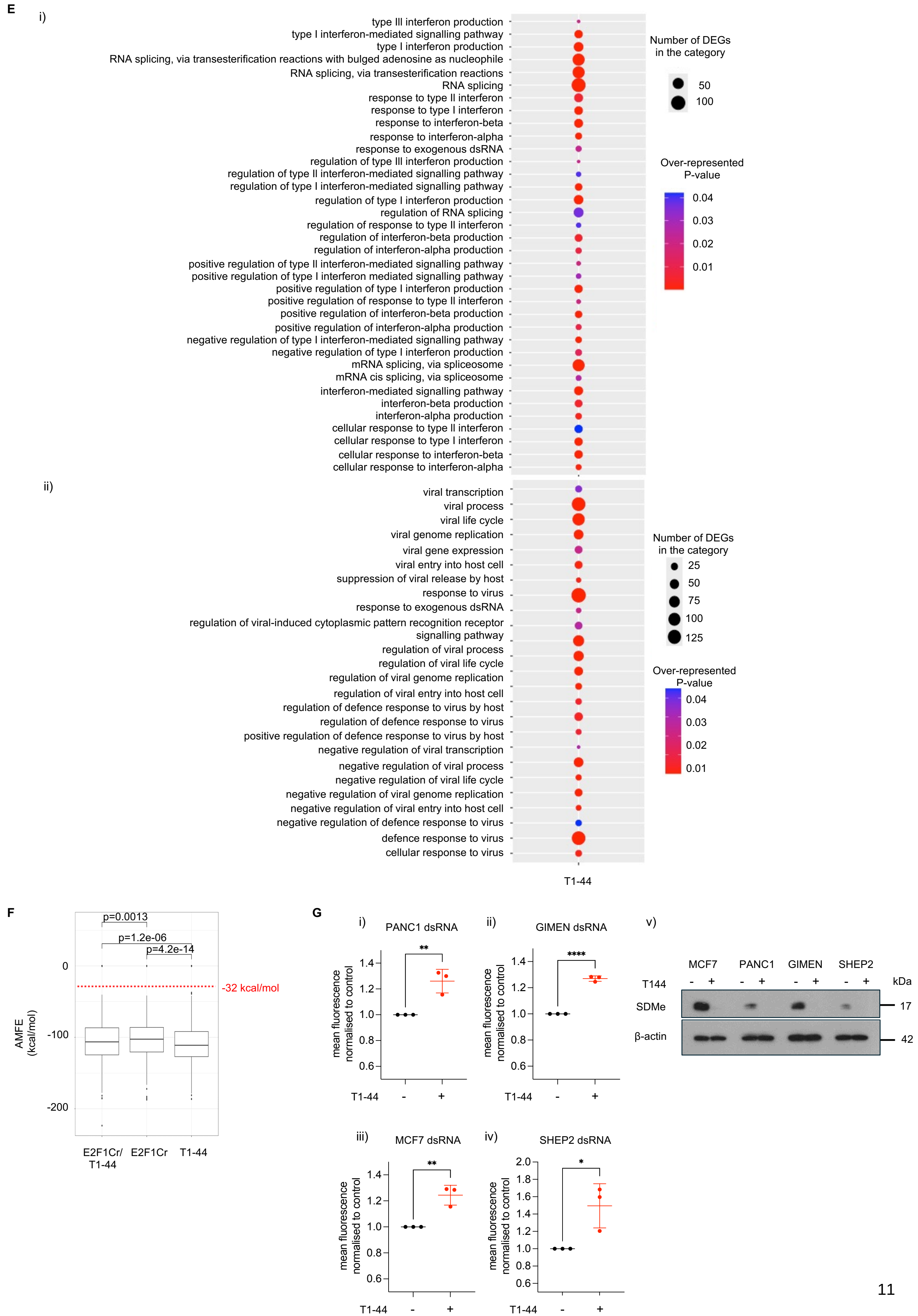
E
i)
type III interferon production
type I interferon-mediated signalling pathway
type I interferon production
RNA splicing, via transesterification reactions with bulged adenosine as nucleophile
RNA splicing, via transesterification reactions
RNA splicing
response to type II interferon
response to type I interferon
response to interferon-beta
response to interferon-alpha
response to exogenous dsRNA
regulation of type III interferon production
regulation of type II interferon-mediated signalling pathway
regulation of type I interferon-mediated signalling pathway
regulation of type I interferon production
regulation of RNA splicing
regulation of response to type II interferon
regulation of interferon-beta production
regulation of interferon-alpha production
positive regulation of type II interferon-mediated signalling pathway
positive regulation of type I interferon mediated signalling pathway
positive regulation of type I interferon production
positive regulation of response to type II interferon
positive regulation of interferon-beta production
positive regulation of interferon-alpha production
negative regulation of type I interferon-mediated signalling pathway
negative regulation of type I interferon production
mRNA splicing, via spliceosome
mRNA cis splicing, via spliceosome
interferon-mediated signalling pathway
interferon-beta production
interferon-alpha production
cellular response to type II interferon
cellular response to type I interferon
cellular response to interferon-beta
cellular response to interferon-alpha
Number of DEGs in the category
50
100
Over-represented P-value
0.04
0.03
0.02
0.01
ii)
viral transcription
viral process
viral life cycle
viral genome replication
viral gene expression
viral entry into host cell
suppression of viral release by host
response to virus
response to exogenous dsRNA
regulation of viral-induced cytoplasmic pattern recognition receptor signalling pathway
regulation of viral process
regulation of viral life cycle
regulation of viral genome replication
regulation of viral entry into host cell
regulation of defence response to virus by host
regulation of defence response to virus
positive regulation of defence response to virus by host
negative regulation of viral transcription
negative regulation of viral process
negative regulation of viral life cycle
negative regulation of viral genome replication
negative regulation of viral entry into host cell
negative regulation of defence response to virus
defence response to virus
cellular response to virus
Number of DEGs in the category
25
50
75
100
125
Over-represented P-value
0.04
0.03
0.02
0.01
T1-44
F
p=0.0013
p=1.2e-06
p=4.2e-14
0
-100
-200
-32 kcal/mol
AMFE (kcal/mol)
E2F1Cr/ T1-44
E2F1Cr
T1-44
G
i) PANC1 dsRNA
ii) GIMEN dsRNA
iii) MCF7 dsRNA
iv) SHEP2 dsRNA
mean fluorescence normalised to control
T1-44
-
+
**
****
**
*
v)
MCF7
PANC1
GIMEN
SHEP2
T144
kDa
SDMe
β-actin
17
42

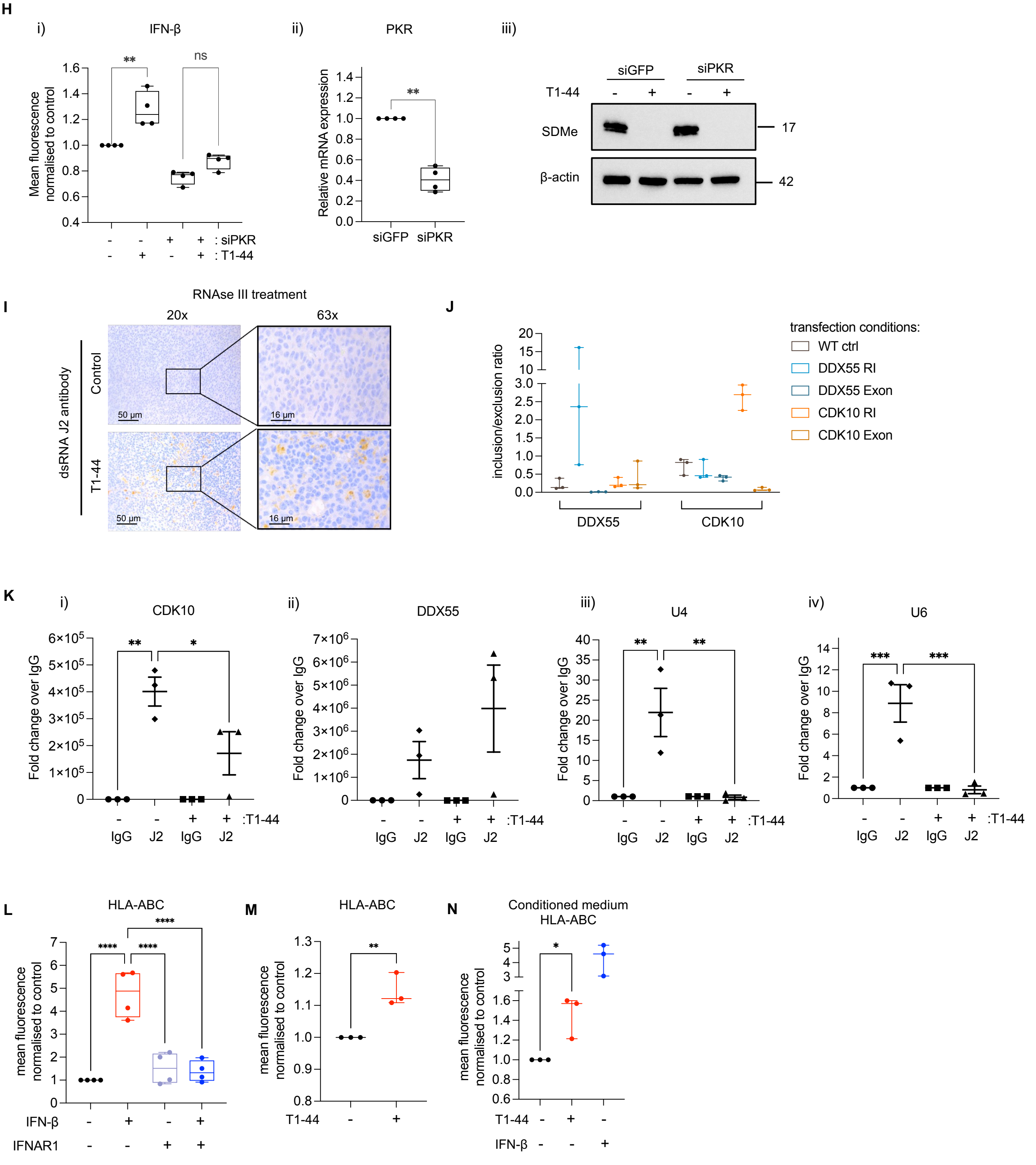

H
i)
IFN-β
Mean fluorescence normalised to control
1.6
1.4
1.2
1.0
0.8
0.6
0.4
**
ns
- - + + : siPKR
- + - + : T1-44
ii)
PKR
Relative mRNA expression
1.4
1.2
1.0
0.8
0.6
0.4
0.2
0.0
**
siGFP siPKR
iii)
siGFP siPKR
T1-44 - + - +
SDMe
17
β-actin
42
I
RNAse III treatment
20x 63x
dsRNA J2 antibody
Control
T1-44
50 µm
16 µm
J
inclusion/exclusion ratio
20
15
10
3.0
2.5
2.0
1.5
1.0
0.5
0.0
DDX55 CDK10
transfection conditions:
WT ctrl
DDX55 RI
DDX55 Exon
CDK10 RI
CDK10 Exon
K
i)
CDK10
Fold change over IgG
6×10^5
5×10^5
4×10^5
3×10^5
2×10^5
1×10^5
0
**
*
- - + + :T1-44
IgG J2 IgG J2
ii)
DDX55
Fold change over IgG
7×10^6
6×10^6
5×10^6
4×10^6
3×10^6
2×10^6
1×10^6
0
- - + + :T1-44
IgG J2 IgG J2
iii)
U4
Fold change over IgG
40
35
30
25
20
15
10
5
0
**
**
- - + + :T1-44
IgG J2 IgG J2
iv)
U6
Fold change over IgG
14
12
10
8
6
4
2
0
***
***
- - + + :T1-44
IgG J2 IgG J2
L
HLA-ABC
mean fluorescence normalised to control
7
6
5
4
3
2
1
0
****
****
****
IFN-β - + - +
IFNAR1 - - + +
M
HLA-ABC
mean fluorescence normalised to control
1.3
1.2
1.1
1.0
0.9
0.8
**
T1-44 - +
N
Conditioned medium
HLA-ABC
mean fluorescence normalised to control
5
4
3
1.6
1.4
1.2
1.0
0.8
0.6
*
T1-44 - + -
IFN-β - - +

Appendix Figure S4. Retained intron analysis in CT26 cells.

A) Alternative Splicing analysis using the VAST tool algorithm of RNA-seq data from CT26 cells treated with T1-44 for 72 h. (i) The bar charts display the breakdown of statistically significant (p-value < 0.05) differential splicing events observed in each of the indicated treatments, as compared to CT26 cells treated with DMSO; results presented as number of events (top) and percentage of total (bottom). ES, skipped/cassette exon; RI, retained intron; ALT5, alternative 5′ splice site; ALT3, alternative 3′ splice; Differential analysis of Alternative Splicing events by VAST tool of CT26 cells treated with T1-44 for 72 h. Results of the differentially upregulated (Up) and downregulated (Down) upon T1-44 splicing events presented as a graph (ii) and heatmap (iii); p-value < 0.05; Red represents higher psi, blue lower psi, and white no difference between conditions.

B) RI analysis using the VAST tool algorithm of RNA-seq data from CT26 cells treated with T1-44 for 72 h. (i) Results presented as a heatmap for RI events with more than 10% psi in wild type condition; Red represents higher psi, blue lower psi, and white no difference between conditions (please see Heatmap generation and normalisation section in Material and Methods); (ii) Overlap of RI events between different conditions; for each condition 10% psi cut-off was applied; (iii) Total number of RI events in each condition; for each condition 10% psi cut-off was applied.

C) Differential analysis of RI events by VAST tool of CT26 cells treated with T1-44 for 72 h. Results of the differentially upregulated (Up) and downregulated (Down) splicing RI events presented as a graph (i) and heatmap (ii); p-value < 0.05; Red represents higher psi, blue lower psi, and white no difference between conditions; (iii) Differential analysis of RIs using VAST tool on RNA-seq datasets derived from CT26 cells treated with T1-44. Results of the differentially upregulated and downregulated RI events presented as a delta PSI plot.

D) Percentage of RI genes differentially regulated at a statistically significant level (VAST tool; p-value < 0.05) in T1-44 treated cells as compared to CT26 cells treated with DMSO that score as potential E2F1 target genes.

E) Gene ontology analyses (i, ii) were performed using goseq R package on lists of differentially expressed genes (p-value < 0.05). GO biological processes and p-value < 0.05 cut-off were used for pathway enrichment.

F) The prevalence estimation and stability prediction of the RI double-stranded structures in HCT116 E2F1 WT and E2F1Cr cells treated with DMSO or T1-44 identified in VAST tool analysis using AMFE (adjusted MFE) method. Results are presented as Box and Whiskers plot. Box and whiskers are defined as first quartile, median, and third quartile. P values are also displayed. Red line shows AFME value for random sequences from Trotta et al (2014).

G) Quantitation of dsRNA intracellular staining (measured by flow cytometry) in PANC1 (Pancreatic Adenocarcinoma) (i), GIMEN (Neuroblastoma) (ii), MCF7 (Breast cancer) (iii), and SHEP2 (Neuroblastoma) (iv) cells treated with DMSO or T1-44. Mean fluorescence was normalised to DMSO treated WT cells (control); n=3; * p< 0.05, ** p< 0.01, **** p<0.0001, Student's t test. (v) A representative immunoblot showing T1-44 activity by SDMe mark is also shown.

H) Quantitation of IFN-β (i) intracellular staining (measured by flow cytometry) in HCT116 WT and siPKR knockdown cells treated with 1 uM T1-44 for 48h. Mean fluorescence was normalised to DMSO treated WT cells, n=4; (ii) Efficiency of siPKR treatment measured by qPCR has been presented as well, n=4; (iii) A representative immunoblot showing T1-44 activity by SDMe mark is also shown.

I) An example of immunohistochemical staining of dsRNA on Colon26 tumour tissue in RNAse III treated slides as an additional negative control; Original magnification: 20x, scale bar, 50 μm; and 63x; scale bar, 16 μm.

J) qPCR-measured inclusion/exclusion ratio of RI and Exon transcripts confirming transfection of DDX55 and CDK10 plasmids, compared to non-transfected cells. Different biological replicates presented as a Box and Whiskers plot. Box and whiskers are defined as minimum, first quartile, median, third quartile, and maximum of data.

K) Detection of dsRNA by J2 immunoprecipitation (dsRNA RIP). Double-stranded RNA (dsRNA) was immunoprecipitated from HCT116 cell lysates treated with T1-44 using the J2 monoclonal antibody. Total input lysate, J2 immunoprecipitated

material (J2), and control IgG IP for CDK10 (i) and DDX55 (ii) introns were analysed by qPCR; U4 (iii) and U6 (iv) transcripts detection was used as a positive control. Results and presented as enrichment of dsRNA species in the J2 IP over with IgG control. * $p < 0.05$, ** $p < 0.01$, *** $p < 0.001$, one-way ANOVA; n=3.

L) HLA-ABC flow cytometry analysis at the HCT116 cells surface after treatment with IFN-β, IFNAR1 antibody, and IFN-β/IFNAR1; results are presented as normalised mean fluorescence values; n=4, **** p value < 0.0001, one-way ANOVA.

M) HLA-ABC flow cytometry analysis at the HCT116 cells surface after 48h treatment with 1 μM T1-44; results are presented as normalised mean fluorescence values; n=3; ** p value < 0.01, Student's t test.

N) HLA-ABC flow cytometry analysis at the HCT116 cell surface after 48h treatment with conditioned medium after pre-treatment with 1 μM T1-44. IFN-β treatment was used as positive control. Results are presented as normalised mean fluorescence values; n=3; * p value < 0.05, Student's t test.

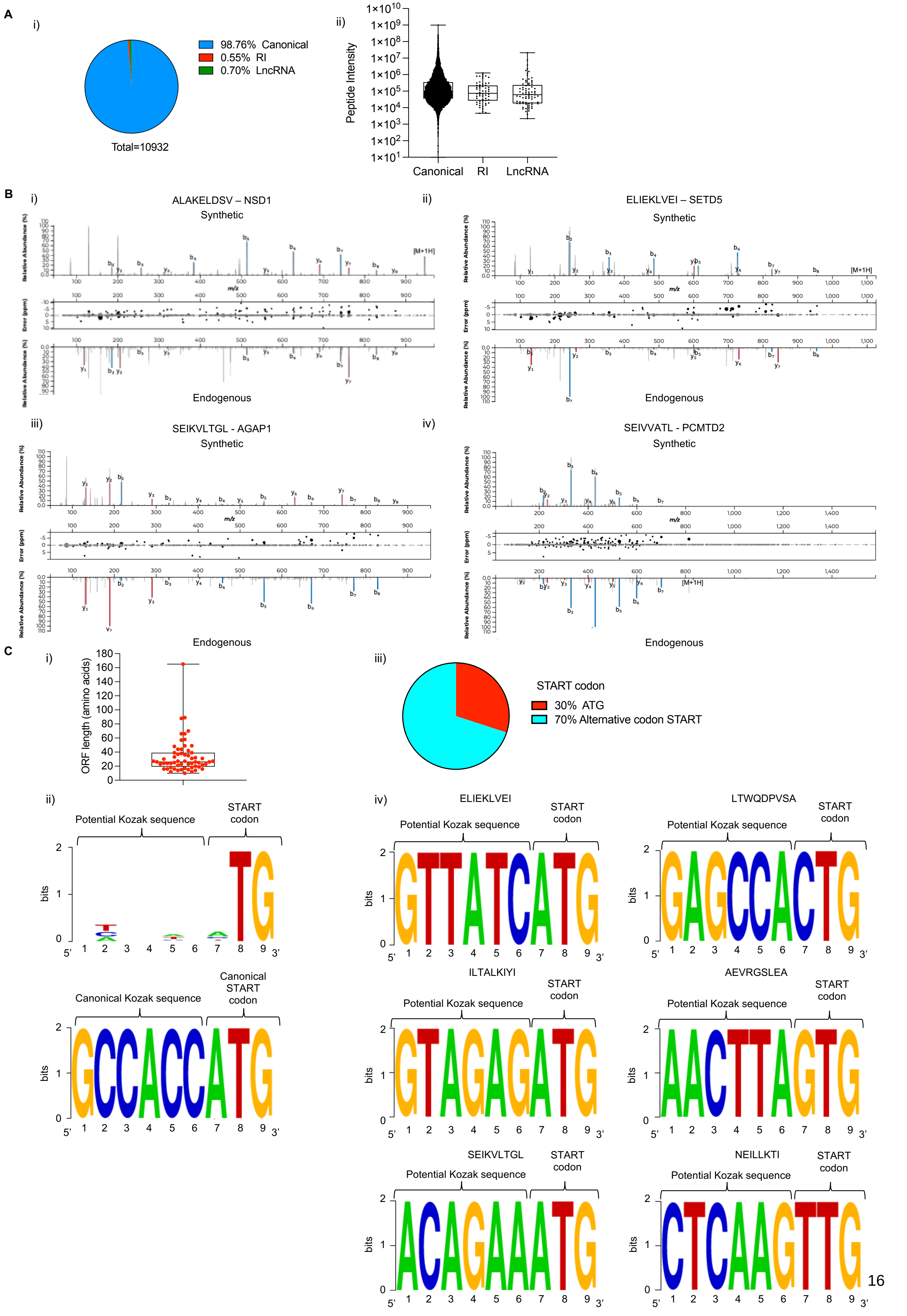
A
i)
98.76% Canonical
0.55% RI
0.70% LncRNA
Total=10932
ii)
Peptide Intensity
Canonical
RI
LncRNA
B
i)
ALAKELDSV – NSD1
Synthetic
Endogenous
ii)
ELIEKLVEI – SETD5
Synthetic
Endogenous
iii)
SEIKVLTGL - AGAP1
Synthetic
Endogenous
iv)
SEIVVATL - PCMTD2
Synthetic
Endogenous
C
i)
ORF length (amino acids)
iii)
START codon
30% ATG
70% Alternative codon START
ii)
Potential Kozak sequence
START codon
Canonical Kozak sequence
Canonical START codon
iv)
ELIEKLVEI
LTWQDPVSA
ILTALKIYI
AEVRGSLEA
SEIKVLTGL
NEILLKTI

D

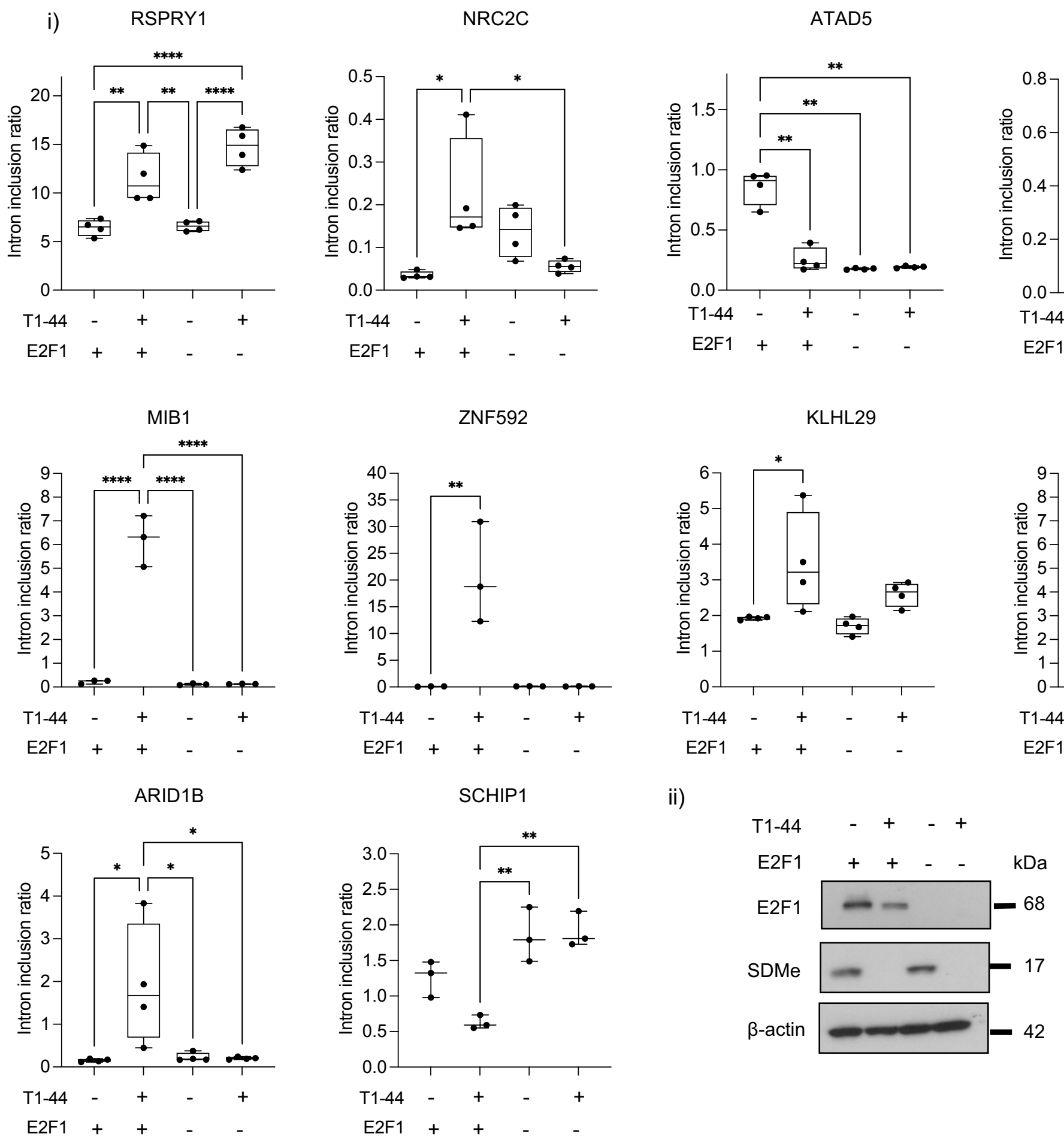
i)
RSPRY1
NRC2C
ATAD5
CDKAL1
SETD5
MIB1
ZNF592
KLHL29
FNDC3B
FAM188B
ARID1B
SCHIP1
Intron inclusion ratio
T1-44
E2F1
ii)
T1-44
E2F1
kDa
E2F1
68
SDMe
17
β-actin
42

E

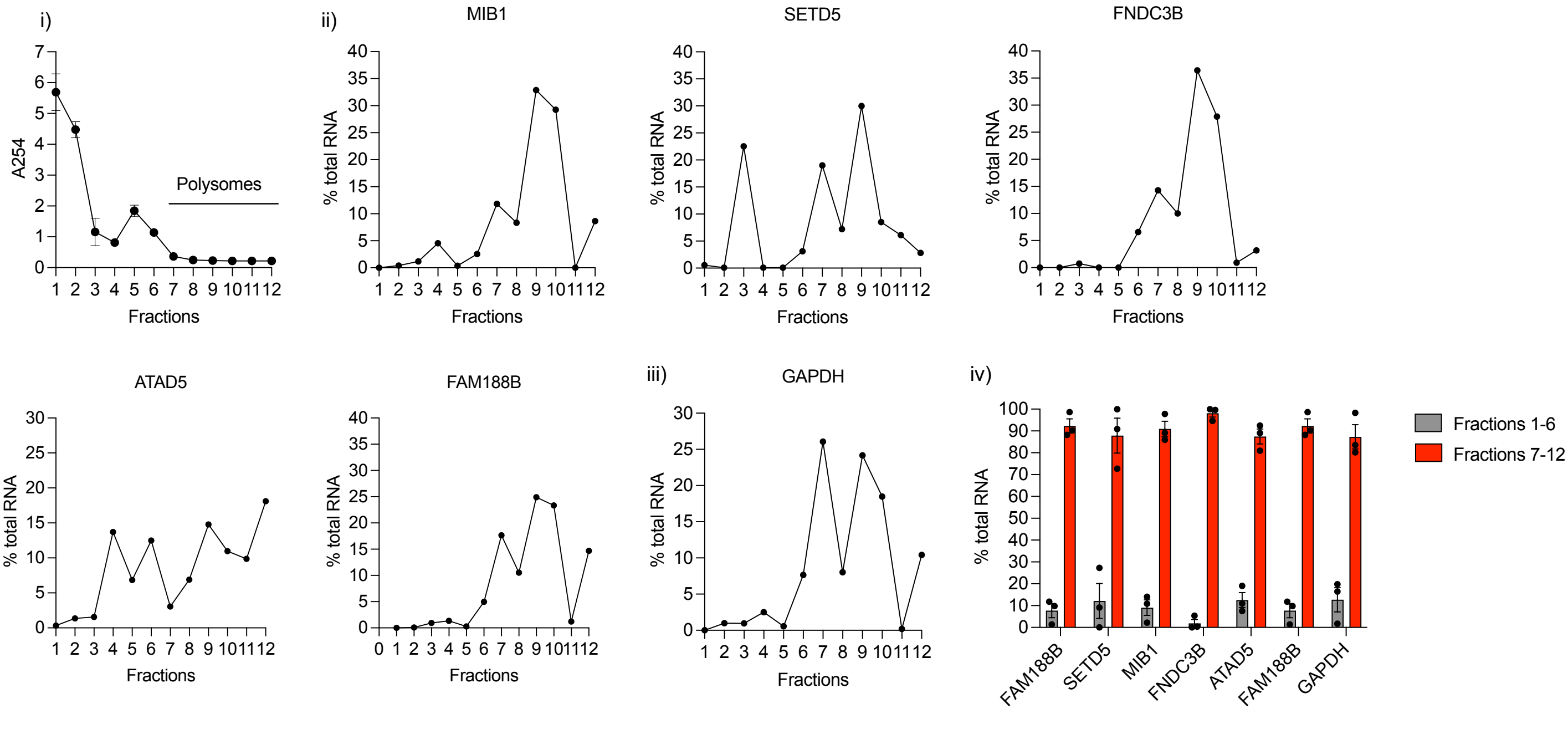
i)
A254
Polysomes
Fractions
ii)
MIB1
SETD5
FNDC3B
ATAD5
FAM188B
% total RNA
Fractions
iii)
GAPDH
iv)
Fractions 1-6
Fractions 7-12
FAM188B
SETD5
MIB1
FNDC3B
ATAD5
FAM188B
GAPDH

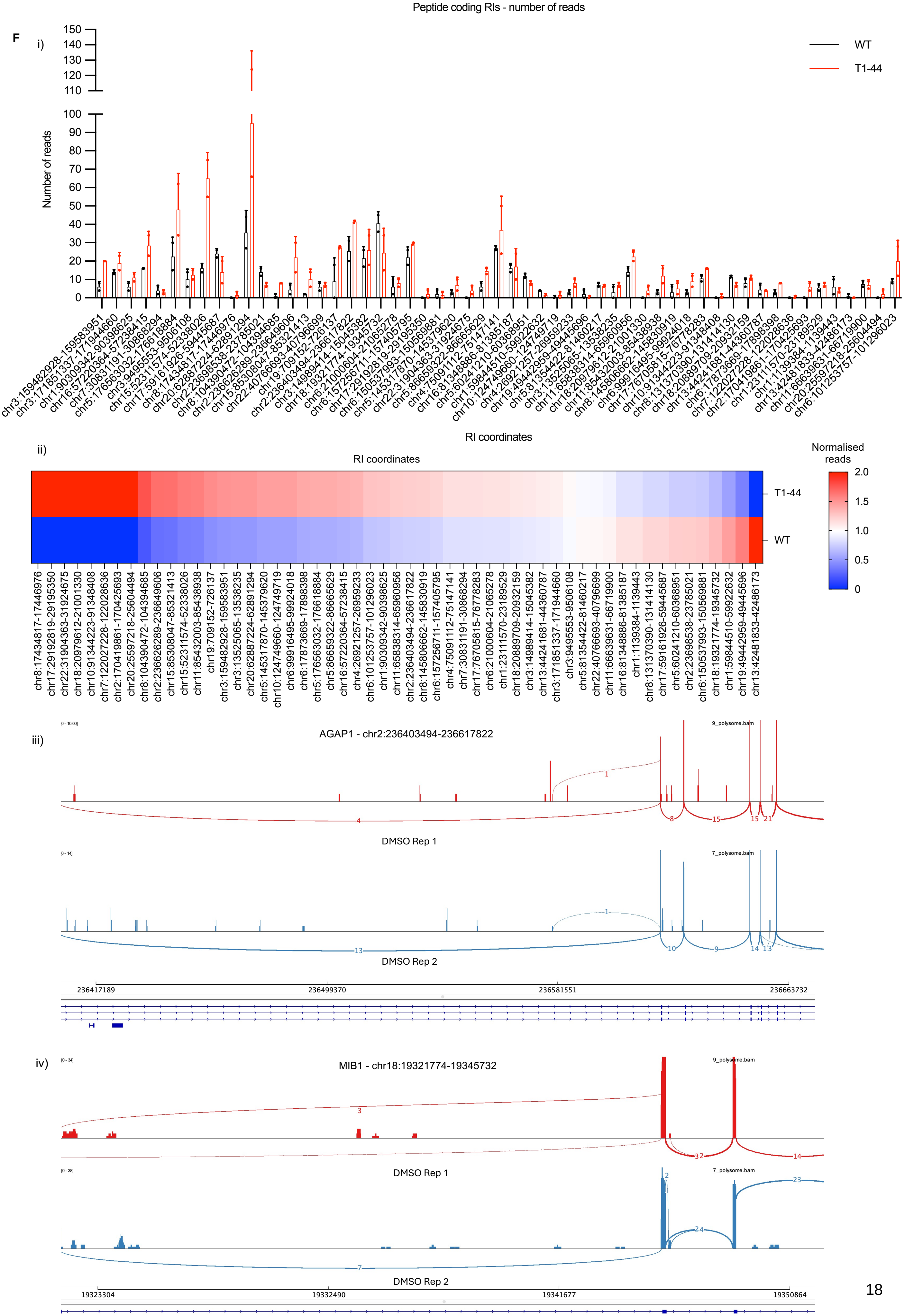
Peptide coding RIs - number of reads
F
i)
ii)
iii)
iv)
WT
T1-44
Number of reads
RI coordinates
Normalised reads
AGAP1 - chr2:236403494-236617822
DMSO Rep 1
DMSO Rep 2
MIB1 - chr18:19321774-19345732
DMSO Rep 1
DMSO Rep 2

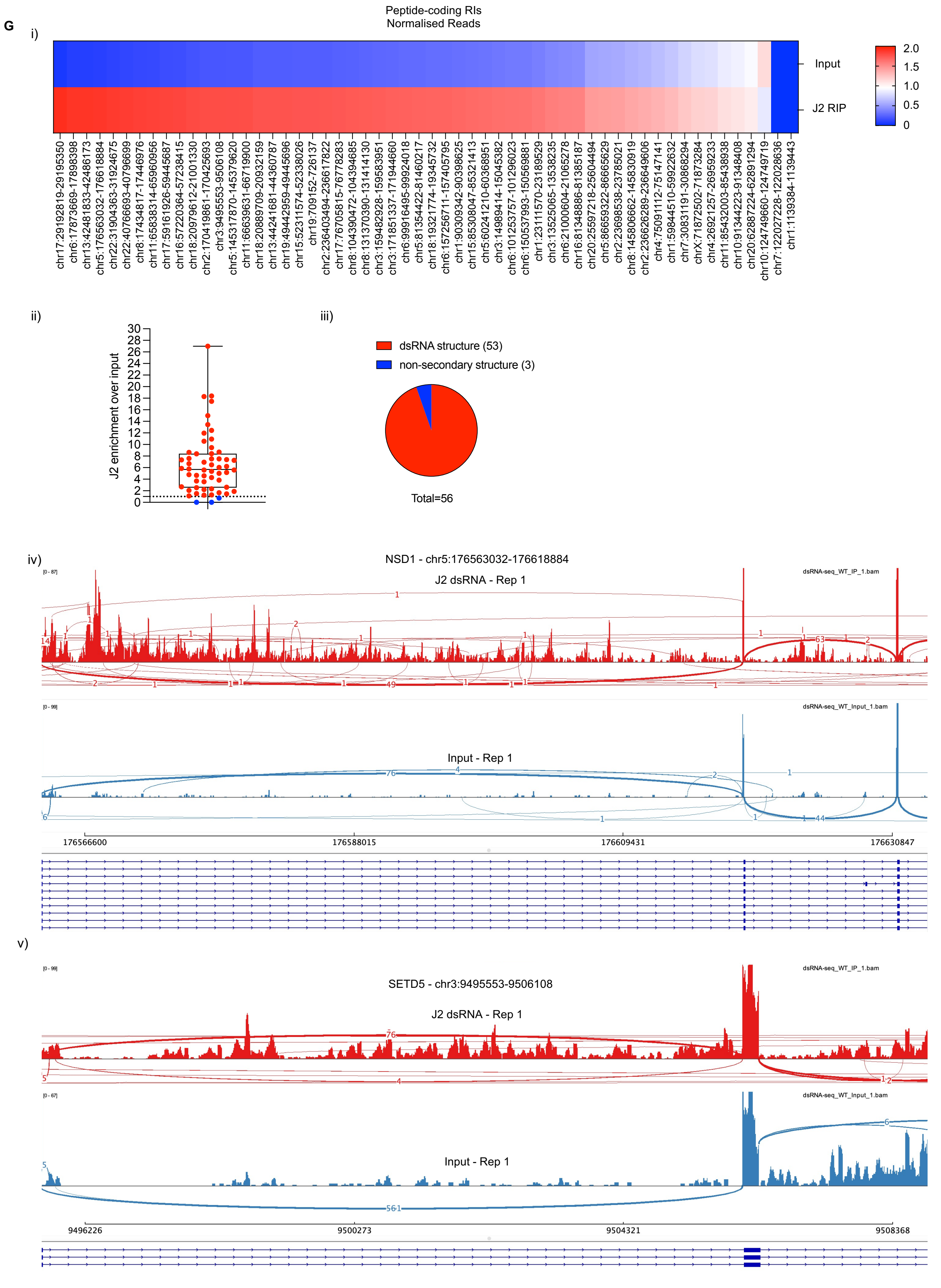

G
i)
Peptide-coding RIs
Normalised Reads
Input
J2 RIP
2.0
1.5
1.0
0.5
0
chr17:29192819-29195350
chr6:17873669-17898398
chr13:42481833-42486173
chr5:176563032-176618884
chr22:31904363-31924675
chr22:40766693-40796699
chr8:17434817-17446976
chr11:65838314-65960956
chr17:59161926-59445687
chr16:57220364-57238415
chr18:20979612-21001330
chr2:170419861-170425693
chr3:9495553-9506108
chr5:145317870-145379620
chr11:66639631-66719900
chr18:20889709-20932159
chr13:44241681-44360787
chr19:49442959-49445696
chr15:52311574-52338026
chr19:709152-726137
chr2:236403494-236617822
chr17:76705815-76778283
chr8:104390472-104394685
chr8:131370390-131414130
chr3:159482928-159583951
chr3:171851337-171944660
chr6:99916495-99924018
chr5:81354422-81460217
chr18:19321774-19345732
chr6:157256711-157405795
chr1:90309342-90398625
chr15:85308047-85321413
chr5:60241210-60368951
chr3:14989414-15045382
chr6:101253757-101296023
chr6:150537993-150569881
chr1:23111570-23189529
chr3:13525065-13538235
chr6:21000604-21065278
chr16:81348886-81385187
chr20:25597218-25604494
chr5:86659322-86665629
chr2:23698538-23785021
chr8:145806662-145830919
chr2:236626289-236649606
chr4:75091112-75147141
chr1:59844510-59922632
chr7:30831191-30868294
chrX:71872502-71873284
chr4:26921257-26959233
chr11:85432003-85438938
chr10:91344223-91348408
chr20:62887224-62891294
chr10:124749660-124749719
chr7:122027228-122028636
chr1:1139384-1139443
ii)
J2 enrichment over input
iii)
dsRNA structure (53)
non-secondary structure (3)
Total=56
iv)
NSD1 - chr5:176563032-176618884
J2 dsRNA - Rep 1
dsRNA-seq_WT_IP_1.bam
Input - Rep 1
dsRNA-seq_WT_Input_1.bam
176566600
176588015
176609431
176630847
v)
SETD5 - chr3:9495553-9506108
J2 dsRNA - Rep 1
dsRNA-seq_WT_IP_1.bam
Input - Rep 1
dsRNA-seq_WT_Input_1.bam
9496226
9500273
9504321
9508368

Appendix Figure S5. Immunopeptidomics on HCT116 cells.

A) (i) Pie charts showing the distribution of identified peptides across different categories in HCT116. Results presented as percentage of total unique peptides assigned to each category; (ii) Distribution of MS signal intensity for each peptide across different categories in HCT116 cells. Data are derived from two independent biological replicates analysed by LC–MS/MS.

B) Synthetic peptides corresponding to MHC class I bound peptides identified from the HCT116 immunopeptidomics analysis were synthesised and analysed by mass spectrometry to confirm the identifications made from the immunopeptidomics analysis. Examples are shown of the peptides encoded by RIs, ALAKELDSV (i), ELIEKLVEI (ii), SEIKVLTGL (iii), and SEIVVATL (iv). The bottom mass spectrogram represents the peptide profile derived from the endogenous HCT116 immunopeptidomics analysis, whilst the upper spectra represent the ion profile from the synthetic peptide.

C) (i) Summary of open reading frame length (ORFs) containing the peptide sequence and plotted as Box and whiskers. (ii) A sequence logo demonstrating the nucleotide conservation around the translation initiation sequences of all potential ORFs (with identified START codon) from all human RIs giving rise to MHC class I associated peptides (top) compared to the classical vertebrate consensus, displayed at the bottom. (iii) Identified START codons across the RI ORFs; (iv) Logo demonstrating the nucleotide sequence around the translation initiation site of potential ORFs (with identified START codon) from RIs giving rise to MHC class I associated peptides.

D) WT E2F1 HCT116 and E2F1Cr cells were treated for 48 h with 1 μM T1-44 or DMSO as indicated. RNA was isolated and used in qRT-PCR analysis with primers against the indicated RIs (all of which encode peptides presented by MHC class I). Results are presented as the RI inclusion/exclusion ratios plotted as Box and whiskers (i). An immunoblot is included to demonstrate input levels of E2F1, and SDMe is used as a control for T1-44 activity (ii); n=3 or 4 biologically independent experiments (each with 3 technical triplicates); statistics were performed by one-

way ANOVA and * marks adjusted P value <0.05, ** marks adjusted P value <0.01 adjusted P values.

E) (i) Polysome profiling assay from HCT116 cells, indicating total RNA quantity detected in each collected fraction; n=3; (ii) Polysome profiling assays for RIs are displayed. Data are presented as mean of the percentage of total RNA in each fraction n = 3; (iii) Polysome profiling assay for GAPDH coding transcript is displayed as a positive control. Data are presented as mean of the percentage of total RNA in each fraction n = 3; (iv) Graphical representation of total percentage in polysomal and non-polysomal fractions. Data are presented as mean ± SEM; n = 3.

F) Polysome profiling RNA-seq analysis of HCT116 cells following T1-44 treatment. (i) Number of RNA-seq reads mapping to annotated intronic regions in polysome-associated RNA from DMSO- and T1-44-treated HCT116 cells. Data represent biological replicates (n = 2); (ii) Heatmap representation of normalised intronic read counts across samples shown in (i). Colour scale indicates relative abundance over mean reads number (red - higher read density; blue - lower read density); Representative sashimi plots from two independent DMSO-treated biological replicates showing AGAP1 (iii) and MIB1 (iv) intronic loci.

G) dsRNA J2 RIP-seq analysis in A549 cells (GEO accession number GSE174374). (i) Heatmap and scatter plot (ii; J2 enrichment over input) representation of normalised intronic read counts (peptide coding RIs detected in the HCT116 immunopeptidomics) across input and J2-IP samples. (iii) pie chart represents the number of peptide coding RIs detected in dsRNA RIP-seq data. Colour scale indicates relative abundance over mean reads number (red - higher read density; blue - lower read density); n = 2; Representative sashimi plots from two independent input and J2 dsRNA RIP biological replicates showing NSD1 (iv) and SETD5 (iv) intronic loci.

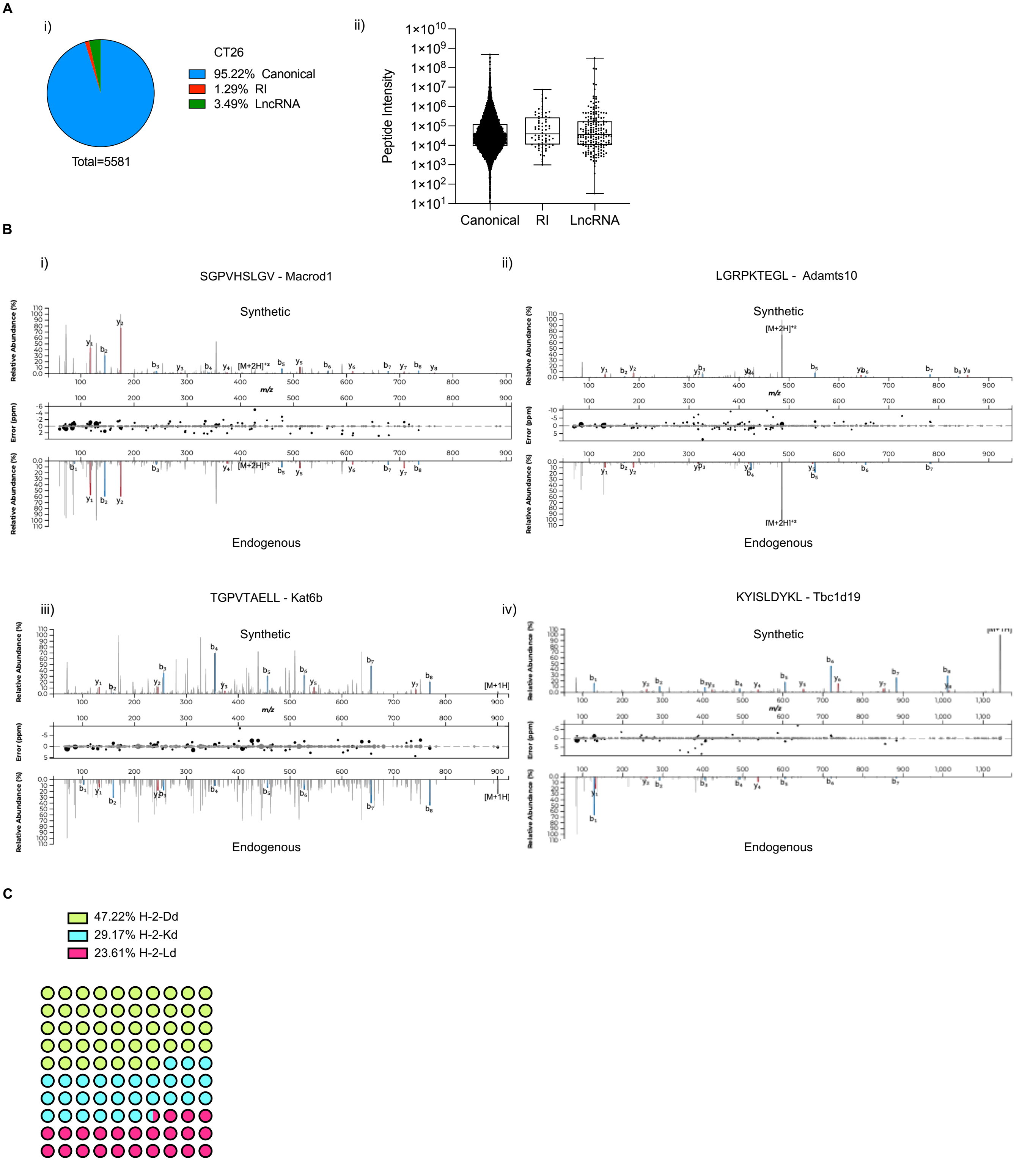
A
i)
CT26
95.22% Canonical
1.29% RI
3.49% LncRNA
Total=5581
ii)
Peptide Intensity
Canonical
RI
LncRNA
B
i)
SGPVHSLGV - Macrod1
ii)
LGRPKTEGL - Adamts10
Synthetic
Endogenous
Relative Abundance (%)
Error (ppm)
m/z
iii)
TGPVTAELL - Kat6b
iv)
KYISLDYKL - Tbc1d19
C
47.22% H-2-Dd
29.17% H-2-Kd
23.61% H-2-Ld

Appendix Figure S6. Immunopeptidomics of CT26 cells.

A) (i) Pie charts showing the distribution of identified peptides across different categories in CT26. Results presented as percentage of total unique peptides assigned to each category; (ii) Distribution of MS signal intensity for each peptide across different categories in CT26 cells. Data are derived from two independent biological replicates analysed by LC–MS/MS.

B) Synthetic peptides corresponding to MHC class I bound peptides identified from the CT26 immunopeptidomics analysis were synthesised and analysed by mass spectrometry to confirm the identifications made. Examples are shown of the peptides encoded by RIs, SGPVHSLGV (i), LGRPKTEGL (ii), TGPVTAELL (iii), and KYISLDYKL (iv). The bottom mass spectrometry profile represents the peptide identification from the endogenous CT26 experiment, whilst the upper spectrogram represents the ion profile from the synthetic peptide.

C) Predicted MHC allele frequency for identified RI-derived peptides is displayed as a percentage of total.

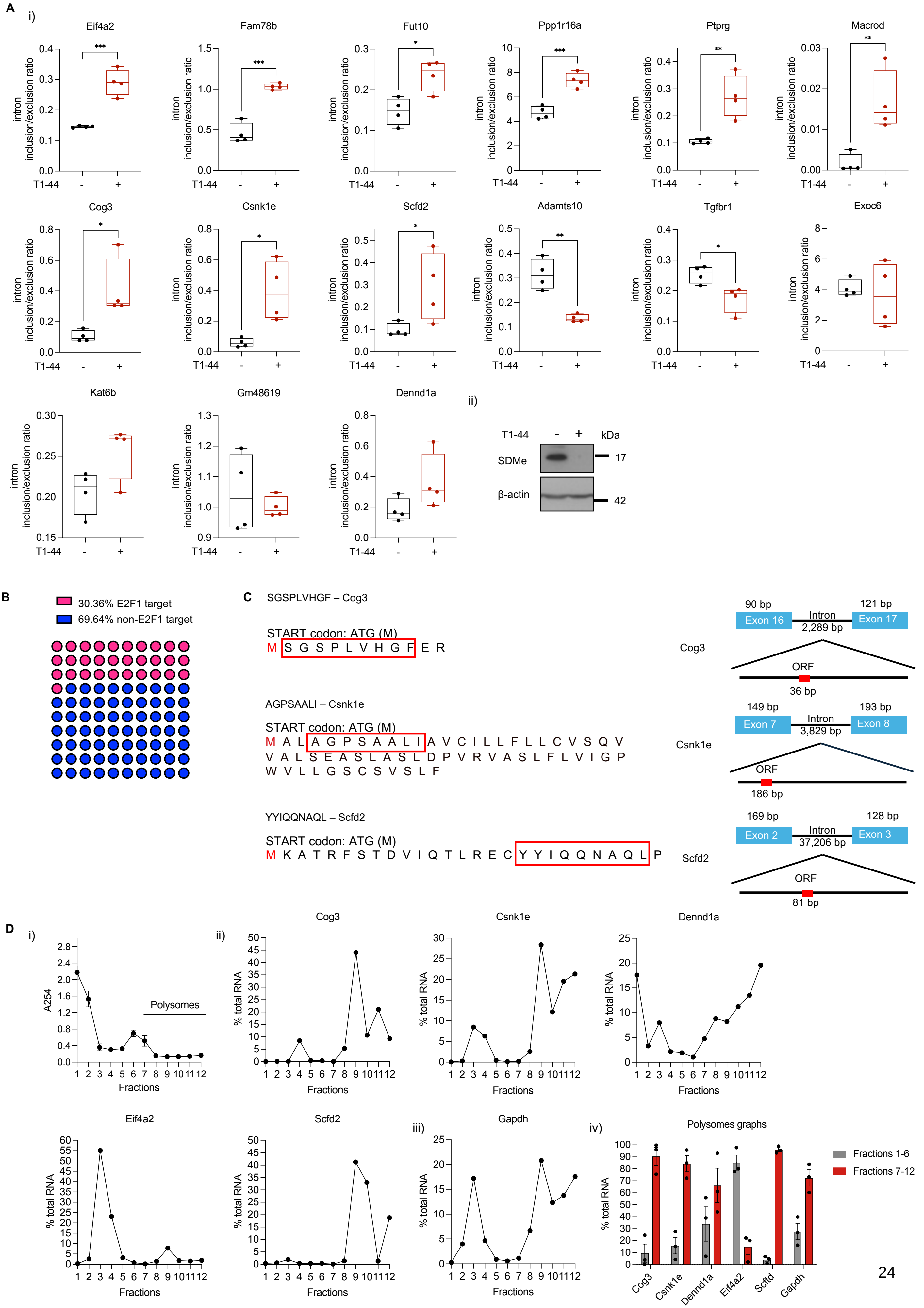
A
i)
Eif4a2
Fam78b
Fut10
Ppp1r16a
Ptprg
Macrod
Cog3
Csnk1e
Scfd2
Adamts10
Tgfbr1
Exoc6
Kat6b
Gm48619
Dennd1a
intron inclusion/exclusion ratio
T1-44
ii)
T1-44
kDa
SDMe
17
β-actin
42
B
30.36% E2F1 target
69.64% non-E2F1 target
C
SGSPLVHGF – Cog3
START codon: ATG (M)
M S G S P L V H G F E R
AGPSAALI – Csnk1e
START codon: ATG (M)
M A L A G P S A A L I A V C I L L F L L C V S Q V
V A L S E A S L A S L D P V R V A S L F L V I G P
W V L L G S C S V S L F
YYIQQNAQL – Scfd2
START codon: ATG (M)
M K A T R F S T D V I Q T L R E C Y Y I Q Q N A Q L P
Cog3
90 bp
Exon 16
Intron
2,289 bp
121 bp
Exon 17
ORF
36 bp
Csnk1e
149 bp
Exon 7
Intron
3,829 bp
193 bp
Exon 8
ORF
186 bp
Scfd2
169 bp
Exon 2
Intron
37,206 bp
128 bp
Exon 3
ORF
81 bp
D
i)
A254
Polysomes
Fractions
ii)
Cog3
Csnk1e
Dennd1a
Eif4a2
Scfd2
% total RNA
Fractions
iii)
Gapdh
iv)
Polysomes graphs
Fractions 1-6
Fractions 7-12
% total RNA
Cog3
Csnk1e
Dennd1a
Eif4a2
Scftd
Gapdh

Appendix Figure S7. Immunopeptidomics of CT26 cells

A) (i) CT26 cells were treated for 48 h with 1 µM T1-44 or DMSO as indicated. RNA was isolated and used in qRT-PCR analysis with primers against the indicated RIs (all of which encode peptides presented by MHC class I). Results are presented as inclusion/exclusion ratios. (ii) An immunoblot is included to demonstrate input levels of SDMe is used as a control for T1-44 activity; n=4 biologically independent experiments (each with 3 technical triplicates); Box and whiskers are defined as minimum, first quartile, median, third quartile, and maximum of data; statistics were performed by one-way ANOVA and * marks adjusted P value <0.05, ** marks adjusted P value <0.01 adjusted P values, *** marks adjusted P value <0.001.
B) The percentage of corresponding RI genes giving rise to MHC class I-bound peptides that score as potential direct E2F1 target genes. Analysis was performed on all 56 unique peptide-coding RI containing genes identified.
C) Examples of RI-derived open reading frames are displayed. The predicted ORF (left panel) giving rise to the identified MHC class I-bound peptide (boxed in red). Potential start residues are highlighted in red text. Graphical representation of predicted ORF position within the intron is also presented (right panel).
D) (i) Polysome profiling assay from CT26 cells, indicating total RNA quantity detected in each collected fraction; n=3; (ii) Polysome profiling assays for RIs are displayed. Data are presented as mean of the percentage of total RNA in each fraction n = 3; (iii) Polysome profiling assay for Gapdh coding transcript is displayed as a positive control. Data are presented as mean of the percentage of total RNA in each fraction n = 3; (iv) Graphical representation of total percentage in polysomal and non-polysomal fractions. Data are presented as mean ± SEM; n = 3.

A

Vaccine order	Pool	Sequence	Accession	Intron position	Gene name	Max fold change	Highest mean condition	Lowest mean condition	length	netMHCpan score	netMHC
1	A1	SPVLIHSFL	chr15:76671763-76690171_frame=2	Intron 1	Ppp1r16a	1.09	Treated	Control	9	0.0068	H-2-Ld
2	A2	SPGDLGLSL	chr19:37550566-37570382_frame=3	Intron 2	Exoc6	1.01	Control	Treated	9	0.0084	H-2-Ld
3	A3	TGPVTAELL	chr14:21517499-21609753_frame=1	Intron 2	Kat6b	1.21	Control	Treated	9	0.0084	H-2-Dd
4	A4	KYISLDYKL	chr5:53889408-53896990_frame=2	Intron 18	Tbc1d19	1.08	Treated	Control	9	0.0138	H-2-Kd
5	A5	SGPVHSLGV	chr19:7185606-7196413_frame=1	Intron 4	Macrod1	1.47	Treated	Control	9	0.0311	H-2-Dd
6	A6	KPVTRLNSL	chr14:11554333-11721114_frame=1	Intron 1	Ptprg	2.57	Treated	Control	9	0.1046	H-2-Ld
7	B1	SYLSSPWL	chr14:21517499-21609753_frame=1	Intron 2	Kat6b	1.03	Control	Treated	8	0.1108	H-2-Kd
8	B2	GPVGVGISL	chr8:31188407-31194953_frame=3	Intron 2	Fut10	1.79	Control	Treated	9	0.1635	H-2-Ld
9	B3	SDFPLYSSF	chr4:47383995-47393246_frame=2	Intron 2	Tgfbr1	1.33	Control	Treated	9	0.4954	H-2-Ld
10	B4	SFWANIHL	chr12:6699927-6795962_frame=1	Intron 1	RP23-478I20.1 (Gm48619)	1.71	Control	Treated	8	0.6554	H-2-Kd
11	B5	LGRPKTEGL	chr17:33549610-33550465_frame=3	Intron 21	Adamts10	1.81	Treated	Control	9	1.2252	H-2-Dd
12	B6	IYGGETIRGL	chr16:23110724-23111125_frame=2	Intron 7	Eif4a2	1.21	Control	Treated	10	1.6336	H-2-Kd
13	C1	ESSDLAPKF	chr1:167001829-167078536_frame=3	Intron 1	Fam78b	1.08	Control	Treated	9	1.7559	H-2-Dd
14	C2	KYIQSKTSI	chr5:36543298-36571295_frame=-3	Intron 2	Tbc1d14	1.80	Treated	Control	9	0.0009	H-2-Kd
15	C3	LGPPYSLNF	chr11:90548988-90571721_frame=-1	Intron 12	Stxbp4	1.35	Control	Treated	9	0.0054	H-2-Dd
16	C4	YYIQQNAQL	chr5:74482278-74519483_frame=-1	Intron 2	Scfd2	1.46	Control	Treated	9	0.0077	H-2-Kd
17	C5	YGSGRFFLL	chr10:91122044-91123557_frame=-2	Intron 2	Slc25a3	1.12	Treated	Control	9	0.0236	H-2-Dd
18	C6	AGPSAALI	chr15:79421011-79424839_frame=-1	Intron 7	Csnk1e	1.07	Control	Treated	8	0.0319	H-2-Dd
19	D1	SYLSYIMGL	chr18:38239190-38250177_frame=-3	Intron 1	1700086O06Rik	2.46	Control	Treated	9	0.0353	H-2-Kd
20	D2	SGEPRLIEL	chr1:105979625-105989018_frame=-1	Intron 2	Gm7160	1.24	Treated	Control	9	0.0874	H-2-Dd
21	D3	MGPHKIAKLL	chr5:74482278-74519483_frame=-3	Intron 2	Scfd2	4.39	Treated	Control	10	0.0926	H-2-Dd
22	D4	VPHVPSATGF	chr1:173927223-173935009_frame=-1	Intron 4	Ifi203	3.18	Control	Treated	10	0.0986	H-2-Ld
23	D5	AVPGKAFNL	chr14:101507691-101607962_frame=-1	Intron 1	Tbc1d4	1.40	Treated	Control	9	0.1003	H-2-Dd
24	D6	SFLLTKSML	chr19:8818685-8819020_frame=-1	Intron 1	Ttc9c	2.43	Treated	Control	9	0.1121	H-2-Kd
25	E1	LPSFSLRSL	chr4:148083994-148087916_frame=-1	Intron 1	Agtrap	1.01	Control	Treated	9	0.1175	H-2-Ld
26	E2	SGSPLVHGF	chr14:75719813-75722101_frame=-2	Intron 16	Cog3	1.27	Treated	Control	9	0.1293	H-2-Dd
27	E3	SPWRNSLL	chr2:37802274-37816915_frame=-1	Intron 20	Dennd1a	1.20	Treated	Control	8	0.1391	H-2-Ld
28	E4	LYRNSFVFL	chr16:31261308-31265304_frame=-3	Intron 2	Ppp1r2	1.15	Treated	Control	9	0.1743	H-2-Kd
29	E5	TYMLHVYL	chr2:128331050-128429093_frame=-3	Intron 1	Morrbid	1.16	Treated	Control	8	0.1997	H-2-Kd

B

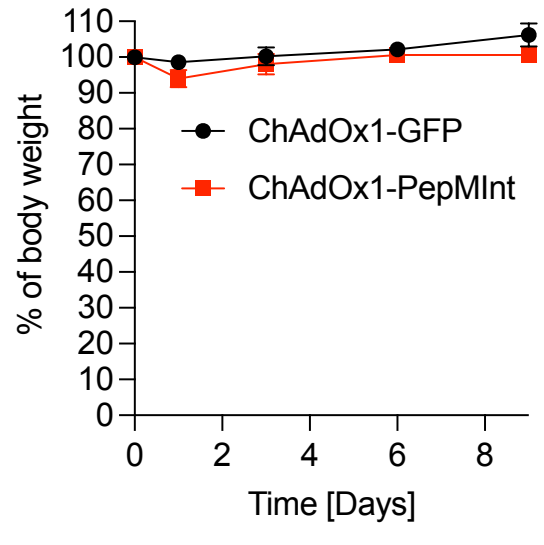

Appendix Figure S8. Immunogenicity of ChAdOx1-PepMInt vaccine.

A) Characterisation of the 29 selected peptides encoded by murine RIs identified in the immunopeptidomics experiment on CT26 cells treated with T1-44, with respect to DMSO control. These peptides were used to generate the poly-antigen cassette expressed from the ChAdOx1 viral vector. In order from the left, columns characterise: order in the vector; pool which peptide has been tested in; sequence of the peptide; accession – RI coordinates and frame; Intron position; RI gene name; Peptide abundance fold change; highest and lowest condition; peptide length; NetMHCpan 4.1 score and allele columns show the results from binding affinity prediction analysis.

B) Relative body weight of BALB/c mice presented as a mean value ± SEM, n=5 mice; please see Figure 4E-F.

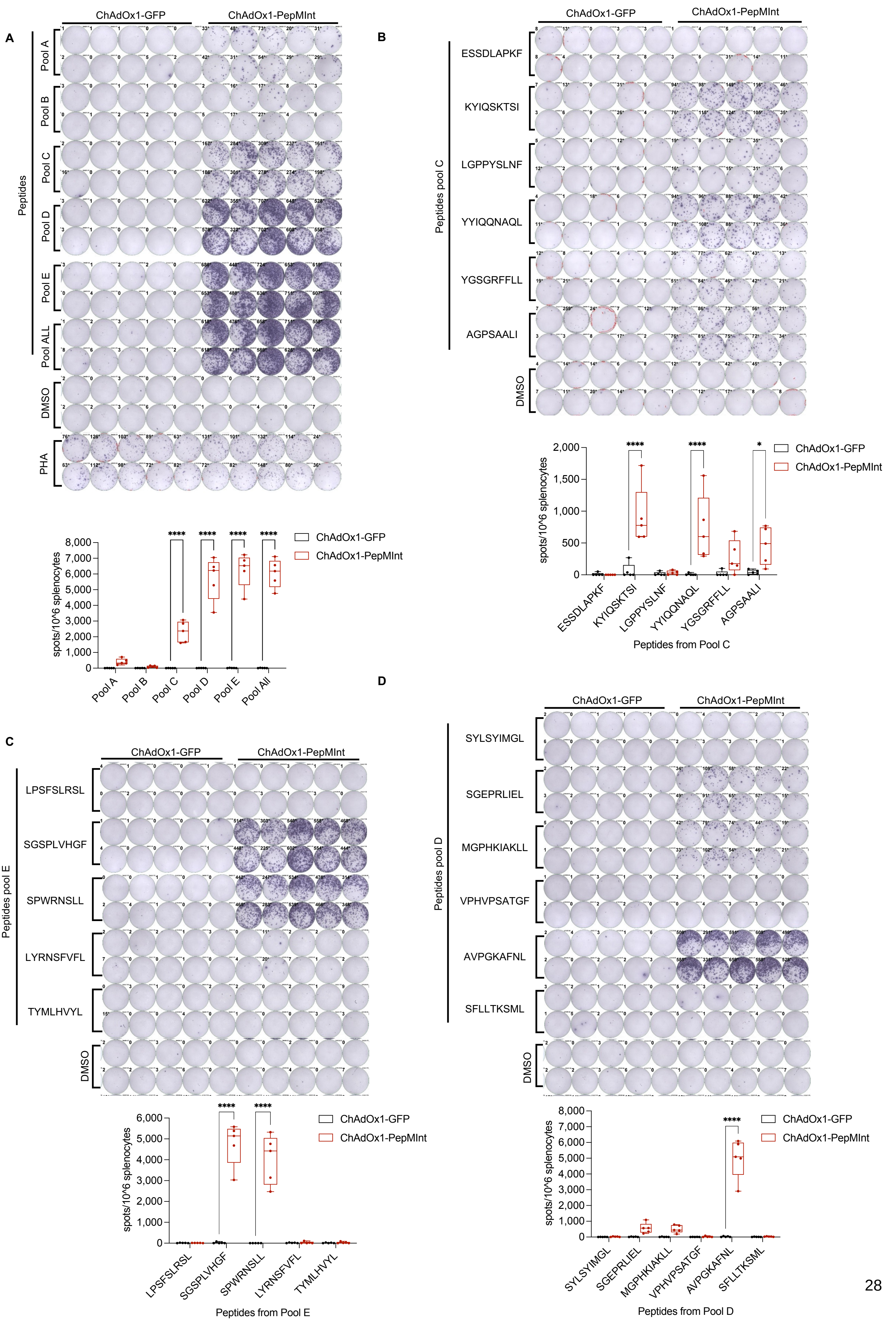
A
ChAdOx1-GFP
ChAdOx1-PepMInt
Peptides
Pool A
Pool B
Pool C
Pool D
Pool E
Pool ALL
DMSO
PHA
spots/10^6 splenocytes
8,000
7,000
6,000
5,000
4,000
3,000
2,000
1,000
0
****
Pool A
Pool B
Pool C
Pool D
Pool E
Pool All
B
Peptides pool C
ESSDLAPKF
KYIQSKTSI
LGPPYSLNF
YYIQQNAQL
YGSGRFFLL
AGPSAALI
2,000
1,500
1,000
500
*
Peptides from Pool C
C
Peptides pool E
LPSFSLRSL
SGSPLVHGF
SPWRNSLL
LYRNSFVFL
TYMLHVYL
Peptides from Pool E
D
Peptides pool D
SYLSYIMGL
SGEPRLIEL
MGPHKIAKLL
VPHVPSATGF
AVPGKAFNL
SFLLTKSML
Peptides from Pool D

Appendix Figure S9. Immunogenicity of ChAdOx1-PepMInt vaccine.

A) Splenocytes were stimulated with pools of peptides contained within the poly-antigen cassette of the ChAdOx1-PepMInt vaccine, and activity was measured in interferon-γ-based ELISpot. DMSO and PHA were used as negative and positive controls respectively; the data were plotted as Box and whiskers; Student's t test; **** marks adjusted P value <0.0001. The PHA, DMSO and Pool All ELISpot data are also presented in Figure 4F as part of the same experiment.

B-D) Splenocytes were stimulated with single peptides (from pools C-E) contained within the poly-antigen cassette, and activity was measured by interferon-γ-based ELISpot. DMSO and PHA were used as negative and positive controls respectively and the data plotted as Box and whiskers; Student's t test; * adjusted p-value <0.05, **** marks adjusted p-value <0.0001.

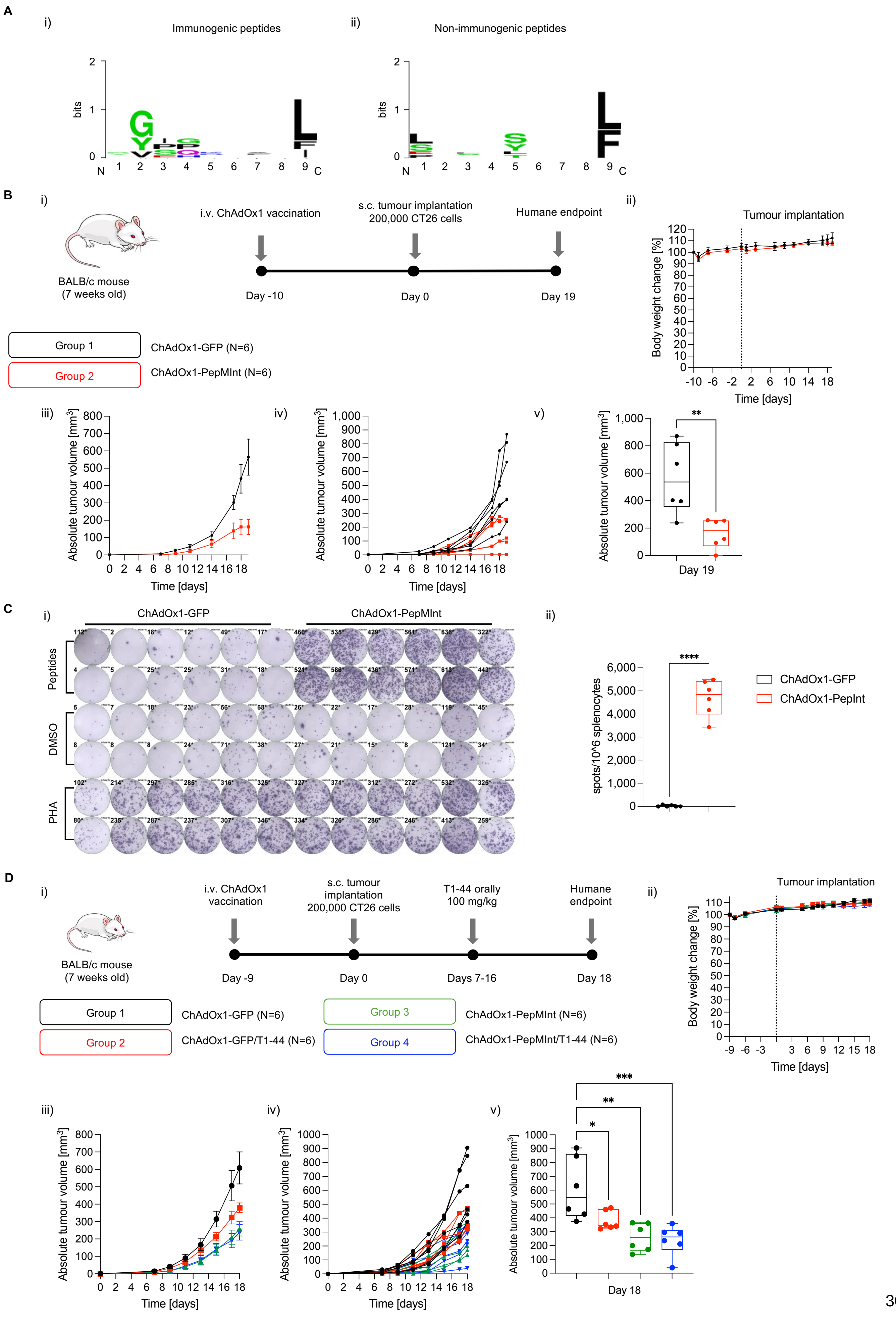
A
i)
Immunogenic peptides
ii)
Non-immunogenic peptides
bits
N
C
B
i)
BALB/c mouse
(7 weeks old)
i.v. ChAdOx1 vaccination
s.c. tumour implantation
200,000 CT26 cells
Humane endpoint
Day -10
Day 0
Day 19
ii)
Tumour implantation
Body weight change [%]
Time [days]
Group 1
ChAdOx1-GFP (N=6)
Group 2
ChAdOx1-PepMInt (N=6)
iii)
iv)
v)
Absolute tumour volume [mm³]
Time [days]
**
Day 19
C
i)
ChAdOx1-GFP
ChAdOx1-PepMInt
Peptides
DMSO
PHA
ii)
****
spots/10^6 splenocytes
ChAdOx1-GFP
ChAdOx1-PepInt
D
i)
BALB/c mouse
(7 weeks old)
i.v. ChAdOx1
vaccination
s.c. tumour
implantation
200,000 CT26 cells
T1-44 orally
100 mg/kg
Humane
endpoint
Day -9
Day 0
Days 7-16
Day 18
Group 1
ChAdOx1-GFP (N=6)
Group 2
ChAdOx1-GFP/T1-44 (N=6)
Group 3
ChAdOx1-PepMInt (N=6)
Group 4
ChAdOx1-PepMInt/T1-44 (N=6)
ii)
Tumour implantation
Body weight change [%]
Time [days]
iii)
iv)
v)
Absolute tumour volume [mm³]
Time [days]
***
**
*
Day 18

E

i)

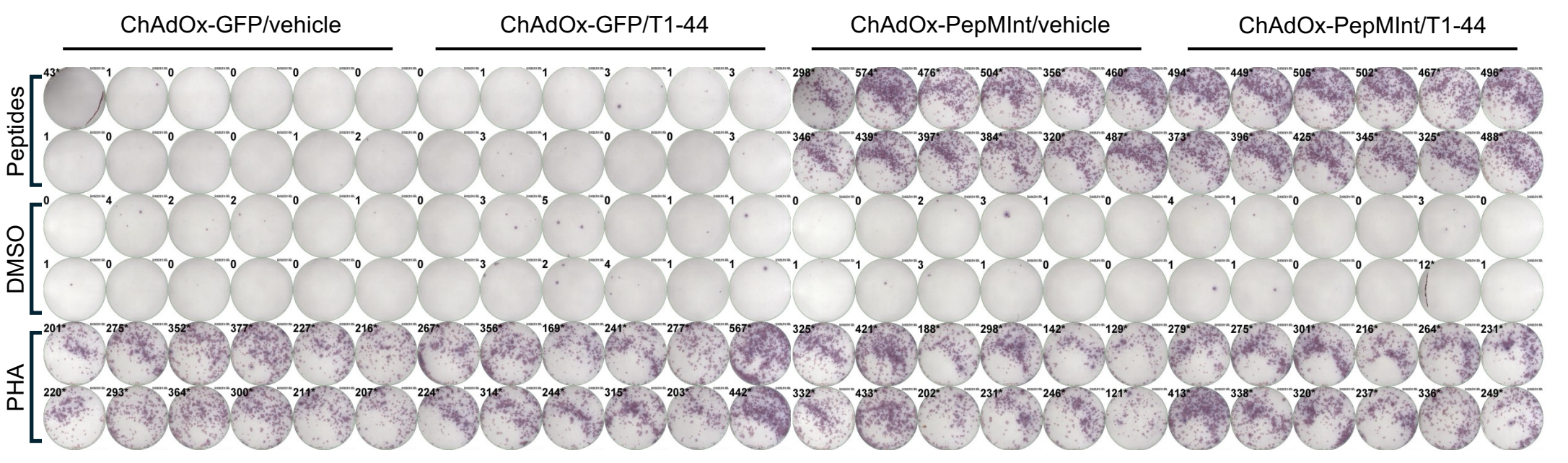
ChAdOx-GFP/vehicle
ChAdOx-GFP/T1-44
ChAdOx-PepMInt/vehicle
ChAdOx-PepMInt/T1-44
Peptides
DMSO
PHA

ii)

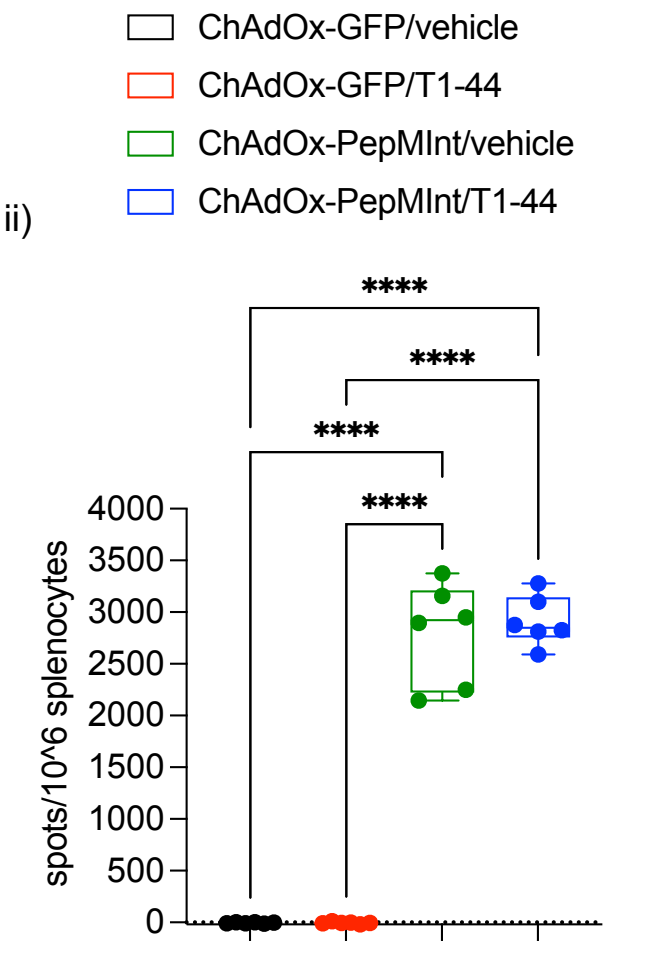
ChAdOx-GFP/vehicle
ChAdOx-GFP/T1-44
ChAdOx-PepMInt/vehicle
ChAdOx-PepMInt/T1-44
****
****
****
****
spots/10^6 splenocytes
4000
3500
3000
2500
2000
1500
1000
500
0

F

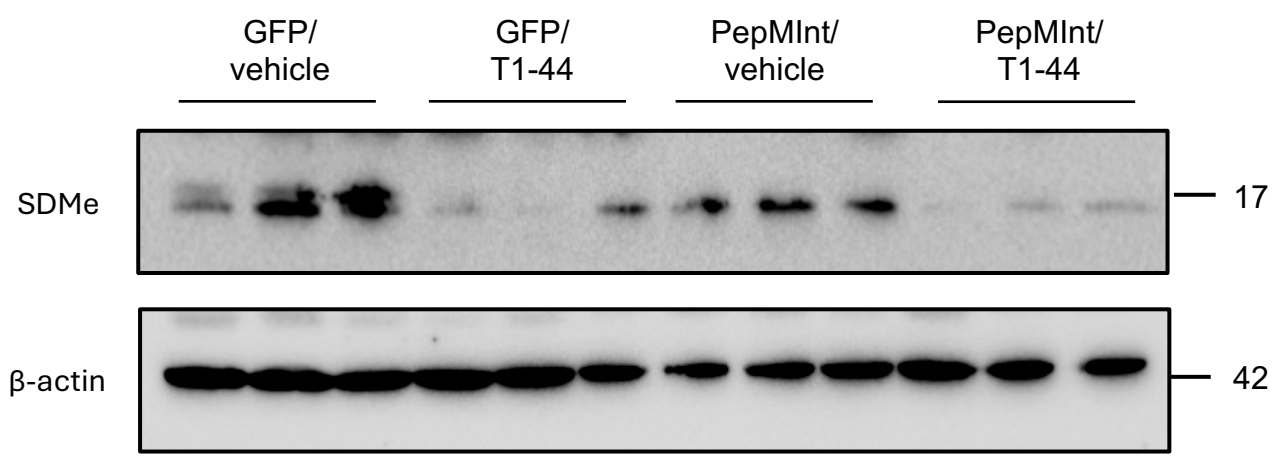
GFP/
vehicle
GFP/
T1-44
PepMInt/
vehicle
PepMInt/
T1-44
SDMe
β-actin
17
42

Appendix Figure S10. Immunogenicity of ChAdOx1-PepMInt vaccine.

A) Sequence logos of amino acid conservation in immunogenic (i) and non-immunogenic (ii) RI-derived peptides.

B) Groups of 6 BALB/c mice were vaccinated with ChAdOx1-PepMInt or ChAdOx1-GFP. At 10 days post-vaccination mice were subcutaneously injected with the 200,000 CT26 cells (i); (ii) Relative body weight of BALB/c mice presented as a mean value ± SEM; (iii) Absolute tumour volume is presented as mean ± SEM; (iv) Absolute tumour volumes are presented in the individual mice; (v) Absolute tumour volume of individual mice at day 19 (Student's t test; ** p-value), plotted as Box and whiskers. The experiment was performed twice with successful replication.

C) Splenocytes collected in the tumour challenge experiment (see Figure S10B) were stimulated with pool of peptides contained within the poly-antigen cassette, and activity was measured in interferon-γ-based ELISpot (i). DMSO and PHA were used as negative and positive controls respectively; plotted as Box and whiskers (ii); Student's t test; **** marks adjusted P value <0.0001.

D) Groups of 12 BALB/c mice were vaccinated with ChAdOx1-PepMInt or ChAdOx1-GFP. At 9 days post-vaccination mice were subcutaneously injected with the 200,000 CT26 cells. From day 7, half of the mice from each group were daily treated (for 10 days) by oral administration (gavage) with 100 mg/kg (dosing volume 10 ml/kg) of T1-44 (i); (ii) Relative body weight of BALB/c mice presented as a mean value ± SEM; (iii) Absolute tumour volume is presented as mean ± SEM; (iv) Absolute tumour volumes are presented in the individual mice; (v) Absolute tumour volume of individual mice at day 18 (one-way ANOVA and * marks adjusted P value <0.05, ** marks adjusted P value <0.01 adjusted P values, *** marks adjusted P value <0.001 adjusted P values), plotted as Box and whiskers. The experiment was performed once.

E) Splenocytes collected in the tumour challenge experiment (see Figure S10D) were stimulated with pool of peptides contained within the poly-antigen cassette, and activity was measured in interferon-γ-based ELISpot (i). DMSO and PHA were used as negative and positive controls respectively; plotted as Box and whiskers (ii); Student's t test; **** marks adjusted P value <0.0001.

F) An immunoblot is included to demonstrate input levels of SDMe used as a control for T1-44 activity in the experiment presented in the Figure S10D.

A

	Sequence	Gene Name	NetMHCpan prediction	NetMHCpan score
Peptide 1	ALAKELDSV	NSD1	HLA-A02:01	0.0474
Peptide 2	ELIEKLVEI	SETD5	HLA-A02:01	0.0563
Peptide 3	RILTPIVSL	FAM188B	HLA-A02:01	0.1985
Peptide 4	GLDLKLVMI	PDGFRL	HLA-A02:01	0.3948
Peptide 5	ILTALKIYI	MAPK6	HLA-A02:01	0.4419
Peptide 6	SLSFHVGTV	BCAS3	HLA-A02:01	0.6773
Peptide 7	LLNGVVSV	RSPRY1	HLA-A02:01	0.9311

B

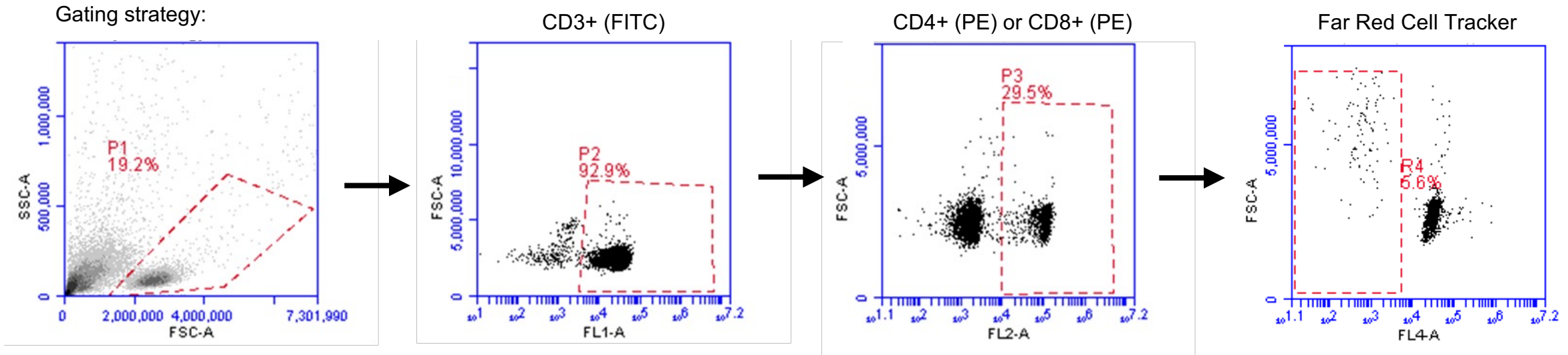

C

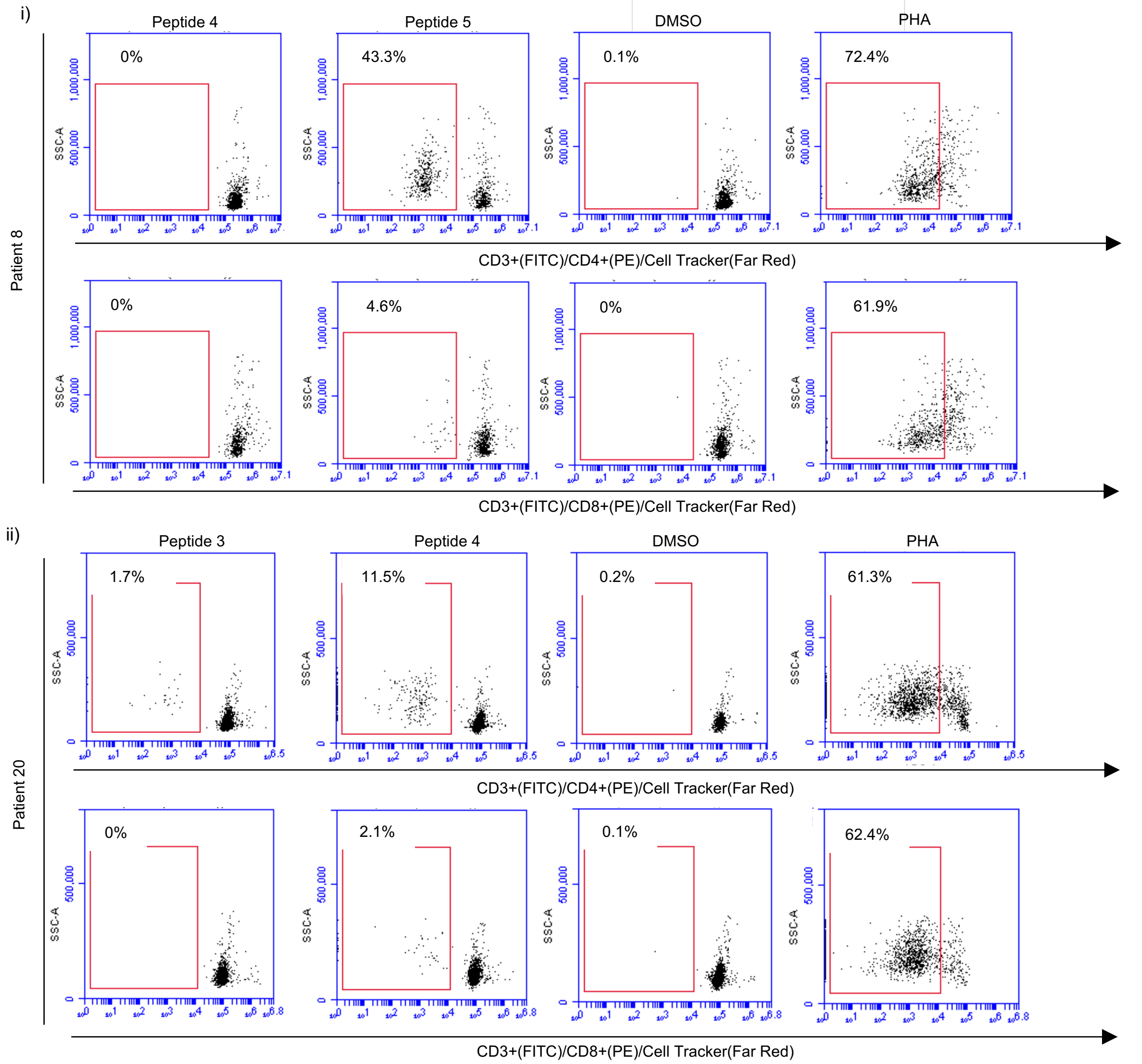

D

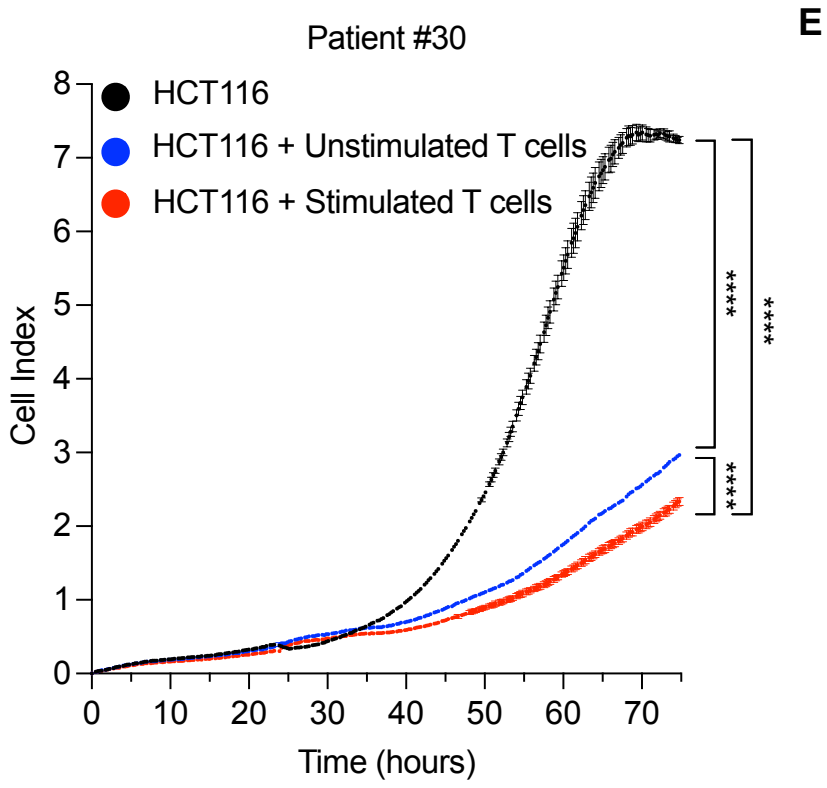

E

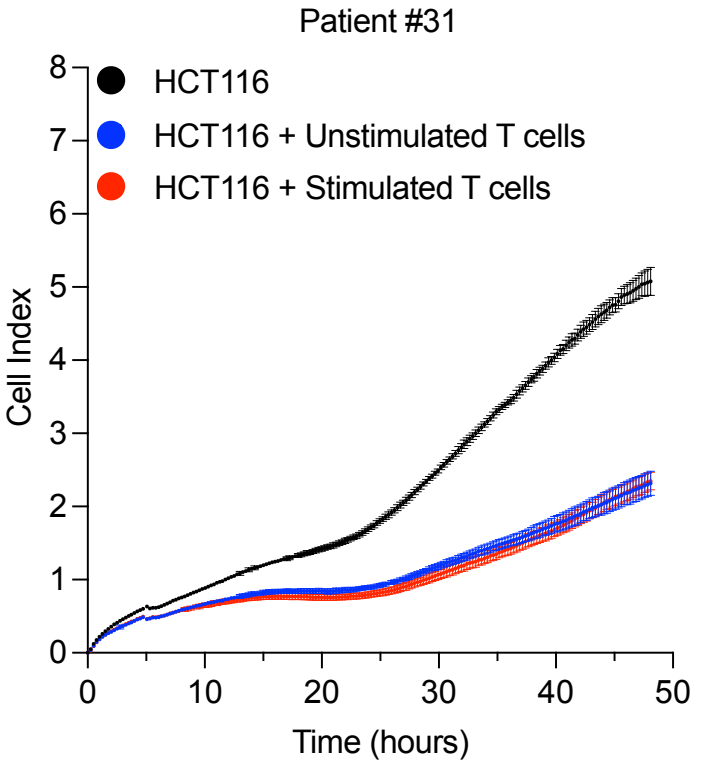

Appendix Figure S11. Immunogenicity of the human RI-derived peptides.

A) Characteristic of the seven HLA-A02 selective RI-derived peptides identified in the human HCT116 immunopeptidomics study.
B) Gating strategy of Flow Cytometry-based Cell Tracker Proliferation Assay.
C) Example of the results of T cell proliferation assay conducted on PBMCs from human donors 8 (i) and 20 (ii). PBMCs were isolated from CRC patients and stained with Cell tracker and incubated for 9-12 days with individual peptides; DMSO and PHA-L treatments were used as negative and positive controls, respectively. The cells were counterstained with $CD3^{+}$, $CD4^{+}$, and $CD8^{+}$ antibodies and populations of the cells were measured by flow cytometry.
D) Killing assay performed with HCT116 cells treated with T cells enriched from PBMCs (Patient #30) that had been stimulated (red line) or unstimulated (blue line) with pool of seven RI-derived peptides, compared to untreated control (black line). Results presented as a mean ± SEM calculated from the data of three sets of T cells isolated and expanded from one Patient. One-way ANOVA, **** marks adjusted p-value <0.0001.
E) Killing assay performed with HCT116 cells treated with T cells enriched from PBMCs (Patient #31) that had been stimulated (red line) or unstimulated (blue line) with pool of seven RI-derived peptides, compared to untreated control (black line). Results presented as a mean ± SEM calculated from the data of three sets of T cells isolated and expanded from one Patient.

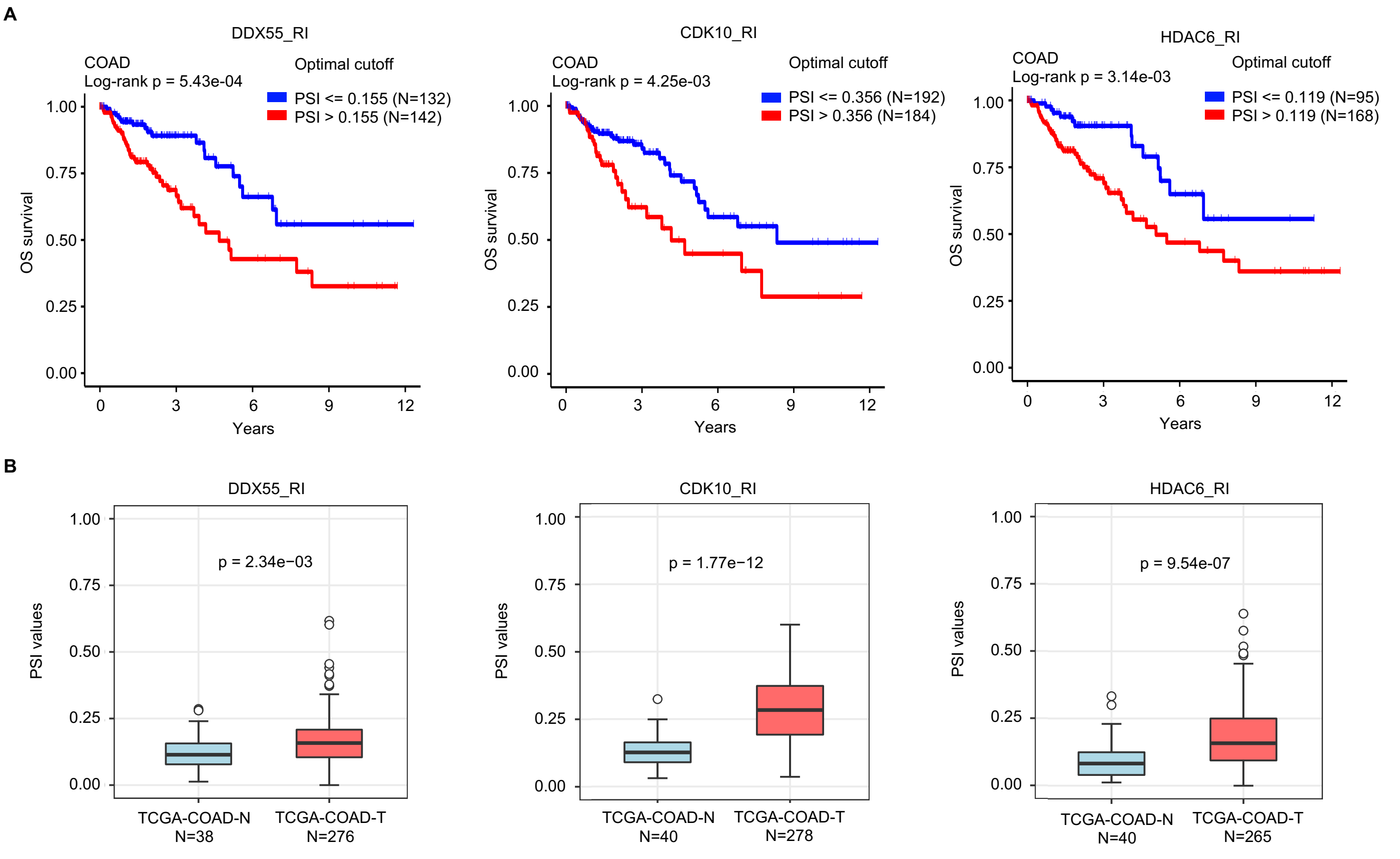
A
DDX55_RI
COAD
Log-rank p = 5.43e-04
Optimal cutoff
PSI <= 0.155 (N=132)
PSI > 0.155 (N=142)
OS survival
Years
CDK10_RI
COAD
Log-rank p = 4.25e-03
Optimal cutoff
PSI <= 0.356 (N=192)
PSI > 0.356 (N=184)
HDAC6_RI
COAD
Log-rank p = 3.14e-03
Optimal cutoff
PSI <= 0.119 (N=95)
PSI > 0.119 (N=168)
B
DDX55_RI
p = 2.34e−03
PSI values
TCGA-COAD-N
N=38
TCGA-COAD-T
N=276
CDK10_RI
p = 1.77e−12
TCGA-COAD-N
N=40
TCGA-COAD-T
N=278
HDAC6_RI
p = 9.54e-07
TCGA-COAD-N
N=40
TCGA-COAD-T
N=265

Appendix Figure S12. Level of RIs in human cancer.

A) Overall Survival of Colon Cancer Patients dependent on expression of selected RIs. KM plots were produced for the particular RI event based on the optimal PSI cut-off.
B) Expression of selected RI transcript in TCGA tumour samples compared with normal tissue; PSI values are presented as a Boxplot; The Wilcoxon rank-sum test was used to evaluate the significance of differences; TCGA-COAD-N – normal tissue was collected from Colorectal Adenocarcinoma patients; TCGA-COAD-T – tumour tissue collected from Colorectal Adenocarcinoma patients.
