# Supplementary material for: PRMT5-mediated intron retention triggers innate and adaptive immunity against cancer
Source: EMBO Mol Med. 2026 Aug 17;18(9):3677–703. doi: 10.1038/s44321-026-00499-1 (PMC13562731; doi:10.1038/s44321-026-00499-1)
Supplement: Supplementary file 2 [file 44321_2026_499_MOESM3_ESM.pdf]

EMBO Molecular Medicine

PRMT5-mediated intron retention triggers innate and adaptive immunity against cancer

Wiktoria Blaszczak, Wojciech Barczak, Chuyue Zhang, Garret Rochford, Annalisa Nicastri, Claire Hutchings, Anastasia Samsonova, Alexander Kanapin, Nicola Ternette, Paul Klenerman, and Nicholas La Thangue

Corresponding authors: Nicholas La Thangue (Nick.LaThangue@oncology.ox.ac.uk)

Review Timeline:

Submission Date:	6th Jan 26
Editorial Decision:	2nd Feb 26
Revision Received:	22nd May 26
Editorial Decision:	10th Jul 26
Revision Received:	16th Jul 26
Accepted:	24th Jul 26

Editor: Lise Roth

Transaction Report:

(Note: With the exception of the correction of typographical or spelling errors that could be a source of ambiguity, letters and reports are not edited. Depending on transfer agreements, referee reports obtained elsewhere may or may not be included in this compilation. Referee reports are anonymous unless the Referee chooses to sign their reports.)

2nd Feb 2026

Dear Prof. La Thangue,

Thank you for the submission of your manuscript to our editorial offices. We have now received feedback from the three reviewers who agreed to evaluate your study. As you will see from the reports below, the reviewers acknowledge the novelty, quality and translational potential of the findings, but nevertheless raise a few major concerns. If you feel you can address the reviewers' comments satisfactorily, you may wish to submit a revised version of your manuscript.

Addressing the reviewers' concerns in full will be necessary for further considering the manuscript in our journal, and acceptance of the manuscript will entail a second round of review. EMBO Molecular Medicine encourages a single round of revision only and therefore, acceptance or rejection of the manuscript will depend on the completeness of your responses included in the next, final version of the manuscript. For this reason, and to save you from any frustrations in the end, I would strongly advise against returning an incomplete revision.

If you would like to discuss further the points raised by the referees, I am available to do so via email or video. Let me know if you are interested in this option.

We are expecting your revised manuscript within three to four months, if you anticipate any delay, please contact us.

When preparing your revised manuscript, please refer to our guidelines: https://link.springer.com/journal/44321/submission-guidelines#cms-Revised-submissions. We perform an initial quality control of all revised manuscripts before re-review; failure to include requested items will delay the evaluation of your revision.

We require:

1) A .docx formatted version of the manuscript text (including legends for main figures, EV figures and tables). Please make sure that the changes are highlighted to be clearly visible.

2) Individual production quality figure files as .eps, .tif, .jpg (one file per figure). For guidance, download the 'Figure Guide PDF': https://media.springernature.com/original/springer-cms/rest/v1/content/27825798/data/v1.

3) A .docx formatted letter INCLUDING the reviewers' reports and your detailed point-by-point responses to their comments. As part of the EMBO Press transparent editorial process, the point-by-point response is part of the Review Process File (RPF), which will be published alongside your paper.

4) A complete author checklist, which you can download from our author guidelines. Please insert information in the checklist that is also reflected in the manuscript. The completed author checklist will also be part of the RPF.

5) Please note that all corresponding authors are required to supply an ORCID ID for their name upon submission of a revised manuscript.

6) It is mandatory to include a 'Data Availability' section after the Materials and Methods. Before submitting your revision, primary datasets produced in this study need to be deposited in an appropriate public database, and the accession numbers and database listed under 'Data Availability'. Please remember to provide a reviewer password if the datasets are not yet public.

In case you have no data that requires deposition in a public database, please state so in this section. Note that the Data Availability Section is restricted to new primary data that are part of this study.

7) For data quantification: please specify the name of the statistical test used to generate error bars and P values, the number (n) of independent experiments (specify technical or biological replicates) underlying each data point and the test used to calculate p-values in each figure legend. The figure legends should contain a basic description of n, P and the test applied. Graphs must include a description of the bars and the error bars (s.d., s.e.m.).

8) At EMBO Press we ask authors to provide source data for the main manuscript figures. You will receive a separate email with instructions for providing source data with your revised manuscript, including how to upload and organize the files.

9) Our journal encourages inclusion of *data citations in the reference list* to directly cite datasets that were re-used and obtained from public databases. Data citations in the article text are distinct from normal bibliographical citations and should directly link to the database records from which the data can be accessed. In the main text, data citations are formatted as follows: "Data ref: Smith et al, 2001" or "Data ref: NCBI Sequence Read Archive PRJNA342805, 2017". In the Reference list, data citations must be labeled with "[DATASET]". A data reference must provide the database name, accession

number/identifiers and a resolvable link to the landing page from which the data can be accessed at the end of the reference.

10) We replaced Supplementary Information with Expanded View (EV) Figures and Tables that are collapsible/expandable online. A maximum of 5 EV Figures can be typeset. EV Figures should be cited as 'Figure EV1, Figure EV2" etc... in the text and their respective legends should be included in the main text after the legends of regular figures.

- For the figures that you do NOT wish to display as Expanded View figures, they should be bundled together with their legends in a single PDF file called *Appendix*, which should start with a short Table of Content. Appendix figures should be referred to in the main text as: "Appendix Figure S1, Appendix Figure S2" etc.

- Additional Tables/Datasets should be labeled and referred to as Table EV1, Dataset EV1, etc. Legends have to be provided in a separate tab in case of .xls files. Alternatively, the legend can be supplied as a separate text file (README) and zipped together with the Table/Dataset file.

11) The paper explained: EMBO Molecular Medicine articles are accompanied by a summary of the articles to emphasize the major findings in the paper and their medical implications for the non-specialist reader. Please provide a draft summary of your article highlighting
- the medical issue you are addressing,
- the results obtained and
- their clinical impact.

This may be edited to ensure that readers understand the significance and context of the research. Please refer to any of our published articles for an example.

12) Author contributions: You will be asked to provide CRediT (Contributor Role Taxonomy) terms in the submission system. These replace a narrative author contribution section in the manuscript.

13) A "Disclosure and Competing Interests Statement" should be provided in the main text.

14) Every published paper includes a 'Synopsis' to further enhance discoverability. Synopses are displayed on the journal webpage and are freely accessible to all readers. They include a short stand first (maximum of 300 characters, including space) as well as 2-5 one-sentences bullet points that summarizes the paper. Please write the bullet points to summarize the key NEW findings. They should be designed to be complementary to the abstract - i.e. not repeat the same text. We encourage inclusion of key acronyms and quantitative information (maximum of 30 words / bullet point). Please use the passive voice. Please attach these in a separate file or send them by email, we will incorporate them accordingly.

Please also suggest a striking image or visual abstract to illustrate your article as a PNG file 550 px wide x 300-600 px high.

15) Include a Reagents and Tools Table as part of the Methods section, which can be downloaded from our author guidelines.

16) As part of the EMBO Publications transparent editorial process initiative (see our Editorial at http://embomolmed.embopress.org/content/2/9/329), EMBO Molecular Medicine will publish online a Review Process File (RPF) to accompany accepted manuscripts.
In the event of acceptance, this file will be published in conjunction with your paper and will include the anonymous referee reports, your point-by-point response and all pertinent correspondence relating to the manuscript. Let us know whether you agree with the publication of the RPF and as here, if you want to remove or not any figures from it prior to publication.
Please note that the Authors checklist will be published at the end of the RPF.

EMBO Molecular Medicine has a "scooping protection" policy, whereby similar findings that are published by others during review or revision are not a criterion for rejection. Should you decide to submit a revised version, I do ask that you get in touch after three months if you have not completed it, to update us on the status.

I look forward to receiving your revised manuscript.

Yours sincerely,

Lise Roth

Lise Roth, PhD
Senior Editor
EMBO Molecular Medicine

***** Reviewer's comments *****

Referee #1 (Comments on Novelty/Model System for Author):

The work is technically strong and novel, combining splicing analysis, immunopeptidomics, vaccination, and human T-cell assays to reveal a new PRMT5-intron retention-immune axis. Medical impact is currently moderate because translation to patients is limited by tumour heterogeneity, HLA restriction, and lack of in vivo human data, although the model systems used are appropriate.

Referee #1 (Remarks for Author):

The manuscript by Blaszczak and colleagues describes a conceptually novel mechanism by which PRMT5 regulates tumour immunogenicity through control of RIs. The authors show that PRMT5 inhibition leads to accumulation of retained introns that form dsRNA structures activating innate interferon responses and encode cryptic ORFs that generate immunogenic peptides presented by MHC I. Using a combination of RNAseq, immunopeptidomics, murine vaccination models, and ex vivo human Tcell assays, the study links aberrant splicing to both innate and adaptive immunity. Overall, the work is strong, and of high potential impact, with clear relevance to cancer immunotherapy and PRMT5-targeted strategies, however some mechanistic aspects, interpretations, and translational claims would benefit from further strengthening. Below are my main concerns,

Major comments

1. While the correlation between PRMT5 inhibition, RI accumulation, dsRNA formation, and IFN-β induction is clear, the manuscript would benefit from stronger causal evidence that RIs are the dominant drivers of the interferon response. For example, selective depletion of specific RI containing transcripts or rescue experiments restoring splicing of key introns could help distinguish RI driven effects from broader splicing disruption caused by PRMT5 inhibition

2. J2 staining is widely used but can detect multiple dsRNA species. It remains unclear to what extent dsRNA signals originate specifically from RIs versus other RNA species. The authors should discuss this limitation more explicitly and if possible provide additional evidence (RNA-FISH for specific RIs) to support RI specificity

3. The mechanism by which the cryptic ORFs are translated remains underexplored. Given the weak Kozak sequences and non-canonical start codons, the authors should discuss alternative initiation mechanisms (leaky scanning, non AUG initiation, ribosome reinitiation) and clarify whether ribosome profiling data or additional evidence could support active translation initiation within introns

4. Although RI derived peptides are clearly detectable and immunogenic, their relative abundance compared with canonical tumour antigens is not fully contextualised. Providing estimates of their contribution to the total MHC class I peptidome (percentage of total intensity or peptide counts) would help readers assess their biological and therapeutic relevance

5. The detection of RI reactive T cells in CRC patients is an important finding, but patient numbers are modest and responses heterogeneous. The authors should temper claims regarding clinical applicability and more clearly discuss inter-patient variability, HLA restriction, and how RI-based vaccines might be personalized or stratified in practice

6. Some phenotypes observed upon PRMT5 inhibition (interferon activation, increased MHC class I expression) may also arise from general splicing perturbation. A more explicit comparison between PRMT5 inhibition and other splicing perturbations (beyond pladienolide as a control) would help clarify whether PRMT5 has a unique role or primarily acts as a gateway to splicing stress induced immunogenicity

Minor comments
1. The manuscript alternates between "RI", "RIs", and "retained introns" frequently. A brief definitional reminder early in the Results and consistent usage thereafter would improve readability, especially for non-splicing specialists.

Referee #2 (Remarks for Author):

In this paper Blaszczak and colleagues identify a role for PRMT5 mediated intron retention in innate and adaptive immune responses in colorectal cancer. They find that PRMT5 inhibition leads to extensive intron retention and these introns are the source of IFN activating dsRNAs and neo-antigens. They further map these neo-antigens and generate a vaccine based on these peptides which has some efficacy in a mouse cancer model. They also find evidence of immune reactivity towards these neo-antigens in T cells from CRC patients. Together, this is an interesting study and with growing interest in activating immune responses in CRC, it is timely with potential implications for anti-cancer therapy. However, there are a number of issues that need to be addressed prior to publication.
Main points:
1) The IHC in Figure 2A is unconvincing. The authors need to control for background staining with RNAse treatment. Also, what is the model that is used and the treatment regime, I couldn't find the details of this in the paper? Further, what are the phenotypic effects of this treatment and proposed dsRNA accumulation on tumour growth, apoptosis, proliferation etc?
2) In Figure 2B how plausible is it that a single RI is detectable by the J2 dsRNA antibody and can lead to IFN activation. Is this a generic activation with any RI or specific to the ones induced by PRMT5 inhibition? Can the authors investigate the effects of other RNA sequences, including those predicted to form dsRNA by the ViennaRNA algorithm and those not.
3) To enhance the findings in Figure 2, can the authors pulldown dsRNA using J2 in their PRMT5 inhibitor treated cells to determine whether the DDX55 and CDK10 RIs can be detected using this method in cells without ectopic overexpression?
4) In Figure 3B there are almost as many downregulated peptides identified as upregulated. What do the authors propose is the reason for this?
5) In Figure 4E-F, what are the sequences that elicit the strongest immune responses in the mouse ELISAspots?
6) Tumour vaccine data is interesting but the level of tumour control is modest. This could be because the neo-antigens used for this vaccine were predominantly identified PRMT5 inhibition and therefore basal expression of them is low. Could the authors combine the vaccination with PRMT5 inhibitor treatment to determine if this increases efficacy?
7) In Figure 5B are the effects peptide specific or simply due to T cell activation? For example, what is the effect of co-culturing PHA stimulated T cells on HCT116 growth? Also, does PRMT5 inhibitor treatment enhance the effect of T cell killing?
8) The human RI data is confusing and counterintuitive. If the RI is leading to neo-antigen production that is recognised by T cells it would be expected to correlate with better survival due to increased immune control. Can the authors expand on this in the discussion?

Referee #3 (Remarks for Author):

In this study, the authors suggest that the arginine-methyltransferase PRMT5 is involved in coordinating innate and adaptive immune responses. The authors applied alternative splicing analyses to RNA-seq data and found that both PRMT5 and E2F1 regulate RNA splicing. Earlier research has shown that E2F1 is methylated by PRMT5, which enables its involvement in alternative splicing. If absent, intron retention is increased, and these regions have been shown to have a high propensity to form immunogenic dsRNA, activating innate immune signaling. Furthermore, they identified small peptides of RIs on the MHC class I complex, indicating the involvement of both innate and adaptive immunity. Following these findings, an RI-derived transgene was tested in animal experiments. In addition, T lymphocytes from human cancer patients demonstrated antigen specificity for RI-derived peptides, resulting in the killing of human tumor cells in vitro.
While the work is conceptually interesting, the mechanisms underlying the proposed model lack in-depth evaluation. Furthermore, while the effects of PRMT5 are comprehensively documented, the link to E2F1 is not as well described and remains rather elusive. Furthermore, the in vivo data require clarification regarding their design, and additional experiments are needed to evaluate the role of the immune system.
Major Concerns:
1) Figure 1A shows a heavily derivatized plot of RI data "normalized PSI values" without clear indication how data were normalized or what the cut-off is. Why do data need to be normalized? PSI is a percentage value, so absolute values can be used for the analysis. Also, the authors should show the browser pictures for 2-3 of the strongest effects.
2) As illustrated in Figure 1A (ii), the overlap of the Venn diagram is approximately 9% between the E2F1Cr and E2F1Cr/T1-44 condition. Could the authors please comment on these figures? If these RIs occur due to an interplay between E2F and PRMT5, should the overlap not be higher? What is the mechanistic explanation for that?
3) The authors claim in line 129 that RI events depend on E2F1 (and PRMT5). However, using the parameters in Figure S1B(iii) (10% PSI cutoff), the total number of RI events does not increase with E2F1 depletion. What would the bar chart look like with the parameters used in Figure 1? I acknowledge that Figure S1A displays a similar plot, but it would be clearer if the control was placed next to the other conditions instead of using a p-value as the cutoff.
4) In Figure 1B, the authors suggest that these RI events primarily happen on E2F1 target genes. How were "potential" E2F-target genes defined? Are these genes dependent on E2F, or are they shared with other transcription factors, such as c-Jun, FOS, or MYC? How many of these genes are specific to E2F1, and how many would be redundant because they could be regulated by other transcription factors?
5) The authors suggest that the RIs are predicted to be capable of forming dsRNAs. What evidence is this prediction based on? Which parameters does the algorithm use to predict such structures? Do these RIs harbor repetitive elements? Do they have any other characteristics that make them prone to forming dsRNA? The authors need to sequence a J2IP, since J2 binds to dsRNA between 14 and 40 bp with increasing affinity for longer dsRNAs.
6) Most predicted sequences will be below the sequence length detected by pattern recognition receptors. The authors should

therefore also define which pattern recognition receptor is activated and what is bound to this receptor.
7) This work suggests that depletion of E2F causes RI events (Figure 1A). However, why do these RIs not result in the accumulation of dsRNA or IFN-β, as observed with PRMT5 inhibition in 1E? The link is not clearly described.
8) While the authors use RNaseIII to control for unspecific staining, they are missing a control for mitochondrial effects. Transcription in mitochondria is bidirectional, resulting in a substantial abundance of dsRNA in these organelles. Furthermore, PRMT5 is known to regulate mitochondrial gene expression1, which renders the increase observed upon PRMT5 treatment difficult to interpret. Therefore, the authors should perform J2 IPs and qPCRs with primers targeting mitochondrial dsRNA or repetitive elements, for instance, and compare the conditions with and without PRMT5 inhibition. Additionally, using Mitotracker in the IF staining could be useful.
9) Figure 3F is hard to understand; it shows 15 blue and 85 red dots. The panels says 16.7 % should be blue and 83.3% red: Is the discrepance meaningful or an error? But the legend says, the analysis was done on 54 genes, so where are those?
10) Also, are the genes in 3F the same as in 3B? So do introns in E2F genes fall into both up and down groups in Figure 3B and, if yes, why?
11) The design of the ChAdOx-GFP control vector is unclear in Figures 4D-G. Is it harboring a scrambled transgene in order to be comparable to the PepMInt transgene? Does the 29-peptide-long PepMInt transgene comprise a GFP tag? This is unclear to this reviewer. If the control construct does not contain a scrambled transgene, why is it a valid control?
12) To strengthen their argument about the role of the immune system and MHC class I prsentation, the authors need to perform the experiments shown in Figures 4D-G in immunocompromised mice or, better, CD8 T cell-depleted mice.

Minor Concerns:
1) Line56: Introduction abbreviation AS should be introduced
2) Line66: ...compared [to] is missing
3) Line 82: "In humans," [comma is missing]
4) Line 102: "in vitro" should be italic
5) Figure 1i: Scale bar is missing
6) Concentration used for T1-44 treatment is missing
7) S4 over 2 pages: split it into two figures; same accounts for S5
8) Line 332: PBMCs; abbreviation is not introduced

1. Huang, L., Liu, J., Zhang, X. O., Sibley, K., Najjar, S. M., Lee, M. M., & Wu, Q. (2018). Inhibition of protein arginine methyltransferase 5 enhances hepatic mitochondrial biogenesis. Journal of Biological Chemistry, 293(28), 10884-10894.

EMM-2025-23176

Responses to the Reviewers from the Authors:

Referee #1 (Comments on Novelty/Model System for Author):

The work is technically strong and novel, combining splicing analysis, immunopeptidomics, vaccination, and human T-cell assays to reveal a new PRMT5-intron retention-immune axis. Medical impact is currently moderate because translation to patients is limited by tumour heterogeneity, HLA restriction, and lack of in vivo human data, although the model systems used are appropriate.

Referee #1 (Remarks for Author):

The manuscript by Blaszczak and colleagues describes a conceptually novel mechanism by which PRMT5 regulates tumour immunogenicity through control of RIs. The authors show that PRMT5 inhibition leads to accumulation of retained introns that form dsRNA structures activating innate interferon responses and encode cryptic ORFs that generate immunogenic peptides presented by MHC I. Using a combination of RNAseq, immunopeptidomics, murine vaccination models, and ex vivo human T cell assays, the study links aberrant splicing to both innate and adaptive immunity. Overall, the work is strong, and of high potential impact, with clear relevance to cancer immunotherapy and PRMT5-targeted strategies, however some mechanistic aspects, interpretations, and translational claims would benefit from further strengthening. Below are my main concerns

Thank you to the Reviewer for his/her important comments on the manuscript and finding our study impactful and novel. The Reviewer raised a number of points, and our responses and revisions are detailed below:

In the first comment, the Reviewer suggested that *while the correlation between PRMT5 inhibition, RI accumulation, dsRNA formation, and IFN-β induction is clear, the manuscript would benefit from stronger causal evidence that RIs are the dominant drivers of the interferon response. For example, selective depletion of specific RI containing transcripts or rescue experiments restoring splicing of key introns could help distinguish RI driven effects from broader splicing disruption caused by PRMT5 inhibition*

In response, we believe that the referee makes an important point. We wonder, however, whether our detailed analysis in Figures 2B–D that retained introns (RIs) form dsRNA structures that drive the interferon (IFN) immune response has been fully appreciated. We believe that in Figure 2B we present data which fulfils the reviewers request, namely establish causal evidence that the RIs derived from our candidate genes increase levels of dsRNA which coincides with elevated IFN levels. Specifically, in 2B we ectopically express either the RI with bordering exons, or bordering exons alone. Expression of the RIs from DDX55 or CDK10 caused a significant increase in dsRNA and IFN, compared to only exons. We believe therefore that RIs can drive the IFN response.

In addition, please be aware that we did not describe RIs as 'dominant drivers of the IFN response', as the referee states above. Rather we suggest that they can via dsRNA formation activate the IFN response, as

can dsRNA structures derived from other sources. We were not suggesting that RIs are the only source of dsRNA.

The experiment proposed by the reviewer, namely measuring the effect of depletion of a specific RI transcript on the dsRNA J2 signal, is unlikely to be successful. While increasing the abundance of even single RI (due to using a highly efficient expression plasmid) can elevate dsRNA levels and induce an IFN response, it is not reasonable to expect that depletion of the expression of a single RI transcript would produce the opposite effect. This is because thousands of introns and other RNA derivatives will contribute to endogenous dsRNA formation, and the IFN response reflects their collective activity.

In the second point, the Reviewer pointed out that *J2 staining is widely used but can detect multiple dsRNA species. It remains unclear to what extent dsRNA signals originate specifically from RIs versus other RNA species. The authors should discuss this limitation more explicitly and if possible provide additional evidence (RNA-FISH for specific RIs) to support RI specificity*

In response, as shown in Figures 2B-D, ectopic expression of individual RI transcripts caused increased levels of dsRNA. We nevertheless acknowledge and agree with the referee that there are limitations to this approach, as PRMT5 inhibition might also alter other sequences to produce dsRNA, detected by the J2 antibody. **In response** we explicitly discussed the limitations of our results with the J2 antibody in the revised manuscript (please see lines 184-188).

In addition, we provided new results in the revised manuscript where we analysed the RNA sequence present in a J2 RNA-IP performed on untreated cells and the impact of PRMT5 inhibition with T1-44, where we identify the CDK10 and DDX55 RIs dsRNA structures characterised in our study (please see Appendix Figure 4K). For the revision, we also included the J2 RIP-seq analysis of publicly available dsRNA RIP-seq datasets and showed that the vast majority of RIs encoding peptide sequences (identified in this study) were enriched in dsRNA immunoprecipitates (please see the Appendix Figure 5G(i-v) and lines 306-311).

Consequently, we can conclude that the detected RIs are recognised by the J2 antibody.

In the third point, the Reviewer commented that *"the mechanism by which the cryptic ORFs are translated remains underexplored. Given the weak Kozak sequences and non-canonical start codons, the authors should discuss alternative initiation mechanisms (leaky scanning, non AUG initiation, ribosome reinitiation) and clarify whether ribosome profiling data or additional evidence could support active translation initiation within introns"*

In response, we wonder whether the referee noticed Figures S5E and S7D in the manuscript, which already shows that RI transcripts are present in translating polysome fractions. In addition, Figure S5C(iii) demonstrates that the majority of predicted open reading frames initiate from alternative start codons.

To further strengthen our conclusions, in the revised manuscript we performed additional polysome profiling RNA-seq, which demonstrated read density across the majority of peptide-coding intronic regions, with many showing increased abundance upon PRMT5 inhibition. This indicates that retained introns are not only accumulated but are actively associated with translating ribosomes (please see Appendix Figure 5F(i-iv)). The polysome profiling RNA-seq data generated in this manuscript have been deposited in the Gene Expression Omnibus (GEO) under accession code: GSE327671 (the following secure token has been created to allow review of record GSE327671 while it remains in private status: ihkryicoptchjyn).

In the next point, the Reviewer suggested that *although RI derived peptides are clearly detectable and immunogenic, their relative abundance compared with canonical tumour antigens is not fully contextualised. Providing estimates of their contribution to the total MHC class I peptidome (percentage of total intensity or peptide counts) would help readers assess their biological and therapeutic relevance*

In response, in the revised manuscript we provided a comprehensive quantitative analysis of the MHC class I peptidome and compared canonical (protein coding genes) and non-canonical (lncRNAs and RIs) peptides (please see the Appendix Figures 5A(ii) and 6A(ii)).

In the next point, the Reviewer commented that *the detection of RI reactive T cells in CRC patients is an important finding, but patient numbers are modest and responses heterogeneous. The authors should temper claims regarding clinical applicability and more clearly discuss inter-patient variability, HLA restriction, and how RI-based vaccines might be personalized or stratified in practice*

In response, we agree that patient numbers are modest (however improved; now 34 Patients after revision) and that human RI-reactive T-cell responses are heterogeneous, which as the reviewer pointed out, is to be expected given inter-patient variability in HLA haplotypes. Nevertheless, we are extremely encouraged by the results which establish that peptide-specific T-cell responses against RI-derived antigens can be detected in human CRC patients.

In response to the referee's comment, in the revised manuscript we added to the discussion the points that the referee has emphasized on inter-patient variability and HLA polymorphism in the human population, and how RI-based vaccine strategies may require patient stratification or personalisation (please see the lines 477-489 in the Discussion).

In the next point, the reviewer pointed out that *some phenotypes observed upon PRMT5 inhibition (interferon activation, increased MHC class I expression) may also arise from general splicing perturbation. A more explicit comparison between PRMT5 inhibition and other splicing perturbations (beyond pladienolide as a control) would help clarify whether PRMT5 has a unique role or primarily acts as a gateway to splicing stress induced immunogenicity*

In response, we agree with the referee that some phenotypes observed upon PRMT5 inhibition, including interferon activation and increased MHC class I expression, may also result from more general splicing perturbations. However, PRMT5 is a well-established regulator of RNA splicing, with multiple methylation-dependent splicing targets involved in splicing fidelity [1]. Therefore, the effects observed following PRMT5 inhibition are consistent with its central role in regulating splicing programmes.

We analysed publicly available RNA-seq datasets from cells depleted of major splicing regulators methylated by PRMT5 (downstream targets of PRMT5). Knockdown of SRSF2 (RNA-seq data, accessible in GEO under accession codes GSE207643 [2]), a positive splicing regulator, led to increased retained intron events, largely upregulated, mirroring the effect observed upon PRMT5 inhibition (Figure for the Reviewer 1A(i-iv)). Also, similarly to the PMRT5 inhibition effect, gene expression and GO analysis revealed activation of type I interferon signalling, antigen presentation, and RNA splicing pathways (Figure for the Reviewer 1A(v)). In contrast, hnRNPA1 knockdown (RNA-seq data, accessible in GEO under accession code GSE267007 [3]), a splicing repressor, resulted in altered splicing with most RIs being spliced out (Figure for the Reviewer 1B(i-iv)). Despite this opposing effect on RI abundance, hnRNPA1 depletion similarly induced type I interferon and antigen presentation gene signatures (Figure for the Reviewer 1B(v)). Together, these findings suggest that perturbation of splicing homeostasis can contribute to activation of immune signalling programmes. However, both SRSF2 and hnRNPA1 are established methylation targets

for PRMT5, meaning that the splicing effect of PRMT5 inhibition might be mediated through either of these two downstream targets.

We have not included these new results in the revision, but we are happy to do so if the Reviewer feels it would be worthwhile.

[1] Wu, Q., Schapira, M., Arrowsmith, C.H. et al. Protein arginine methylation: from enigmatic functions to therapeutic targeting. Nat Rev Drug Discov 20, 509–530 (2021).

[2] Ma HL, Bizet M, Soares Da Costa C, Murisier F, de Bony EJ, Wang MK, Yoshimi A, Lin KT, Riching KM, Wang X et al (2023) SRSF2 plays an unexpected role as reader of m(5)C on mRNA, linking epitranscriptomics to cancer. Mol Cell 83: 4239-4254 e4210

[3] Lin L, Huang X, Huang C, Zou J, Hu M, Lan T, Wang H, Du Z, Tian M, Liu J et al (2025) Inflammatory signaling attenuates spliceosome function and cognitive ability. Autophagy 21: 3123-3141

Figure for reviewers removed

Minor comments:

The manuscript alternates between "RI", "RIs", and "retained introns" frequently. A brief definitional reminder early in the Results and consistent usage thereafter would improve readability, especially for non-splicing specialists.

In response, the definition of the Retained Intron early in the Results section was added.

Many thanks to the Reviewer for their insightful comments. We hope that the Reviewer now finds the manuscript acceptable for publication.

EMM-2025-23176

Responses to the Reviewers from the Authors:

Referee #2 (Remarks for Author):

In this paper Blaszczak and colleagues identify a role for PRMT5 mediated intron retention in innate and adaptive immune responses in colorectal cancer. They find that PRMT5 inhibition leads to extensive intron retention and these introns are the source of IFN activating dsRNAs and neo-antigens. They further map these neo-antigens and generate a vaccine based on these peptides which has some efficacy in a mouse cancer model. They also find evidence of immune reactivity towards these neo-antigens in T cells from CRC patients. Together, this is an interesting study and with growing interest in activating immune responses in CRC, it is timely with potential implications for anti-cancer therapy. However, there are a number of issues that need to be addressed prior to publication.

Thank you to the Reviewer for his/her important comments on the manuscript and finding our study interesting. The Reviewer raised a number of points, and our responses and revisions are detailed below:

Main points:

In the first point, the Reviewer found *the IHC in Figure 2A is unconvincing.* They suggested that *the authors need to control for background staining with RNAse treatment. Also, what is the model that is used and the treatment regime, I couldn't find the details of this in the paper? Further, what are the phenotypic effects of this treatment and proposed dsRNA accumulation on tumour growth, apoptosis, proliferation etc?*

In response, we agree that appropriate controls are important for interpretation of the IHC data in Figure 2A and we were remiss in not including the requested control that the referee has requested.

It is worth mentioning that in the experimental setting shown in Figure 2A, inclusion of an RNase III treated negative control for IHC proved technically challenging due to the use of multilayer tumour sections, which limit efficient RNase III penetration and result in incomplete digestion of dsRNA in FFPE samples from T1-44 treated mice. In contrast, RNase III-dependent loss of J2 binding is readily observed in single cell-based assays, including flow cytometry and monolayer immunofluorescence, which are presented in Fig.1F and Fig.2C and provide independent validation of dsRNA specificity.

However, in the revised manuscript, we improved our procedures and included the RNAse III treatment control for the tumour tissue IHC, as requested by the referee (please see Appendix Figure 4I). In the control group, the signal was almost completely abolished following RNase III treatment. In the T1-44-treated group, a small residual signal remained detectable (Figure 4I); however, it was substantially reduced compared with the signal observed in Figure 2A(i).

With respect to the in vivo tumour model and treatment regimen, we note that these details were described previously and are explicitly referenced in the manuscript (please see lines 210-214 and 705-706). The mouse experiments, including treatment conditions and phenotypic outcomes, were reported in detail in Barczak et al. (2023).

In the revised manuscript, we improved the text describing the model and therapeutic effects of T1-44 treatment, and referenced the Barczak et al. (2023) publication more transparently.

In the second point, the Reviewer asked that *in Figure 2B how plausible is it that a single RI is detectable by the J2 dsRNA antibody and can lead to IFN activation. Is this a generic activation with any RI or specific to the ones induced by PRMT5 inhibition? Can the authors investigate the effects of other RNA sequences, including those predicted to form dsRNA by the ViennaRNA algorithm and those not.*

In response, we agree with the reviewer that any single RI expressed at endogenous levels is not likely to produce detectable dsRNA or IFN activation. In these results shown in Figure 2B however, the RIs were expressed ectopically under the strong CMV promoter which provides a high level of expression.

In addition, we do not propose that RIs induced by PRMT5 inhibition have a unique capacity to form dsRNA or activate innate immunity; rather, we believe this property is shared by RI transcripts in general, irrespective of how they are regulated, and is well-established in the literature.

In the revised manuscript, and in the interests of providing clarity for the referee, we cited additional literature which document other types of dsRNA structures as activators of IFN (please see Discussion lines 444-452).

In the third point, the Reviewer suggested that *to enhance the findings in Figure 2, can the authors pulldown dsRNA using J2 in their PRMT5 inhibitor treated cells to determine whether the DDX55 and CDK10 RIs can be detected using this method in cells without ectopic overexpression?*

In response, we agree with the reviewer that assessing dsRNA formation by endogenous RIs would further strengthen Figure 2.

To address this point, for the revised manuscript we performed J2 RNA immunoprecipitation (RIP) in PRMT5 inhibitor treated cells and detected endogenous CDK10 and DD55 intronic RNA, confirming that RI-containing transcripts (identified in this study) form dsRNA structures in cells (Appendix Figure 4K, please see lines 233-235).

In the fourth point, the reviewer pointed out that *in Figure 3B there are almost as many downregulated peptides identified as upregulated. What do the authors propose is the reason for this?*

In response, we agree that both upregulated and downregulated peptides are observed in Figure 3B. This is expected because PRMT5 inhibition broadly affects transcription, RNA splicing, and antigen presentation pathways. As a result, some RI-derived peptides become more abundant, while others decrease due to altered transcript expression and splicing patterns. Therefore, PRMT5 inhibition reshapes the overall antigen presentation landscape rather than simply increasing peptide presentation.

In the revision, we cited relevant studies (including Barczak et al. 2023) demonstrating that other treatments reshape, rather than uniformly increase, peptide presentation, and incorporated this into the Discussion and cited appropriate literature in the revised manuscript (please see Discussion lines 466-471).

In the next point, the Reviewer commented that *in Figure 4E-F, what are the sequences that elicit the strongest immune responses in the mouse ELISAspots?*

In response, the peptide sequences that elicit the strongest immune responses in the mouse ELISpot assays are provided in the Appendix Figures. The response magnitudes are shown in Figure S9, and the corresponding peptide sequences are listed in Figure S8A.

To improve clarity for the reader, in the revised manuscript we relabelled the ELISpot graphs in Figures S9 to display peptide sequences (rather than peptide numbers).

In the next point, the Reviewer commented that *tumour vaccine data is interesting but the level of tumour control is modest. This could be because the neo-antigens used for this vaccine were predominantly identified PRMT5 inhibition and therefore basal expression of them is low. Could the authors combine the vaccination with PRMT5 inhibitor treatment to determine if this increases efficacy?*

In response, these RI-derived antigens comprising the vaccine cargo are bidirectionally regulated (namely up- and down-regulated) upon treatment with the inhibitor, from a basal constitutive level of expression. Out of 29 peptides within the vaccine, 15 are induced by PRMT5 inhibition while remaining 14 are decreased (as shown in Appendix Figure 8).

Nevertheless, following Reviewer's suggestion, in the revised manuscript we evaluated whether combining PepMInt with PRMT5 inhibition (T1-44) could further enhance anti-tumour efficacy. However, the combination treatment did not result in improved tumour control compared to PepMInt alone, indicating no additive or synergistic benefit under these conditions. One possible explanation is that PRMT5 inhibition itself promotes the generation and presentation of retained intron (RI)-derived peptides, thereby reshaping the tumour antigen landscape in a manner that reflects the antigenic cargo in the PepMInt vaccine. As a result, both agents may induce overlapping immune responses against shared RI-derived epitopes, limiting the consequence of combining the vaccine with T1-44 treatment. These findings suggest that PRMT5 inhibition may function, at least in part, as an inducer of RI-derived tumour antigens, leading to immune effects comparable to those achieved by vaccination alone (Appendix Fig. S10D-F; please see lines 375-382).

In the next point, the Reviewer asked if *in Figure 5B are the effects peptide specific or simply due to T cell activation? For example, what is the effect of co-culturing PHA stimulated T cells on HCT116 growth? Also, does PRMT5 inhibitor treatment enhance the effect of T cell killing?*

In response, in Figure 5Bii and Appendix Figure 11D-E, T cells were already expanded with PHA treatment prior to the target cell killing assay to obtain a sufficient number of effector T cells. The observed effects reflect comparisons between peptide-pulsed and control targets, indicating peptide-specific killing rather than non-specific T cell activation.

In response to the referee's suggested experiment, we also assessed whether PRMT5 inhibition enhances T cell mediated killing by performing killing assays using T1-44 pre-treated HCT116 target cells. Peripheral blood mononuclear cells (PBMCs) and isolated T cells from three independent patients were co-cultured with T1-44-pre-treated or untreated target cells. Increased killing (of 12%) following T1-44 pre-treatment was observed only in one case (T cells, Patient #32), whereas in the remaining samples no statistically significant enhancement of cytotoxicity was detected. These results suggest that PRMT5 inhibition may augment T cell-mediated killing in a subset of individuals, but the effect appears to be variable and patient-dependent (Figure 1A-C for the Reviewer 2). We will continue this analysis in future experiments to achieve increased patient number,

Figure for reviewers removed

In the last point, the Reviewer pointed out that *the human RI data is confusing and counterintuitive. If the RI is leading to neo-antigen production that is recognised by T cells it would be expected to correlate with better survival due to increased immune control. Can the authors expand on this in the discussion?*

In response, we agree that the relationship between RI levels, antigen expression, and clinical outcome is complex and on face value appears to be counterintuitive. Although we identified T cell responses against RI-derived peptides in CRC patients, higher RI transcript expression was unexpectedly associated with poorer survival. This may reflect the highly immunosuppressive tumour microenvironment in aggressive tumours, where mechanisms such as PD-1-mediated T cell exhaustion and extracellular acidosis impair

effective anti-tumour immunity despite the presence of immunogenic antigens. Thus, although RI-derived peptides may naturally elicit T cell responses, these responses are likely insufficient for tumour control without therapeutic immune activation, supporting the rationale for vaccine-based approaches (please see Discussion; lines 490-495).

Many thanks to the Reviewer for their insightful comments. We hope that the Reviewer now finds the manuscript acceptable for publication.

16th May 2026

EMM-2025-23176

Responses to the Reviewers from the Authors:

Referee #3 (Remarks for Author):

In this study, the authors suggest that the arginine-methyltransferase PRMT5 is involved in coordinating innate and adaptive immune responses. The authors applied alternative splicing analyses to RNA-seq data and found that both PRMT5 and E2F1 regulate RNA splicing. Earlier research has shown that E2F1 is methylated by PRMT5, which enables its involvement in alternative splicing. If absent, intron retention is increased, and these regions have been shown to have a high propensity to form immunogenic dsRNA, activating innate immune signaling. Furthermore, they identified small peptides of RIs on the MHC class I complex, indicating the involvement of both innate and adaptive immunity. Following these findings, an RI-derived transgene was tested in animal experiments. In addition, T lymphocytes from human cancer patients demonstrated antigen specificity for RI-derived peptides, resulting in the killing of human tumor cells in vitro.
While the work is conceptually interesting, the mechanisms underlying the proposed model lack in-depth evaluation. Furthermore, while the effects of PRMT5 are comprehensively documented, the link to E2F1 is not as well described and remains rather elusive. Furthermore, the in vivo data require clarification regarding their design, and additional experiments are needed to evaluate the role of the immune system.

Thank you to the Reviewer for his/her important comments on the manuscript and finding our study interesting. The Reviewer raised a number of points, and our responses and revisions are detailed below:

Major Concerns:

In the first point, the Reviewer pointed out that the *Figure 1A shows a heavily derivatized plot of RI data "normalized PSI values" without clear indication how data were normalized or what the cut-off is. Why do data need to be normalized? PSI is a percentage value, so absolute values can be used for the analysis. Also, the authors should show the browser pictures for 2-3 of the strongest effects.*

In response, we appreciate the reviewer's request for clarification. The normalisation of PSI values in Figure 1A was necessary to highlight relative changes within individual retained introns across the conditions. While PSI values are percentages, baseline PSI levels vary widely between different RIs. As a result, plotting absolute PSI values would obscure treatment-related changes in RIs with low or intermediate baseline inclusion and consequently bias the visualization toward introns with intrinsically high PSI. Normalisation therefore enables a more informative comparison of relative RI modulation across the dataset. However, to improve the interpretation of the data, in the revised manuscript we added Figure 1A(ii) (and also Appendix Figure 2E(ii)), showing differentially upregulated and downregulated RI events in different conditions presented as a delta PSI plot. We have also included the representative Sashimi plots in the Appendix Figure S1B.

In the revision, we also updated the Material and Methods section (please see Heatmap generation and normalisation section in Material and Methods; please see lines 600-608).

In the second point, the Reviewer commented that *as illustrated in Figure 1A (ii), the overlap of the Venn diagram is approximately 9% between the E2F1Cr and E2F1Cr/T1-44 condition. Could the authors please comment on these figures? If these RIs occur due to an interplay between E2F and PRMT5, should the overlap not be higher? What is the mechanistic explanation for that?*

In response, the small overlap between E2F1Cr and E2F1Cr/T1-44 conditions reflects the fact that E2F1 and PRMT5 contribute to RI regulation through both shared and independent mechanisms, rather than acting as a single linear pathway, as E2F1 is one of many and not an exclusive PRMT5 target. PRMT5 broadly affects spliceosome assembly, leading to widespread splicing alterations that are not exclusively E2F1-dependent. Conversely, E2F1 primarily impacts a subset of introns associated with E2F1-regulated transcriptional programmes and chromatin context [1, 2]. Importantly, the shared subset likely represents introns that are co-regulated by E2F1-dependent transcriptional control and PRMT5 (through its ability to methylate E2F1 [3]). Whereas the non-overlapping events reflect condition-specific regulatory inputs, meaning that certain introns are influenced uniquely by PRMT5-dependent alterations in spliceosome activity or, alternatively, by E2F1-driven changes without requiring simultaneous contribution from both factors.

In the revised manuscript, we clarified our explanation for the minimal overlap which we suggest does not argue against functional interplay, but rather supports a model in which E2F1 and PRMT5 exert partially overlapping in addition to mechanistically distinct effects on intron retention.

[1] Carr, S. M., Liu, G., Barczak, W., Syffudin, H., Munro, S., Reyna-Jeldes, D., Vendrell, I., Kessler, B., Cribbs, A. P., Kanapin, A., Samsonova, A., & La Thangue, N. B. (2026). Separate transcription and splicing gene networks are linked and coordinated by the pRb-E2F pathway. Nucleic acids research, 54(3), gkag016.

[2] Roworth, A. P., Carr, S. M., Liu, G., Barczak, W., Miller, R. L., Munro, S., Kanapin, A., Samsonova, A., & La Thangue, N. B. (2019). Arginine methylation expands the regulatory mechanisms and extends the genomic landscape under E2F control. Science advances, 5(6), eaaw4640.

[3] Cho, E. C., Zheng, S., Munro, S., Liu, G., Carr, S. M., Moehlenbrink, J., Lu, Y. C., Stimson, L., Khan, O., Konietzny, R., McGouran, J., Coutts, A. S., Kessler, B., Kerr, D. J., & Thangue, N. B. (2012). Arginine methylation controls growth regulation by E2F-1. The EMBO journal, 31(7), 1785–1797.

In the third point, the Reviewer commented that *the authors claim in line 129 that RI events depend on E2F1 (and PRMT5). However, using the parameters in Figure S1B(iii) (10% PSI cutoff), the total number of RI events does not increase with E2F1 depletion. What would the bar chart look like with the parameters used in Figure 1? I acknowledge that Figure S1A displays a similar plot, but it would be clearer if the control was placed next to the other conditions instead of using a p-value as the cutoff.*

In response, the referee makes an important point. Figure 1A and Figure S1C use different analysis criteria, which explains the perceived discrepancy highlighted by the referee. Specifically, Figure 1A reports differential splicing events (each event is tested and reported relative to the control treatment (WT)), so the numbers reflect statistically significant changes compared to WT. Figure S1C, in contrast, displays raw PSI values after applying a 10% PSI cutoff to remove low-abundance retained introns. It therefore represents the number of events over an abundance threshold rather than a number of events identified as differentially regulated. Because E2F1 knockout changes which introns are affected (rather than simply producing a larger total number of retained introns add figure here), the two plots would not be expected to overlap exactly. Figure S1C already reports the number and percentage of differentially spliced events (i.e. compared to control), so the control is implicitly included in that comparison.

In the revision, we updated the figure legends and Material and Methods (please see please see Heatmap generation and normalisation section; please see the lines 600-608) so that the different types of analysis are explicitly described.

In the fourth point, the Reviewer pointed out that *in Figure 1B, the authors suggest that these RI events primarily happen on E2F1 target genes. How were "potential" E2F-target genes defined? Are these genes dependent on E2F, or are they shared with other transcription factors, such as c-Jun, FOS, or MYC? How many of these genes are specific to E2F1, and how many would be redundant because they could be regulated by other transcription factors?*

In response to the referee's question, the definition of potential E2F1 target genes is described in the ENCODE ChIP-seq data analysis section in Material and Methods (ENCODE ChIP-seq data analysis; line 507)

We agree with the referee that many of the E2F target genes may also be co-regulated by other transcription factors such as MYC, c-Jun or FOS, and that the presence of E2F binding sites in the promoter region does not necessarily imply unique regulation by the E2F pathway. However, our conclusion in Figure 1B is that RI events are enriched in genes bound by E2F1, not that they are exclusively regulated by E2F1. Other transcription factors could well contribute to the transcriptional regulation of these genes in cells, which is an interesting question that we will evaluate in future experiments.

In the next point, the Reviewer commented that *the authors suggest that the RIs are predicted to be capable of forming dsRNAs. What evidence is this prediction based on? Which parameters does the algorithm use to predict such structures? Do these RIs harbor repetitive elements? Do they have any other characteristics that make them prone to forming dsRNA? The authors need to sequence a J2IP, since J2 binds to dsRNA between 14 and 40 bp with increasing affinity for longer dsRNAs.*

In response, the prediction for the number of dsRNA structures (shown in Figure 1D) was generated using the ViennaRNA Package RNA-fold tool, as described in the Materials and Methods (dsRNA prediction; line 738), and was further supported by the experimental validation in Figures 2B–D.

In the revised manuscript, we included an additional analysis on predicted dsRNA, as previously described (e.g., [1] Trotta *et al.*), to compare randomly generated RNA sequence with the prediction in Figure 1D on dsRNA formed with RIs. We estimated the prevalence and predicted stability of structures within the retained introns using AMFE (adjusted minimum free energy; minimum folding free energy of an RNA sequence normalised to a standard length, typically per 100 nucleotides, allowing structural stability comparisons between RNAs of different sizes). These results (Appendix Fig. S4F) show that the AMFE values of the RIs identified in our study are significantly lower, indicating the formation of more stable dsRNA structures, compared with randomly shuffled RNA sequences of similar lengths (12–600 nt), which have an average AMFE of ~ −32 kcal/mol (Trotta et al, 2014). Notably, the lowest AMFE values were observed among RIs upregulated following T1-44 treatment. These results suggest that the secondary structures originating from RIs are more stable than random sequences (described in Trotta et al) and thus they may contribute to the pool of dsRNAs we observe in T1-44 treated cells (please see Appendix Figure S4F and lines 171-181).

For the revision, we also included the J2 RIP-seq analysis as requested by the referee, which revealed that the vast majority of RIs encoding peptide sequences (identified in this study) were enriched in dsRNA immunoprecipitates (please see the Appendix Fig. S5G(i-v) and lines 306-311).

[1] Trotta E. On the normalization of the minimum free energy of RNAs by sequence length. PLoS One. 2014 Nov 18;9(11):e113380. doi: 10.1371/journal.pone.0113380. PMID: 25405875; PMCID: PMC4236180.

In the next point, the Reviewer pointed out that *most predicted sequences will be below the sequence length detected by pattern recognition receptors. The authors should therefore also define which pattern recognition receptor is activated and what is bound to this receptor.*

In response, we agree with the referee that many predicted RNA structures fall below the minimum length typically associated with activation of pattern recognition receptors. However, our analyses also identified a substantial number of double-stranded RNA regions exceeding ~15 bp, which are within the reported recognition range of receptors such as RIG-I and PKR [1,2]. We therefore consider it plausible that these longer dsRNA structures contribute to innate immune activation.

To further clarify this issue, in the revised manuscript we included new data relating to the role of the dsRNA sensor PKR in the observed effects on IFN activation (please see Appendix Figure S4H and lines 199-205). To confirm that dsRNA recognition pathways contribute to innate immune activation, we treated cells with a siRNA against PKR and we observed that it is only when cells have active PKR does IFN-β become elevated upon T1-44 treatment, which is consistent with a linear signalling pathway from dsRNA.

[1] Cottrell KA, Andrews RJ, Bass BL. The competitive landscape of the dsRNA world. Mol Cell. 2024 Jan 4;84(1):107-119. doi: 10.1016/j.molcel.2023.11.033. Epub 2023 Dec 19. PMID: 38118451; PMCID: PMC10843539.

[2] Zheng X, Bevilacqua PC. Activation of the protein kinase PKR by short double-stranded RNAs with single-stranded tails. RNA. 2004 Dec;10(12):1934-45. doi: 10.1261/rna.7150804. PMID: 15547138; PMCID: PMC1370682.

In the next point, the Reviewer commented that *this work suggests that depletion of E2F causes RI events (Figure 1A). However, why do these RIs not result in the accumulation of dsRNA or IFN-β, as observed with PRMT5 inhibition in 1E? The link is not clearly described.*

We thank the referee for this question. **In response**, it is correct that loss of E2F1 alone increases intron retention, as shown in Fig. 1A, but this does not result in detectable dsRNA accumulation or induction of an IFN response. We expect that this lack of effect might be due to additional effects of PRMT5 inhibition which impact quantitatively on RIs, such as RNA stability and turnover. This notion is supported by the analysis of predicted stability by Adjusted Minimum Free Energy (Appendix Figure S4F), which indicates dsRNA structures identified within T1-44 treated cells as the most stable out of all tested conditions. In the revised manuscript, we described this idea as a possible explanation (please see lines 171-181).

In the next point, the Reviewer commented that *while the authors use RNaseIII to control for unspecific staining, they are missing a control for mitochondrial effects. Transcription in mitochondria is bidirectional, resulting in a substantial abundance of dsRNA in these organelles. Furthermore, PRMT5 is known to regulate mitochondrial gene expression1, which renders the increase observed upon PRMT5 treatment difficult to interpret. Therefore, the authors should perform J2 IPs and qPCRs with primers targeting mitochondrial dsRNA or repetitive elements, for instance, and compare the conditions with and without PRMT5 inhibition. Additionally, using Mitotracker in the IF staining could be useful.*

Many thanks for the referee's important comment. **In response**, it should be noted that we did not detect any mitochondria-derived transcripts or peptides in our analyses (including RNAseq, immunopeptidome,

polysome profiling). Further, human mitochondrial genes generally lack introns [1]; therefore, we consider it unlikely that mitochondrial transcription will produce RIs that subsequently give rise to dsRNA structures. However, we agree the referee that the IHC performed on cells with J2 could, theoretically, identify dsRNA (derived from other sources) in mitochondria although, given the absence of detectable mitochondrial sequences in our studies, would seem to be relatively unlikely.

[1] Rackham, O., Filipovska, A. Organization and expression of the mammalian mitochondrial genome. Nat Rev Genet 23, 606–623 (2022).

In the next point, the Reviewer pointed out that *Figure 3F is hard to understand; it shows 15 blue and 85 red dots. The panels say 16.7 % should be blue and 83.3% red: Is the discrepance meaningful or an error? But the legend says, the analysis was done on 54 genes, so where are those?*

In response, we thank the referee for carefully examining Figure 3F and for spotting our error. The discrepancy between the number of dots shown and the percentages indicated in the panel, as well as the reference to 54 genes in the legend, was due to our error in the original figure and legend. This has now been corrected in the revised version of Figure 3F, and the figure, percentages, and legend are now fully consistent.

In the next point, the Reviewer asked if there *are the genes in 3F the same as in 3B? So do introns in E2F genes fall into both up and down groups in Figure 3B and, if yes, why?*

We thank the referee for this important question. **In response**, the measurements shown in Figures 3B and 3F are not the same. Figure 3B displays peptides, whereas Figure 3F summarizes the genes from which these peptides originate. Therefore, Figure 3B reflects peptide-level regulation, while Figure 3F represents gene-level annotation.

Importantly, the peptides in Figure 3B are regulated in both directions (namely up and down) upon PRMT5 inhibition, independently of E2F1 regulation. Consequently, the presence of genes in Figure 3F does not imply that introns in E2F target genes fall into both up- and down-regulated groups in Figure 3B. Rather, the changes observed in Figure 3B, where some peptides increase and others decrease upon PRMT5 inhibition, reflect PRMT5-dependent effects at the peptide level and are independent of E2F1-mediated regulation.

In the next point, the Reviewer commented that the design of the ChAdOx-GFP control vector is unclear in Figures 4D-G. Is it harboring a scrambled transgene in order to be comparable to the PepMInt transgene? Does the 29-peptide-long PepMInt transgene comprise a GFP tag? This is unclear to this reviewer. If the control construct does not contain a scrambled transgene, why is it a valid control?

In response, ChAdOx1-GFP was used as a vector- and expression-matched control to account for innate immune activation and non-specific effects of adenoviral transduction and transgene expression. GFP provides robust transgene expression without sharing sequence homology or immunological relevance to the RI antigens of interest, allowing RI antigen-specific effects to be attributed to the inserted cargo sequence.

In contrast, scrambled transgene controls, suggested by the referee, may generate unintended MHC-binding epitopes and confound interpretation.

Use of ChAdOx1-GFP as a negative control is standard practice in ChAdOx1-based vaccine studies and is widely used in the literature [1, 2, 3].

[1] Nazki, S., Reiné, J., Kailath, R., Gilbert, S., & Douradinha, B. (2026). Replication-incompetent viral vaccine vectors ChAdOx1 and MVA as tools for evaluating T-cell responses to naturally processed antigens in vitro. Virus research, 364, 199691.

[2] Watanabe, Y., Mendonça, L., Allen, E. R., Howe, A., Lee, M., Allen, J. D., Chawla, H., Pulido, D., Donnellan, F., Davies, H., Ulaszewska, M., Belij-Rammerstorfer, S., Morris, S., Krebs, A. S., Dejnirattisai, W., Mongkolsapaya, J., Supasa, P., Screaton, G. R., Green, C. M., Lambe, T., … Crispin, M. (2021). Native-like SARS-CoV-2 spike glycoprotein expressed by ChAdOx1 nCoV-19/AZD1222 vaccine. ACS Cent. Sci. 2021, 7, 4, 594–602

[3] Vardeu, A., Davis, C., McDonald, I., Stahlberg, G., Thapa, B., Piotrowska, K., Marshall, M. A., Evans, T., Wheeler, V., Sebastian, S., & Anderson, K. (2022). Intravenous administration of viral vectors expressing prostate cancer antigens enhances the magnitude and functionality of CD8+ T cell responses. Journal for immunotherapy of cancer, 10(11), e005398.

In the next point, the Reviewer suggested that *to strengthen their argument about the role of the immune system and MHC class I presentation, the authors need to perform the experiments shown in Figures 4D-G in immunocompromised mice or, better, CD8 T cell-depleted mice.*

We thank the reviewer for this suggestion. We respectfully note that the experiments proposed (immunocompromised or CD8 T cell-depleted mice) would primarily address whether the observed tumour control is mediated through $CD8^+$ T cells. Our immunopeptidome was performed on peptides associated with MHC class I, which is specially recognised by the CD8 cell T cell receptor, and peptides derived from this methodology have been extensively validated in ours (Figures 3-5, Appendix Figures S9-S11) and other studies as targets for CD8 cells [1, 2, 3]. With respect to the referee, it seems therefore to be somewhat redundant to repeat all of our mouse studies in immunocompromised or CD8 depleted mice, given the focus of the experiments on CD8 T cells already described in the manuscript.

Importantly, in the present manuscript we also demonstrate that the identified human MHC class I- restricted peptides bind $CD8^+$ T cells and induce their activation (Fig. 5A), further supporting the $CD8^+$ T cell-dependent mechanism.

1. Barczak, W., Carr, S. M., Liu, G., Munro, S., Nicastri, A., Lee, L. N., Hutchings, C., Ternette, N., Klenerman, P., Kanapin, A., Samsonova, A., & La Thangue, N. B. (2023). Long non-coding RNA-derived peptides are immunogenic and drive a potent anti-tumour response. Nature communications, 14(1), 1078. https://doi.org/10.1038/s41467-023-36826-0
2. Bassani-Sternberg, M., Bräunlein, E., Klar, R., Engleitner, T., Sinitcyn, P., Audehm, S., Straub, M., Weber, J., Slotta-Huspenina, J., Specht, K., Martignoni, M. E., Werner, A., Hein, R., H Busch, D., Peschel, C., Rad, R., Cox, J., Mann, M., & Krackhardt, A. M. (2016). Direct identification of clinically relevant neoepitopes presented on native human melanoma tissue by mass spectrometry. Nature communications, 7, 13404. https://doi.org/10.1038/ncomms13404
3. Zhang, B., & Bassani-Sternberg, M. (2023). Current perspectives on mass spectrometry-based immunopeptidomics: the computational angle to tumor antigen discovery. Journal for immunotherapy of cancer, 11(10), e007073. https://doi.org/10.1136/jitc-2023-007073

Minor Concerns:

1) Line56: Introduction abbreviation AS should be introduced

Introduced line 45

2) Line66: ...compared [to] is missing

Corrected

3) Line 82: "In humans," [comma is missing]

Added

4) Line 102: "in vitro" should be italic

Corrected

5) Figure 1i: Scale bar is missing

Corrected

6) Concentration used for T1-44 treatment is missing

Concentration added to the Material and Methods (please see Cell line section)

7) S4 over 2 pages: split it into two figures; same accounts for S5

Structure of the figures will be discussed with the Editor

8) Line 332: PBMCs; abbreviation is not introduced

Abbreviation explained

In response, we corrected all Minor points raised by the Reviewer.

Many thanks to the Reviewer for their insightful comments. We hope that the Reviewer now finds the manuscript acceptable for publication.

10th Jul 2026

Dear Prof. La Thangue,

Thank you for submitting your revised manuscript, and please accept my apologies for the delay in getting back to you while we awaited the remaining referee report.

We have now received the final report. As you will see below, referees #1 and #2 are satisfied with the revisions overall, and referee #3 also finds the study mechanistically interesting and considers that you have responded well to the previous comments.
I am pleased to inform you that I will be able to accept your manuscript once a few remaining points, together with the editorial requests below, have been addressed.

The remaining concerns relate primarily to the interpretation of the mechanistic findings. In particular, the manuscript would benefit from a more cautious presentation of the proposed link between PRMT5-mediated intron retention and dsRNA accumulation, given the relatively modest effects of PRMT5 inhibition on splicing. Please therefore temper the mechanistic conclusions by clarifying that E2F1 contributes to intron retention, whereas PRMT5 is likely to influence the interferon response through additional mechanisms, and by acknowledging that the J2 antibody may detect dsRNA species other than those arising from retained introns.

Regarding the vaccination experiments, please also state more explicitly that, although the data strongly support a role for $CD8^+$ T cells, definitive in vivo evidence for CD8 dependence would require depletion experiments.

Additionally, please address the editorial points below:
1/ Manuscript text:
- Please remove the red font text and only keep in track changes mode any new modification.
- Correct the order and headings of the manuscript sections to: Abstract, Introduction, The Paper Explained, Results, Discussion, Methods, Acknowledgements, Disclosure and competing interests statement, References, Figure legends, Expanded View Figure legends
- In the Methods (remove Methods and Protocols):
o Animals: provide a statement of compliance with ethical regulations
o Human participants: Include a statement confirming that the experiments conformed to the principles set out in the WMA Declaration of Helsinki and the Department of Health and Human Services Belmont Report. Provide reference number for ethics approval if applicable.
o Statistics: provide a statement on inclusion/exclusion criteria.
- Data Availability: please only list here datasets newly generated as part of this study. All remaining text should be removed.
- BIORENDER: remove from Fig 5 legend and add a dedicated paragraph to the Methods, using this format:
Graphics:
(some of the... OR Figure #... OR synopsis) Graphics were created with BioRender.com.
- 'Ethics declarations' should be renamed 'Disclosure and competing interests statement'
- Remove Authors contribution and provide CRediT (Contributor Role Taxonomy) terms in the submission system instead. These replace a narrative author contribution section in the manuscript.

2/ Figures:
- We encourage you to make some of your Appendix figures into Expanded View (EV) Figures and Tables that are collapsible/expandable online. EV Figures should be cited as 'Figure EV1, Figure EV2" etc... in the text and their respective legends should be included in the main text after the legends of regular figures.
For the figures that you do NOT wish to display as Expanded View figures, they should be bundled together with their legends in a single PDF file called *Appendix*, which should start with a short Table of Content. Appendix figures should be referred to in the main text as: "Appendix Figure S1, Appendix Figure S2" etc. Please remove the EV dataset legends from the appendix file and add them to each corresponding dataset, in a separate worksheet. Please also remove the EV table legend and add it to top of the table. Add page numbers to the appendix PDF and to its table of contents with the remaining appendix figures.
- During our standard figure checks, similarities were found between Figure 4F and Appendix Fig S9C, please check and correct if needed. Please note that figure re-use is allowed, but should be mentioned in the figure legend.
- Please make sure that all your figures and figure panels are referenced in the text. Currently, a callout is missing for Figure 5C.
- Please address the queries from our data editors in the figure legends:
1. Please note that the exact p values are not provided in the legends of 1E,F(iii); 2A(i)(ii), 2B(i)(ii)(v), 2C(ii), 2D; 4G(vi); 5B(i)(ii).
2. Please carefully check the legend for Figure 1. Some graphs are incorrectly described as box-and-whisker plots. Please revise these descriptions and clearly define what the central line and error bars represent.

3/ Author checklist:
Please fill in:

- Experimental study design and statistic, inclusion/exclusion criteria
- Ethics, informed consent and Helsinki declaration

4/ Source Data:
For Western blots, please provide if annotated uncropped original pictures.

5/ Please provide 'The paper explained': EMBO Molecular Medicine articles are accompanied by a summary of the articles to emphasize the major findings in the paper and their medical implications for the non-specialist reader. Please provide a draft summary of your article highlighting
- the medical issue you are addressing,
- the results obtained and
- their clinical impact.

This may be edited to ensure that readers understand the significance and context of the research. Please refer to any of our published articles for an example.

6/ Every published paper includes a 'Synopsis' to further enhance discoverability. Synopses are displayed on the journal webpage and are freely accessible to all readers. They include a short stand first (maximum of 300 characters, including space) as well as 2-5 one-sentences bullet points that summarizes the paper. Please write the bullet points to summarize the key NEW findings. They should be designed to be complementary to the abstract - i.e. not repeat the same text. We encourage inclusion of key acronyms and quantitative information (maximum of 30 words / bullet point). Please use the passive voice. Please attach these in a separate file or send them by email, we will incorporate them accordingly.

Please also suggest a striking image or visual abstract to illustrate your article as a PNG file 550 px wide x 300 px high.

7/ As part of the EMBO Publications transparent editorial process initiative (see our Editorial at http://embomolmed.embopress.org/content/2/9/329), EMBO Molecular Medicine will publish online a Review Process File (RPF) to accompany accepted manuscripts.
This file will be published in conjunction with your paper and will include the anonymous referee reports, your point-by-point response and all pertinent correspondence relating to the manuscript. Let us know whether you agree with the publication of the RPF and as here, if you want to remove or not any figures from it prior to publication.
Please note that the Authors checklist will be published at the end of the RPF.

I look forward to receiving your revised manuscript.

Yours sincerely,

Lise Roth

Lise Roth, PhD
Senior Editor
EMBO Molecular Medicine

***** Reviewer's comments *****

Referee #1 (Comments on Novelty/Model System for Author):

The manuscript is technically strong, novel, and of significant translational relevance, identifying a previously unrecognized link between PRMT5 mediated intron retention and the generation of immunogenic tumour antigens. The combination of complementary mouse and human model systems is appropriate and supports the main conclusions. There are no major ethical concerns beyond standard animal and human sample use

Referee #1 (Remarks for Author):

The authors have addressed the concerns of this reviewer. I recommend publication.

Referee #2 (Remarks for Author):

The authors have addressed my concerns and I now recommend publication of this study.

Referee #3 (Comments on Novelty/Model System for Author):

See below. The slightly reduced score on novelty is due to the fact that they have published a similar analysis for peptides derived from lncRNAs before. The low score on medical impact is due to the reason detailed below. The authors portray the analysis as an argument for PRMT5 as target in immunotherapy and the effect size is very small.

Referee #3 (Remarks for Author):

This is a mechanistically interesting study and the authors have responded well to the comments.

What remains a major concern is the magnitude of the effect. The new Figure 1Aii shows that the overall effect of PRMT5 inhibition is minute. The new browser picture in S1B show three examples. For each, only one replicate and only two of the four experimental conditions are shown. Despite this selection for what are presumably the best examples, one of the effects is minute. In these conditions, the basal expression of all genes appears very different so some of the effects may be secondary and none is an E2F1 target. The data as presented do not establish with sufficient certainty that there is a significant effect of PRMT5 inhibition on splicing. It is therefore not clear enough, how the dsRNA observed in immunefluorescence emerges from these very small changes in splicing efficiency.

16th July 2026

EMM-2025-23176

Responses to the Reviewers from the Authors:

Referee #1 (Comments on Novelty/Model System for Author):

Referee #1 (Remarks for Author):

The authors have addressed the concerns of this reviewer. I recommend publication.

We thank Reviewer 1 for recommending acceptance of our revised manuscript and for their constructive and helpful comments throughout the review process.

Referee #2 (Remarks for Author):

The authors have addressed my concerns and I now recommend publication of this study.

We thank Reviewer 2 for recommending acceptance of our revised manuscript and for their constructive and helpful comments throughout the review process.

Referee #3 (Comments on Novelty/Model System for Author):

See below. The slightly reduced score on novelty is due to the fact that they have published a similar analysis for peptides derived from lncRNAs before. The low score on medical impact is due to the reason detailed below. The authors portray the analysis as an argument for PRMT5 as target in immunotherapy and the effect size is very small.

Referee #3 (Remarks for Author):

This is a mechanistically interesting study and the authors have responded well to the comments.

What remains a major concern is the magnitude of the effect. The new Figure 1Aii shows that the overall effect of PRMT5 inhibition is minute. The new browser picture in S1B show three examples. For each, only one replicate and only two of the four experimental conditions are shown. Despite this selection for what are presumably the best examples, one of the effects is minute. In these conditions, the basal expression of all genes appears very different so some of the effects may be secondary and none is an E2F1 target. The data as presented do not establish with sufficient certainty that there is a significant effect of PRMT5 inhibition on splicing. It is therefore not clear enough, how the dsRNA observed in immunefluorescence emerges from these very small changes in splicing efficiency.

Response to the Reviewer 3:
We thank the reviewer for highlighting this important point. We agree that PRMT5 inhibition produces relatively modest global changes in intron retention, and we have therefore substantially tempered our

mechanistic interpretation throughout the manuscript (please see lines 28-30, 95-96, 166-168, 186-187, 293-294). Specifically, we now distinguish the stronger evidence supporting a role for E2F1 in regulating intron retention from the broader effects of PRMT5 on innate immune activation. We also clarify that the J2 antibody detects dsRNA irrespective of its origin, and that retained introns likely represent one contributor to the observed dsRNA pool rather than its exclusive source (please see lines 233-234).

24th Jul 2026

Dear Prof. La Thangue,

Thank you for submitting your revised files. We are pleased to inform you that your manuscript is accepted for publication and is now being sent to our publisher to be included in the next available issue of EMBO Molecular Medicine.

Please note that the deposited datasets must be publicly available before online publication of the manuscript.

Your manuscript will be processed for publication by EMBO Press. It will be copy edited and you will receive page proofs prior to publication. Please note that you will be contacted by Springer Nature Author Services to complete licensing and payment information.

You may qualify for financial assistance for your publication charges - either via a Springer Nature fully open access agreement or an EMBO initiative. Check your eligibility: https://link.springer.com/journal/44321/how-to-publish-with-us

Should you be planning a Press Release on your article, please notify the production team via this form in order to coordinate publication and release dates: https://forms.cloud.microsoft/e/eH4A8JVP6g

If you have any questions, please do not hesitate to contact the Editorial Office. Thank you for your contribution to EMBO Molecular Medicine.

Yours sincerely,

Lise Roth

Lise Roth, Ph.D
Senior Editor
EMBO Molecular Medicine

>>> Please note that it is EMBO Molecular Medicine policy for the transcript of the editorial process (containing referee reports and your response letter) to be published as an online supplement to each paper. If you do NOT want this, you will need to inform the Editorial Office via email immediately. More information is available here: https://link.springer.com/partners/embo-press/editorial-policies#Peer%20review

EMBO Press Author Checklist

Corresponding Author Name: Nicholas La Thangue	
Journal Submitted to: EMBO Molecular Medicine	
Manuscript Number: EMM-2025-23176	

USEFUL LINKS FOR COMPLETING THIS FORM
The EMBO Journal - Author Guidelines
EMBO Reports - Author Guidelines
Molecular Systems Biology - Author Guidelines
EMBO Molecular Medicine - Author Guidelines

Reporting Checklist for Life Science Articles (updated January
This checklist is adapted from Materials Design Analysis Reporting (MDAR) Checklist for Authors. MDAR establishes a minimum set of requirements in transparent reporting in the life sciences (see Statement of Task: 10.31222/osf.io/9sm4x). Please follow the journal's guidelines in preparing your
Please note that a copy of this checklist will be published alongside your article.

Abridged guidelines for figures
1. Data
The data shown in figures should satisfy the following conditions:
- the data were obtained and processed according to the field's best practice and are presented to reflect the results of the experiments in an accurate and unbiased manner.
- ideally, figure panels should include only measurements that are directly comparable to each other and obtained with the same assay.
- plots include clearly labeled error bars for independent experiments and sample sizes. Unless justified, error bars should not be shown for technical
- if n<5, the individual data points from each experiment should be plotted. Any statistical test employed should be justified.
- Source Data should be included to report the data underlying figures according to the guidelines set out in the authorship guidelines on Data

2. Captions
Each figure caption should contain the following information, for each panel where they are relevant:
- a specification of the experimental system investigated (eg cell line, species name).
- the assay(s) and method(s) used to carry out the reported observations and measurements.
- an explicit mention of the biological and chemical entity(ies) that are being measured.
- an explicit mention of the biological and chemical entity(ies) that are altered/varied/perturbed in a controlled manner.
- the exact sample size (n) for each experimental group/condition, given as a number, not a range;
- a description of the sample collection allowing the reader to understand whether the samples represent technical or biological replicates (including how many animals, litters, cultures, etc.).
- a statement of how many times the experiment shown was independently replicated in the laboratory.
- definitions of statistical methods and measures:
  - common tests, such as t-test (please specify whether paired vs. unpaired), simple χ2 tests, Wilcoxon and Mann-Whitney tests, can be unambiguously identified by name only, but more complex techniques should be described in the methods section;
  - are tests one-sided or two-sided?
  - are there adjustments for multiple comparisons?
  - exact statistical test results, e.g., P values = x but not P values < x;
  - definition of 'center values' as median or average;
  - definition of error bars as s.d. or s.e.m.

Please complete ALL of the questions below.
Select "Not Applicable" only when the requested information is not relevant for your study.

Materials

Newly Created Materials	Information included in the manuscript?	In which section is the information available? (Reagents and Tools Table, Materials and Methods, Figures, Data Availability Section)
New materials and reagents need to be available; do any restrictions apply?	Not Applicable	

Antibodies	Information included in the manuscript?	In which section is the information available? (Reagents and Tools Table, Materials and Methods, Figures, Data Availability Section)
For **antibodies** provide the following information:<br>- Commercial antibodies: RRID (if possible) or supplier name, catalogue number and or/clone number<br>- Non-commercial: RRID or citation	Yes	Methods

DNA and RNA sequences	Information included in the manuscript?	In which section is the information available? (Reagents and Tools Table, Materials and Methods, Figures, Data Availability Section)
**Short novel DNA or RNA including primers, probes**: provide the sequences.	Yes	Methods

Cell materials	Information included in the manuscript?	In which section is the information available? (Reagents and Tools Table, Materials and Methods, Figures, Data Availability Section)
**Cell lines:** Provide species information, strain. Provide accession number in repository **OR** supplier name, catalog number, clone number, and/**OR** RRID.	Yes	Methods
**Primary cultures:** Provide species, strain, sex of origin, genetic modification status.	Not Applicable	
Report if the cell lines were recently **authenticated** (e.g., by STR profiling) and tested for mycoplasma contamination.	Yes	Methods

Experimental animals	Information included in the manuscript?	In which section is the information available? (Reagents and Tools Table, Materials and Methods, Figures, Data Availability Section)
**Laboratory animals or Model organisms:** Provide species, strain, sex, age, genetic modification status. Provide accession number in repository **OR** supplier name, catalog number, clone number, **OR** RRID.	Yes	Methods
**Animal observed in or captured from the field:** Provide species, sex, and age where possible.	Not Applicable	
Please detail **housing and husbandry conditions**.	Yes	Methods

Plants and microbes	Information included in the manuscript?	In which section is the information available? (Reagents and Tools Table, Materials and Methods, Figures, Data Availability Section)
**Plants:** provide species and strain, ecotype and cultivar where relevant, unique accession number if available, and source (including location for collected wild specimens).	Not Applicable	
**Microbes:** provide species and strain, unique accession number if available, and source.	Not Applicable	

Human research participants	Information included in the manuscript?	In which section is the information available? (Reagents and Tools Table, Materials and Methods, Figures, Data Availability Section)
If collected and within the bounds of privacy constraints report on age, sex and gender or ethnicity for all study participants.	Yes	Methods

Core facilities	Information included in the manuscript?	In which section is the information available? (Reagents and Tools Table, Materials and Methods, Figures, Data Availability Section)
If your work benefited from core facilities, was their service mentioned in the acknowledgments section?	Yes	Methods

Design

Study protocol	Information included in the manuscript?	In which section is the information available? (Reagents and Tools Table, Materials and Methods, Figures, Data Availability Section)
If study protocol has been **pre-registered, provide DOI in the manuscript**. For clinical trials, provide the trial registration number **OR** cite DOI.	Not Applicable	
Report the **clinical trial registration number** (at ClinicalTrials.gov or equivalent), where applicable.	Not Applicable	

Laboratory protocol	Information included in the manuscript?	In which section is the information available? (Reagents and Tools Table, Materials and Methods, Figures, Data Availability Section)
Provide DOI OR other citation details if **external detailed step-by-step protocols** are available.	Not Applicable	

Experimental study design and statistics	Information included in the manuscript?	In which section is the information available? (Reagents and Tools Table, Materials and Methods, Figures, Data Availability Section)
Include a statement about **sample size** estimate even if no statistical methods were used.	Yes	Methods
Were any steps taken to minimize the effects of subjective bias when allocating animals/samples to treatment (e.g. **randomization procedure**)? If yes, have they been described?	Yes	Methods
Include a statement about **blinding** even if no blinding was done.	Yes	Methods
Describe **inclusion/exclusion criteria** if samples or animals were excluded from the analysis. Were the criteria pre-established? If sample or data points were omitted from analysis, report if this was due to attrition or intentional exclusion and provide justification.	Yes	Methods, Statistical analysis
For every figure, are **statistical tests** justified as appropriate? Do the data meet the assumptions of the tests (e.g., normal distribution)? Describe any methods used to assess it. Is there an estimate of variation within each group of data? Is the variance similar between the groups that are being statistically compared?	Yes	Methods, Figure Legends

Sample definition and in-laboratory replication	Information included in the manuscript?	In which section is the information available? (Reagents and Tools Table, Materials and Methods, Figures, Data Availability Section)
In the figure legends: state number of times the experiment was **replicated** in laboratory.	Yes	Figure legends
In the figure legends: define whether data describe **technical or biological replicates**.	Yes	Figure legends

Ethics

Ethics	Information included in the manuscript?	In which section is the information available? (Reagents and Tools Table, Materials and Methods, Figures, Data Availability Section)
Studies involving **human participants**: State details of **authority granting ethics approval** (IRB or equivalent committee(s), provide reference number for approval.	Yes	Methods
Studies involving **human participants**: Include a statement confirming that **informed consent** was obtained from all subjects and that the experiments conformed to the principles set out in the WMA Declaration of Helsinki and the Department of Health and Human Services Belmont Report.	Yes	Methods
Studies involving **human participants:** For publication of **patient photos**, include a statement confirming that consent to publish was obtained.	Not Applicable	
Studies involving experimental **animals**: State details of **authority granting ethics approval** (IRB or equivalent committee(s), provide reference number for approval. Include a statement of compliance with ethical regulations.	Yes	Methods
Studies involving **specimen and field samples:** State if relevant **permits** obtained, provide details of authority approving study; if none were required, explain why.	Not Applicable	

Dual Use Research of Concern (DURC)	Information included in the manuscript?	In which section is the information available? (Reagents and Tools Table, Materials and Methods, Figures, Data Availability Section)
Could your study fall under dual use research restrictions? Please check biosecurity documents and list of **select agents and toxins** (CDC): https://www.selectagents.gov/sat/list.htm	Not Applicable	
If you used a select agent, is the security level of the lab appropriate and reported in the manuscript?	Not Applicable	
If a study is subject to dual use research of concern regulations, is the name of the **authority granting approval and reference number** for the regulatory approval provided in the manuscript?	Not Applicable	

Reporting

The MDAR framework recommends adoption of discipline-specific guidelines, established and endorsed through community initiatives. Journals have their own policy about requiring specific guidelines and recommendations to complement MDAR.

Adherence to community standards	Information included in the manuscript?	In which section is the information available? (Reagents and Tools Table, Materials and Methods, Figures, Data Availability Section)
State if relevant guidelines or checklists (e.g., **ICMJE, MIBBI, ARRIVE, PRISMA**) have been followed or provided.	Not Applicable	
For **tumor marker prognostic studies,** we recommend that you follow the **REMARK** reporting guidelines (see link list at top right). See author guidelines, under 'Reporting Guidelines'. Please confirm you have followed these guidelines.	Not Applicable	
For **phase II and III randomized controlled trials**, please refer to the **CONSORT** flow diagram (see link list at top right) and submit the CONSORT checklist (see link list at top right) with your submission. See author guidelines, under 'Reporting Guidelines'. Please confirm you have submitted this list.	Not Applicable	

Data Availability

Data availability	Information included in the manuscript?	In which section is the information available? (Reagents and Tools Table, Materials and Methods, Figures, Data Availability Section)
Have **primary datasets** been deposited according to the journal's guidelines (see 'Data Deposition' section) and the respective accession numbers provided in the Data Availability Section?	Yes	Methods, Data Availability
Were **human clinical and genomic datasets** deposited in a public access-controlled repository in accordance to ethical obligations to the patients and to the applicable consent agreement?	Not Applicable	
Are **computational models** that are central and integral to a study available without restrictions in a machine-readable form? Were the relevant accession numbers or links provided?	Not Applicable	
If publicly available data were reused, provide the respective **data citations in the reference list**.	Yes	Methods
